# Recent Advances in Near-Infrared Fluorescent Probes Based on Organic Small Molecules for the Detection and Imaging of Heavy Metal Ions

**DOI:** 10.3390/molecules31203621

**Published:** 2026-10-11

**Authors:** Luyi Zong, Jun Zhang, Xianchao Du, Xiaojing Xing, Dongfang Qiu

**Affiliations:** College of Chemistry and Pharmaceutical Engineering, Nanyang Normal University, Nanyang 473061, China

**Keywords:** near-infrared fluorescent probes, organic small molecules, heavy metal ions, imaging

## Abstract

Heavy metal pollution poses a severe threat to ecosystems and human health. Therefore, it is important to develop rapid, sensitive, and reliable detection methods. Fluorescent probes with near-infrared (NIR, 650–900 nm) emission have emerged as powerful analytical tools owing to their distinct advantages, including low background interference, deep-tissue penetration, minimal photodamage, and high signal-to-noise ratios, which make them particularly attractive for both environmental monitoring and bioimaging applications. This review systematically summarizes recent progress in NIR organic small-molecule fluorescent probes for the detection of heavy metal ions, covering the period from 2022. Representative examples are categorized and discussed, with a focus on key metal ions such as Cu^2+^, Hg^2+^, Cd^2+^, Zn^2+^, Pb^2+^, Fe^2+^ and Fe^3+^. The molecular design strategies, sensing performance, and practical applications in environmental samples, food samples and biological imaging are critically discussed. Furthermore, current challenges and future opportunities in this rapidly advancing field are highlighted.

## 1. Introduction

With the rapid development of modern industrialization and urbanization, heavy metal pollution has become one of the most severe global environmental problems. Heavy metal ions such as Hg^2+^, Pb^2+^, Cd^2+^, Fe^2+^/Fe^3+^, and Cu^2+^/Cu^+^ are released into the environment through various pathways, including mineral mining, metal smelting, electronic waste disposal, industrial wastewater discharge, and pesticide use, posing serious threats to ecosystems and human health. Owing to their characteristics of high toxicity, non-biodegradability and bioaccumulation, even at trace levels, heavy metal ions can accumulate in living organisms through the food chain, ultimately leading to various severe effects to human beings [1,2]. For example, excessive Cu^2+^ in the human body can induce oxidative stress, leading to gastrointestinal dysfunction and neurological damage. Chronic copper overload may affect the liver, brain, and kidneys, producing clinical manifestations resembling Wilson disease, including cirrhosis, tremor, and neuropsychiatric symptoms [3,4]. Hg^2+^ overload can cause irreversible damage to multiple organs and systems, including the kidneys, heart, central nervous system, cardiovascular system, and reproductive system. In particular, methylmercury (CH_3_Hg^+^) readily crosses the blood–brain barrier and poses a particular threat to fetal and childhood neurodevelopment [5,6]. Zinc overload can result in multisystem toxicity, primarily manifesting as gastrointestinal disturbances, immune dysfunction, neurological damage, and dyslipidemia, thereby increasing the risk of atherosclerosis; it may also disrupt cardiac electrophysiological activity and contribute to arrhythmias [7]. Following entry into the human body, Cd^2+^ predominantly accumulates in the kidneys and bones. Excessive cadmium exposure can cause renal tubular injury, disturbances in calcium and phosphorus metabolism, osteoporosis, and osteomalacia, and in severe cases may progress to Itai-Itai disease. Chronic exposure is also associated with a significantly increased risk of malignancies, including lung and prostate cancers [8]. Lead poisoning can cause severe multisystem damage, primarily affecting the hematopoietic, nervous, digestive, urinary, cardiovascular, and immune systems [9]. Excessive iron intake can irritate the gastrointestinal tract, increase the risk of cardiovascular diseases, cause liver injury, and induce tumorigenesis [10,11]. Thus, the World Health Organization (WHO) and the United States Environmental Protection Agency (EPA) have set strict limits on the concentration of various heavy metal ions in drinking water. For example, the WHO has established a maximum allowable concentration of 0.5 μg/L for Hg^2+^ [12], 5 μg/L for Cd^2+^ [13], and 10–15 mg/L for Pb^2+^ [14]. Thus, the development of rapid, sensitive and reliable technology to detect heavy metal ions has significant social and economic significance for environmental monitoring, food safety and public health protection.

Traditional detection methods for monitoring heavy metal ions include atomic absorption spectrometry (AAS), atomic emission spectrometry (AES), inductively coupled plasma mass spectrometry (ICP-MS) and electrochemical analysis [15,16,17,18]. Although these methods have high sensitivity and accuracy, they have inherent limitations including high instrument costs, complex pre-processing procedures, time-consuming operations, and the inability to be utilized for in situ visualization of biological imaging, which limit their application in a wide range of scenarios [19,20].

**Figure 1:**
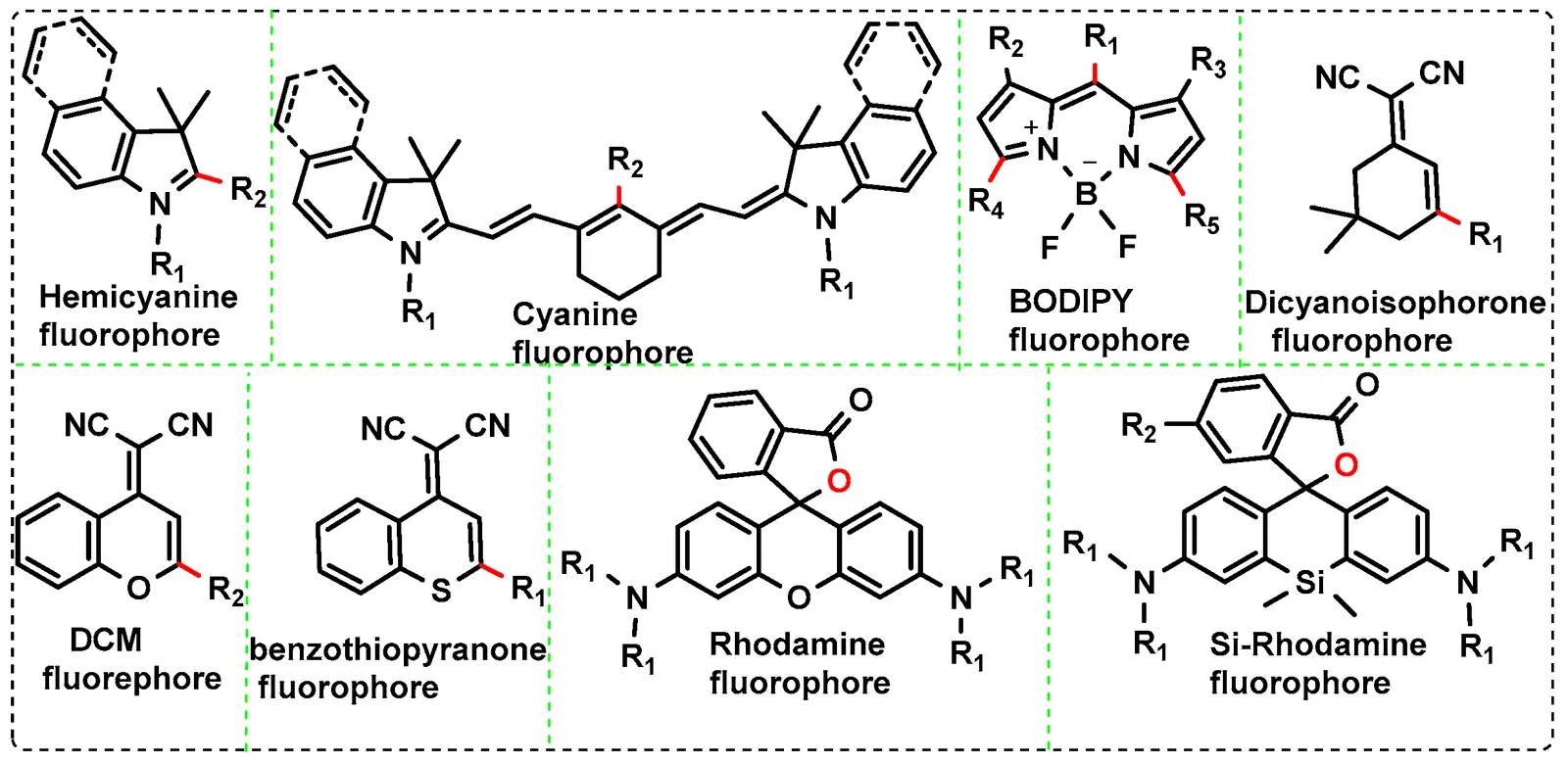
General structures of commonly used NIR fluorophores.

The field of fluorescent probes began with the pioneering work of Minta et al., who reported the first Cu^2+^-selective fluorescent probe in 1989. Since then, thousands of fluorescent probes have been developed [21,22,23,24,25,26,27]. Compared with traditional methods, fluorescent probes are characterized by their high selectivity and sensitivity, real-time detection, low cost and non-invasive imaging, making them attractive tools in life sciences, environmental monitoring, and medical diagnostics, particularly for ion detection, bioimaging, and drug delivery [28,29,30,31]. Most fluorescent probes emit in the visible region (λ_em_ < 600 nm), making them susceptible to background fluorescence, tissue absorption and strong light scattering during application. In comparison, near-infrared fluorescent probes (NIR, 650–900 nm) offer the benefits of minimal background fluorescence, lower light scattering, deep-tissue imaging capability, and high imaging contrast [32,33,34,35,36]. Commonly used NIR fluorophores include BODIPYs [37,38,39,40], hemicyanines [41,42,43,44], cyanines [45], dicyanoisophorone analogues [46,47,48,49], DCM derivatives [50,51,52], rhodamines [53,54], etc. (Figure 1). These dyes generally exhibit large Stokes shifts, tunable structures, and pronounced spectral changes upon interaction with analytes. Owing to these advantages, NIR fluorescence imaging has gained considerable attention over the past decade, with numerous NIR-emitting materials being applied to detect intracellular active species, analyze tumor biomarkers, monitor disease progression, evaluate drug efficacy in vivo, and more recently, to address challenges in environmental and food safety monitoring.

In recent years, there have been several reviews covering the developments in NIR fluorescent probes, focusing primarily on the sensing and bioimaging of small molecules (e.g., biothiols, H_2_S) [55,56] and biomacromolecules (e.g., proteins, enzymes, nucleic acids) [57,58,59], as well as their applications in cancer targeting and disease diagnosis (e.g., Alzheimer’s disease, kidney injury, urological disorders) [60,61,62,63]. For example, Yu, Cong and co-workers (2025) systematically outlined advances in NIR small-molecule probes for responsive biomarkers from 2018 to 2025, emphasizing their roles in cancer research and therapy [64]. Wang et al. (2025) comprehensively reviewed NIR fluorescent probes for detecting Alzheimer’s disease biomarkers, categorizing them by dye structure, for instance curcumin, BODIPY, quinoline, and donor acceptor systems, and discussing their spectral characteristics, in vitro detection, and in vivo imaging performance [65]. Additionally, many reviews have addressed design strategies based on specific fluorophore platforms (e.g., heptamethine cyanines, coumarins, rhodamines, silicon-substituted xanthenes) and their sensing and bioimaging applications [66,67,68,69,70]. Pu et al. (2021) systematically summarized recent advances in NIR probes built upon a hemicyanine skeleton for disease imaging and diagnosis [71], while Guo et al. (2025) highlighted hemicyanine-based NIR probes for detecting key disease-related biomarkers and their utility in diagnosis and treatment [72].

Despite these contributions, a comprehensive and dedicated review that systematically described the construction strategies, analytical performance, response mechanisms, and bioimaging utility of NIR fluorescent probes for heavy metal ions is still lacking. Over the past few years, remarkable progress has been made in the detection and bioimaging of ions such as Cu^2+^, Hg^2+^, Cd^2+^, Zn^2+^, Pb^2+^ and Fe^2+^/Fe^3+^ using NIR probes. Hence, a comprehensive and timely survey is urgently required to guide future developments in this vibrant research domain.

Here, we comprehensively reviewed the research progress on NIR fluorescent probes for heavy metal ions reported since 2022. This review introduces the common sensing mechanisms employed by these probes. Then, the probes are classified by the target metal ions, focusing on molecular design strategies, sensing behaviors, recognition mechanisms, and practical applications. Finally, based on an in-depth analysis of the sensing performance and application scope of the reported NIR probes, we identify the existing challenges and future directions in this rapidly evolving field. This review is expected to provide valuable guidance for the rational design of next-generation NIR fluorescent probes for the detection of heavy metal ions in complex environmental and biological systems.

## 2. Sensing Mechanism of Fluorescent Probes for Heavy Metal Ions

There are various mechanisms by which fluorescent probes detect heavy metal ions, including photo-induced electron transfer (PET), intramolecular charge transfer (ICT), twisted intramolecular charge transfer (TICT), fluorescence resonance energy transfer (FRET), excited-state intramolecular proton transfer (ESIPT), chelation-enhanced fluorescence (CHEF) or quenching (CHEQ), aggregation-induced emission (AIE), ligand-to-metal charge transfer (LMCT), metal-to-ligand charge transfer (MLCT), through-bond energy transfer (TBET), and so on. The following sections will detail the mechanisms most frequently employed in near-infrared fluorescent probes for heavy metal ion sensing, which are also extensively discussed in key review articles [73,74].

### 2.1. Photo-Induced Electron Transfer (PET)

**Figure 2:**
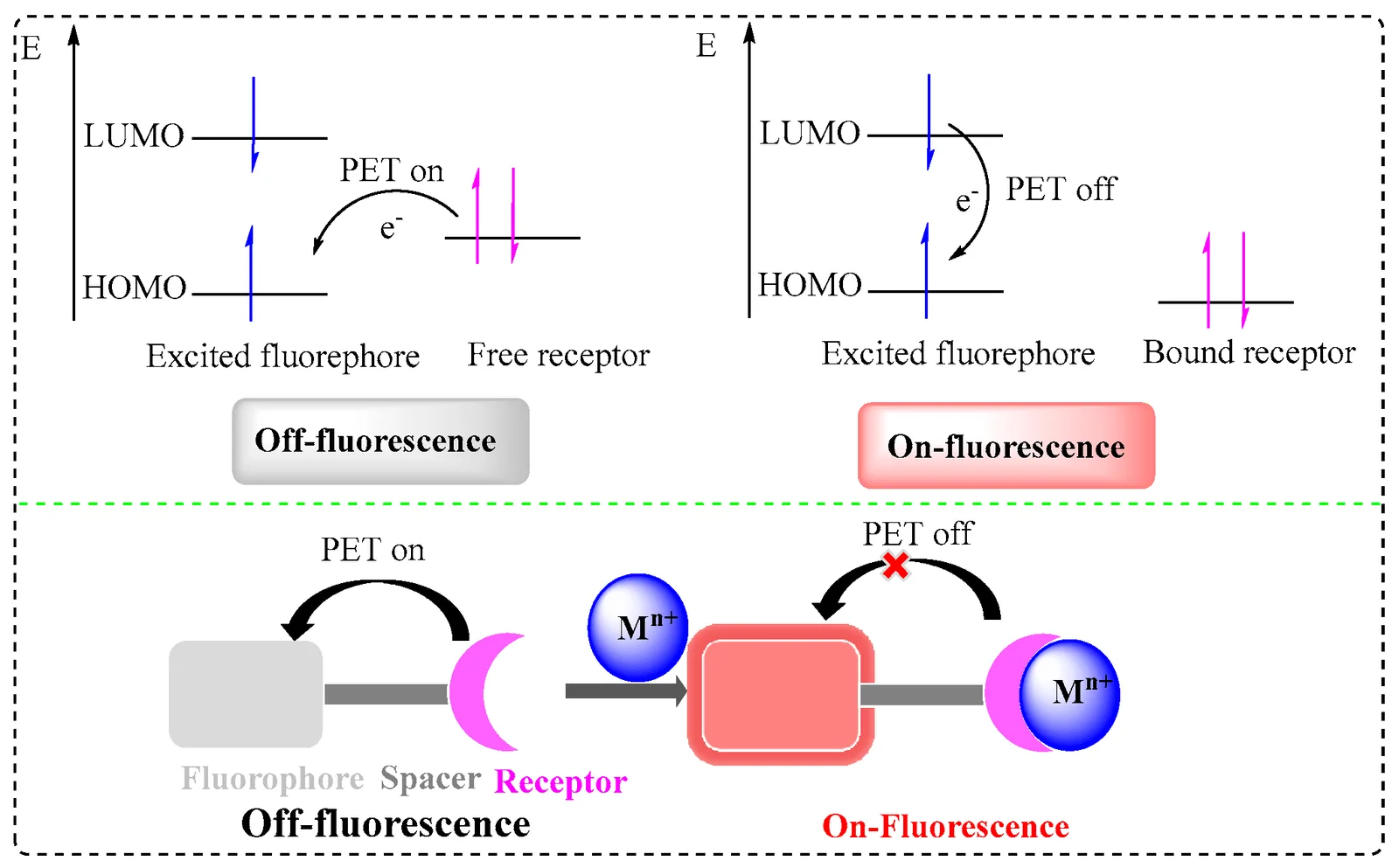
Mechanism of photo-induced electron transfer (PET).

Fluorescent probes based on the PET mechanism typically consist of three key components: a fluorophore, a linker, and a recognition group. In such a system, the fluorophore serves as an electron acceptor, while the recognition unit functions as an electron donor. A critical feature of this design is that the highest occupied molecular orbital (HOMO) energy level of the recognition unit (electron donor) lies between the HOMO and the lowest unoccupied molecular orbital (LUMO) energy levels of the fluorophore (electron acceptor) (Figure 2). Upon photoexcitation of the fluorophore, an electron is promoted from its HOMO to its LUMO, creating a vacancy in the HOMO of the fluorophore that is subsequently filled by an electron from the HOMO of the receptor. This inter-component electron transfer process, namely the PET process, effectively quenches the fluorescence of the fluorophore. When a target cation binds to the recognition unit, the HOMO energy level of the donor is lowered. It reaches a lower level than that of the fluorophore, thereby suppressing the PET pathway and restoring the fluorescence emission. This is a characteristic signal “turn-on” response.

### 2.2. Intramolecular Charge Transfer (ICT)

**Figure 3:**
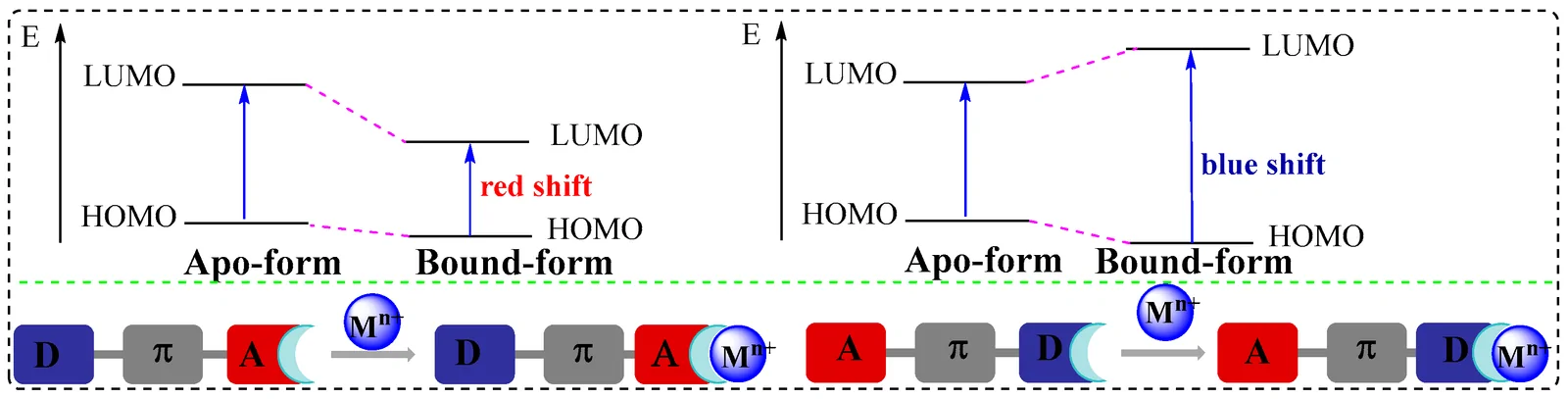
The mechanism of intramolecular charge transfer (ICT).

Typically, ICT-based fluorescent probes are composed of an electron-donating moiety and an electron-withdrawing moiety linked via a conjugated bridge, thereby creating a “push–pull” electronic system (Figure 3). Upon binding of the recognition unit to the target analyte, the electron-donating or electron-withdrawing nature of the system is modulated, leading to readjustment of the charge distribution throughout the entire conjugated system. Consequently, the “push–pull” effect is either enhanced or weakened, producing a spectral shift (either red or blue) in absorption or fluorescence. This mechanism is often used to design ratiometric fluorescence probes.

### 2.3. Fluorescence Resonance Energy Transfer (FRET)

**Figure 4:**
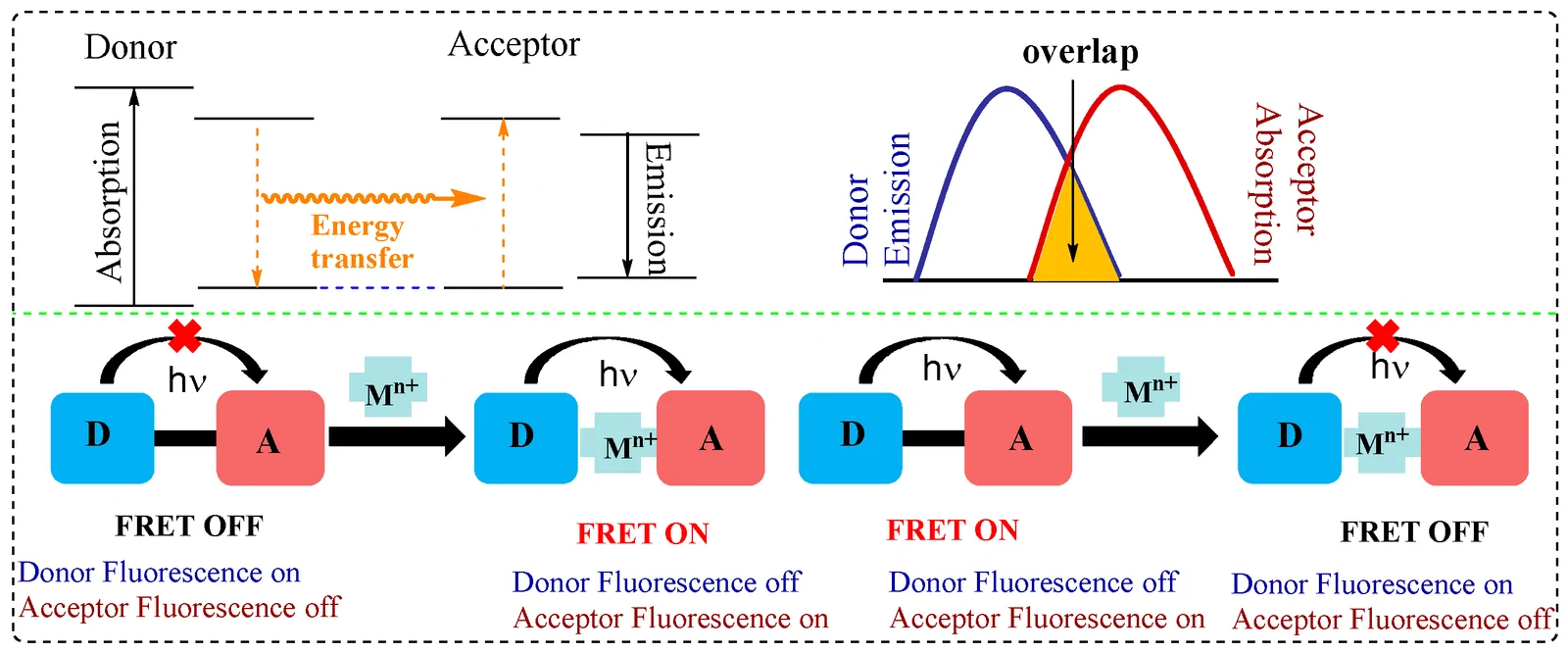
The mechanism of fluorescence resonance energy transfer (FRET). D: donor; A: acceptor; hn: energy.

FRET is another frequently employed mechanism for constructing ratiometric fluorescent probes. Such probes typically consist of two fluorophores, serving as the energy donor–acceptor pair. The core principle of FRET relies on sufficient spectral overlap between donor emission and acceptor absorption, with their separation generally falling within 10–100 Å (Figure 4). In the absence of energy transfer, donor emission is predominantly observed. Upon target recognition, the FRET pathway is activated, causing an increase in acceptor emission and a corresponding decline in donor emission. Conversely, if the probe is initially in a FRET-active state, fluorescence from the acceptor predominates. Interaction with the analyte can inhibit the FRET progress, causing recovery of donor fluorescence and attenuation of acceptor emission.

### 2.4. Chelation-Enhanced Fluorescence (CHEF)

**Figure 5:**
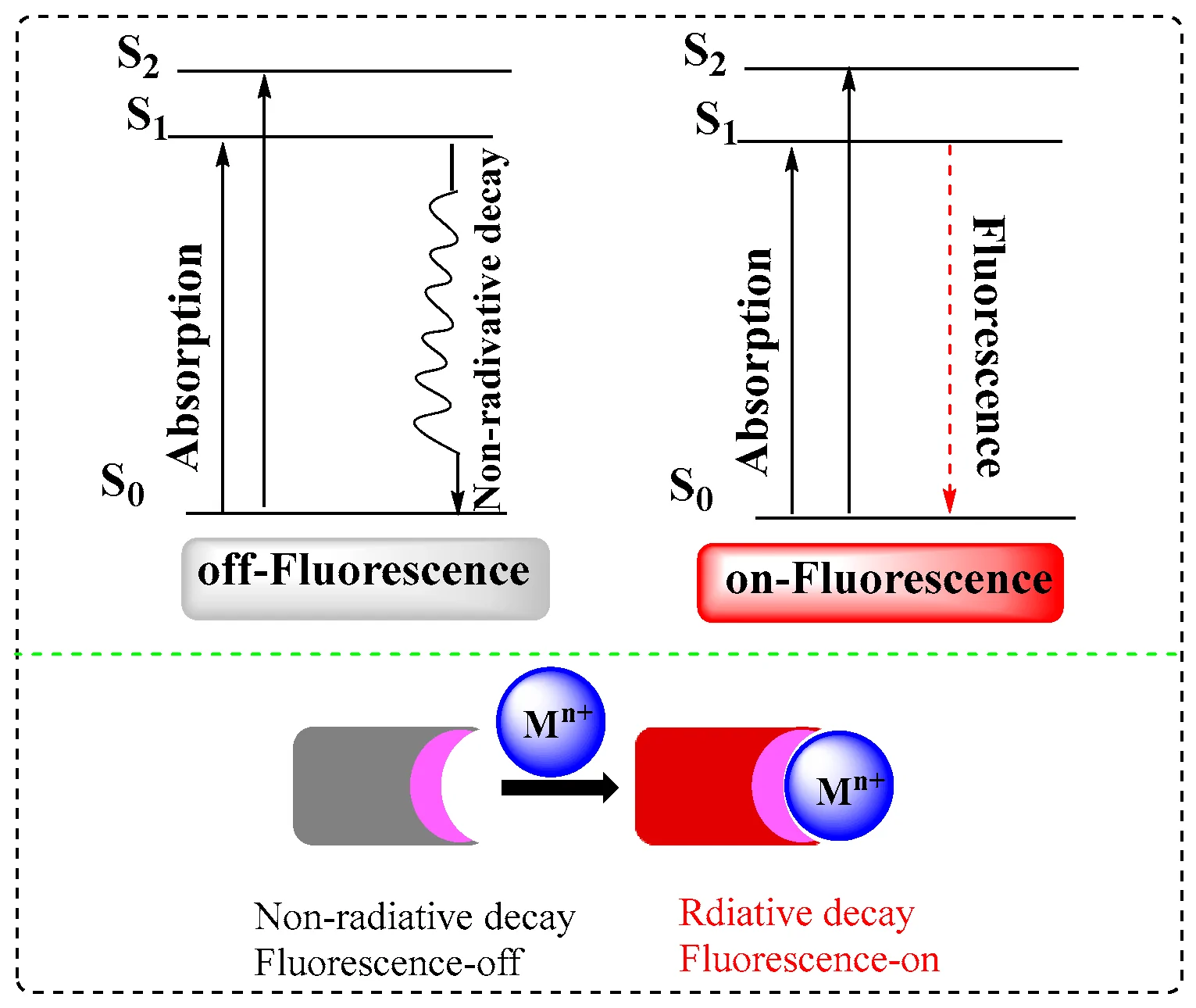
The mechanism of chelation-enhanced fluorescence (CHEF).

CHEF serves as a crucial photophysical pathway for fluorescence sensing. In such probes, the fluorophore or receptor unit typically incorporates heteroatoms with lone-pair electrons (such as, N, O, and S) (Figure 5). Upon excitation, the probes undergo non-radiative decay through molecular vibrations, rotations, or electronic energy transfer, leading to fluorescence quenching or very weak emission. Upon specific coordination with the target metal ion (M^n+^), the lone-pair electrons become engaged in bond formation, which rigidifies the local conformation of the recognition site. Consequently, the radiative transition pathway is restored or significantly enhanced, generating an intense fluorescence signal (“turn-on” response).

## 3. NIR Fluorescent Probes for Heavy Metal Ions

### 3.1. NIR Fluorescent Probes for Cu^2+^

Copper (Cu^2+^) plays dual roles as both an essential trace element and a significant environmental pollutant. Anthropogenic activities, such as industrial manufacturing and agriculture, release substantial amounts of copper into the ecosystems. Cu^2+^ accumulates in water and soil and is subsequently enriched via the food chain, thereby constituting a significant hazard to public health [75]. Biologically, Cu^2+^ is an indispensable co-factor in numerous enzymes critical for hematopoiesis, neural function, development, and antioxidant defense [76]. Consequently, both deficiency and overload disrupt cellular homeostasis, leading to severe disorders, including Menkes and Wilson’s diseases, neurodegeneration, and oxidative damage, underscoring the critical importance of maintaining copper homeostasis [77]. Given its environmental prevalence and profound biological impact, developing reliable monitoring tools is essential. Leveraging their deep penetration into tissues and minimal background signals, NIR fluorescent probes offer a promising approach for the real-time detection of Cu^2+^ in complicated biological and environmental specimens. During the period of 2022–2026, diverse NIR fluorescent probes were successfully developed for Cu^2+^ sensing. Based on dyes including rhodamine, isophorone, heptamethine cyanine, and so on, these probes demonstrate outstanding sensing capabilities. The photophysical properties of representative probes are summarized in Table 1.

#### 3.1.1. NIR Fluorescent Probes Derived from Dicyanoisohporone Derivatives

**Figure 6:**
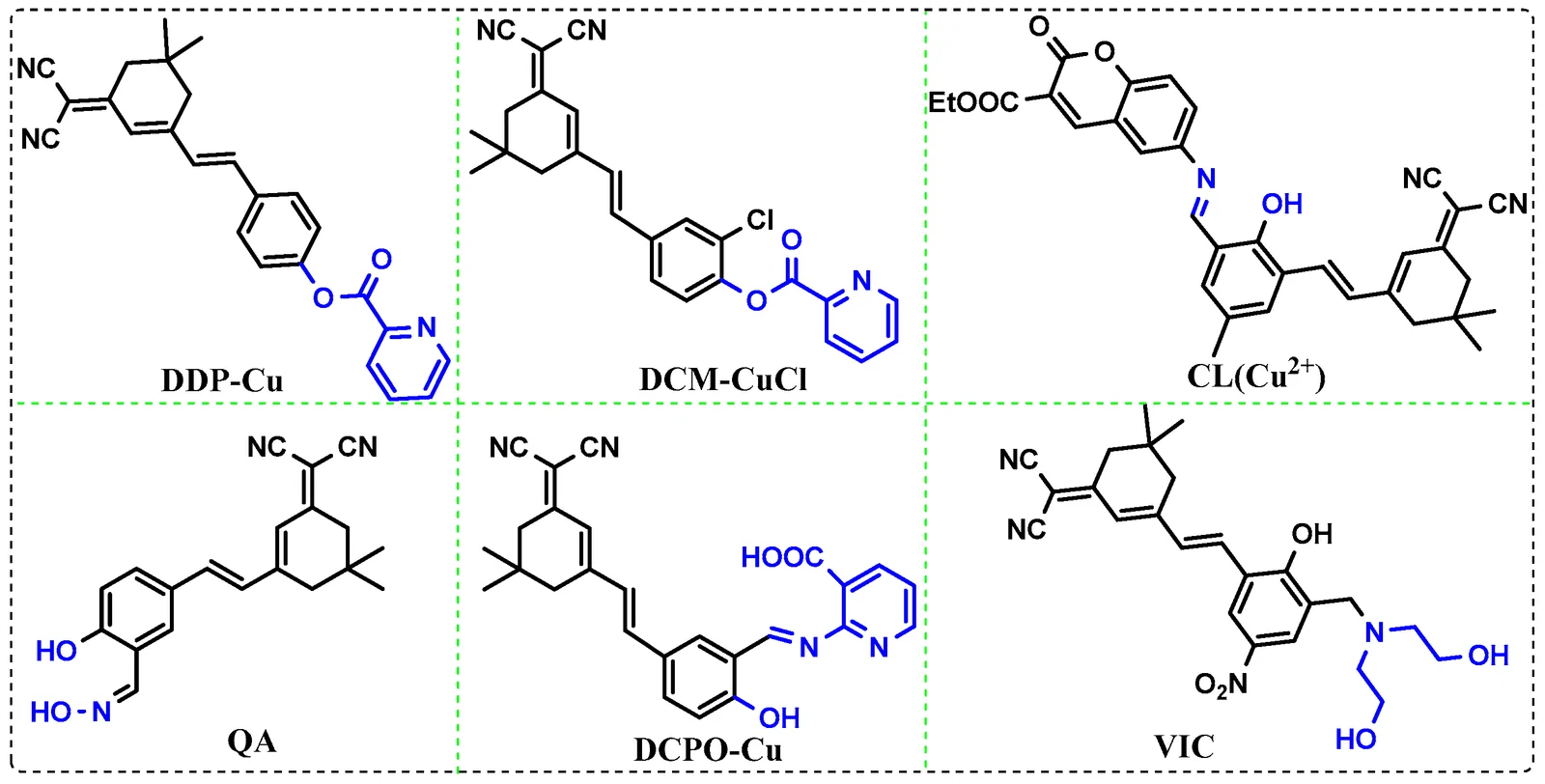
Structures of NIR fluorescent probes for Cu^2+^ based on dicyanoisohporone platform.

Dicyanoisophorone derivatives, owing to their unique D-π-A structure, exhibit excellent fluorescence properties in the NIR region, such as tunable emission wavelengths, large Stokes shifts, and good photostability. Thus, they have emerged as an ideal platform for designing Cu^2+^ fluorescent probes. To date, numerous NIR fluorescent probes (Figure 6) have been fabricated by introducing different specific recognition groups for Cu^2+^ to the dicyanoisophorone skeleton [78,79,80,81,82,83].

In 2022, Zhou et al. introduced a 2-pyridinecarbonyl group as a recognition unit and initial fluorescence quencher into the dicyanoisophorone platform to construct the probe **DDP-Cu** [78]. Owing to the interrupted ICT process, the probe was initially in a fluorescence-off state. Upon Cu^2+^-induced hydrolysis of the picolinate ester, **DDP-Cu** was converted to the NIR fluorophore with restored ICT, producing a strong emission at 665 nm (LOD = 36 nM). **DDP-Cu** enabled the imaging and quantification of Cu^2+^ in live cells, zebrafish, and brain tissues of AD model mice. Fluorescence imaging revealed that Cu^2+^ levels in AD brains increased 3.5-fold (mice) and 4-fold (Drosophila) and further uncovered the connections between elevated Cu^2+^, oxidative stress, and Aβ aggregation, making it a promising tool for studying Cu^2+^-related AD pathology.

In 2023, Zhang et al. incorporated a chlorine atom into the picolinate recognition moiety, obtaining the probe **DCM-CuCl** [79]. The introduction of a chloride substituent effectively accelerated the Cu^2+^-catalyzed hydrolysis reaction, shortening the response time to 2.5 min, while maintaining NIR emission at 668 nm. **DCM-CuCl** was effectively used for Cu^2+^ visualization in HeLa cells and for the assay of Cu^2+^ in real water samples, demonstrating the positive effect of chlorine modification on enhancing the reaction kinetics.

As research progressed, the strategic incorporation of appropriate recognition moieties onto the dicyanoisophorone framework led to the construction of multifunctional fluorescent probes that can simultaneously sense Cu^2+^ and other biologically or environmentally relevant analytes. In 2025, Li’s group created a coumarin–dicyanoisophorone-based probe **CL(Cu^2+^)**, capable of simultaneously responding to lysine (Lys), Cu^2+^, Fe^3+^, and biothiols [80]. The coordination of Cu^2+^ involved the phenolic oxygen and imine nitrogen of **CL(Cu^2+^)**, blocking ICT and causing fluorescence quenching at 704 nm (LOD = 1.70 mM). The resulting **CL(Cu^2+^)-Cu^2+^** complex could further undergo competitive coordination with biothiols like glutathione (GSH), restoring fluorescence and enabling the secondary detection of GSH. **CL(Cu^2+^)** exhibited good biocompatibility in live-cell imaging.

Dong’s team reported a probe **QA**, featuring aggregation-induced emission (AIE) characteristics [81]. The oxime and phenolic hydroxyl groups in its structure coordinated with Cu^2+^ in a 2:1 stoichiometry, causing a fluorescence turn-off at 688 nm. Upon subsequent addition, GSH exhibited a stronger binding affinity for Cu^2+^ than **QA**, thereby displacing the metal ion from the **QA**-Cu^2+^ complex and liberating the free probe, which led to fluorescence recovery. The **QA**-Cu^2+^ complex demonstrated high selectivity for GSH, with an LOD of 0.347 μM. **QA** exhibited excellent selectivity, high sensitivity, strong anti-interference capability, and rapid response toward Cu^2+^ and GSH. The probe has been successfully applied to the quantitative determination of Cu^2+^ in tap water and lake water samples with satisfactory spiked recoveries. Furthermore, in combination with RGB color analysis and a smartphone platform, the probe enabled convenient on-site quantitative detection of Cu^2+^ with an LOD of 40.4 mM. In addition, **QA** was effectively applied to visualize Cu^2+^ and GSH in HepG-2 cells, C. elegans, and mice, indicating the advantages of AIE probes in biological research.

While the aforementioned isophorone-based probes have primarily been employed for Cu^2+^ detection under controlled laboratory conditions, recent efforts have shifted toward the development of practical tools that enable rapid, on-site monitoring of Cu^2+^ in real-world settings. In 2025, Li et al. synthesized the probe **DCPO-Cu** using 2-aminonicotinic acid as the responsive group for Cu^2+^ [82]. Cu^2+^ exclusively triggered fluorescence quenching at 655 nm, whereas other common metal ions and anions had negligible effects. Moreover, the coexistence of these ions did not compromise the probe’s response to Cu^2+^, highlighting its superior selectivity and anti-interference performance. **DCPO-Cu** not only enabled sensitive detection of Cu^2+^ in water samples, food (rice, mung beans) and plant tissues (onion roots), but was also further adapted into convenient test strips for field use. Additionally, combined with a smartphone color recognition APP, it achieved on-site quantitative analysis of Cu^2+^ in soil samples. However, the fluorescence quenching mechanism restricted its use for live-cell imaging, underscoring the need for further development of probes tailored to biomedical applications.

Zhang et al. constructed the probe **VIC** using diethanolamine as the recognition unit [83]. The N and O atoms in diethanolamine, along with the phenolic hydroxyl group, coordinated with Cu^2+^, blocking ICT and causing fluorescence quenching at 675 nm. **VIC** demonstrated excellent sensing performance for Cu^2+^, including fast response (30 s), ultra-high sensitivity (LOD = 7 nM), excellent selectivity, and wide pH adaptability (5.0–12.0), enabling the rapid assessment of Cu^2+^ in soil samples. Furthermore, the probe has been successfully fabricated into a smartphone-assisted portable test kit for rapid, on-site detection of Cu^2+^ residues on the surface of traditional Chinese medicines (senna alexandrina mill) and vegetables (reed leaves, Chinese chives and garlic bolts) with satisfactory recoveries (89.5–108.7%). With the advantages of low cytotoxicity and good biocompatibility, **VIC** can image exogenous Cu^2+^ in the endoplasmic reticulum of MCF-7 cells, as well as in live zebrafish and nude mice. Notably, **VIC** enabled three-dimensional fluorescence imaging of AD mouse brains, revealing that Cu^2+^ levels progressively increased with disease progression, highlighting its potential for studying Cu^2+^-related neurodegenerative diseases.

#### 3.1.2. NIR Fluorescent Probes Derived from Rhodamine Derivatives

**Figure 7:**
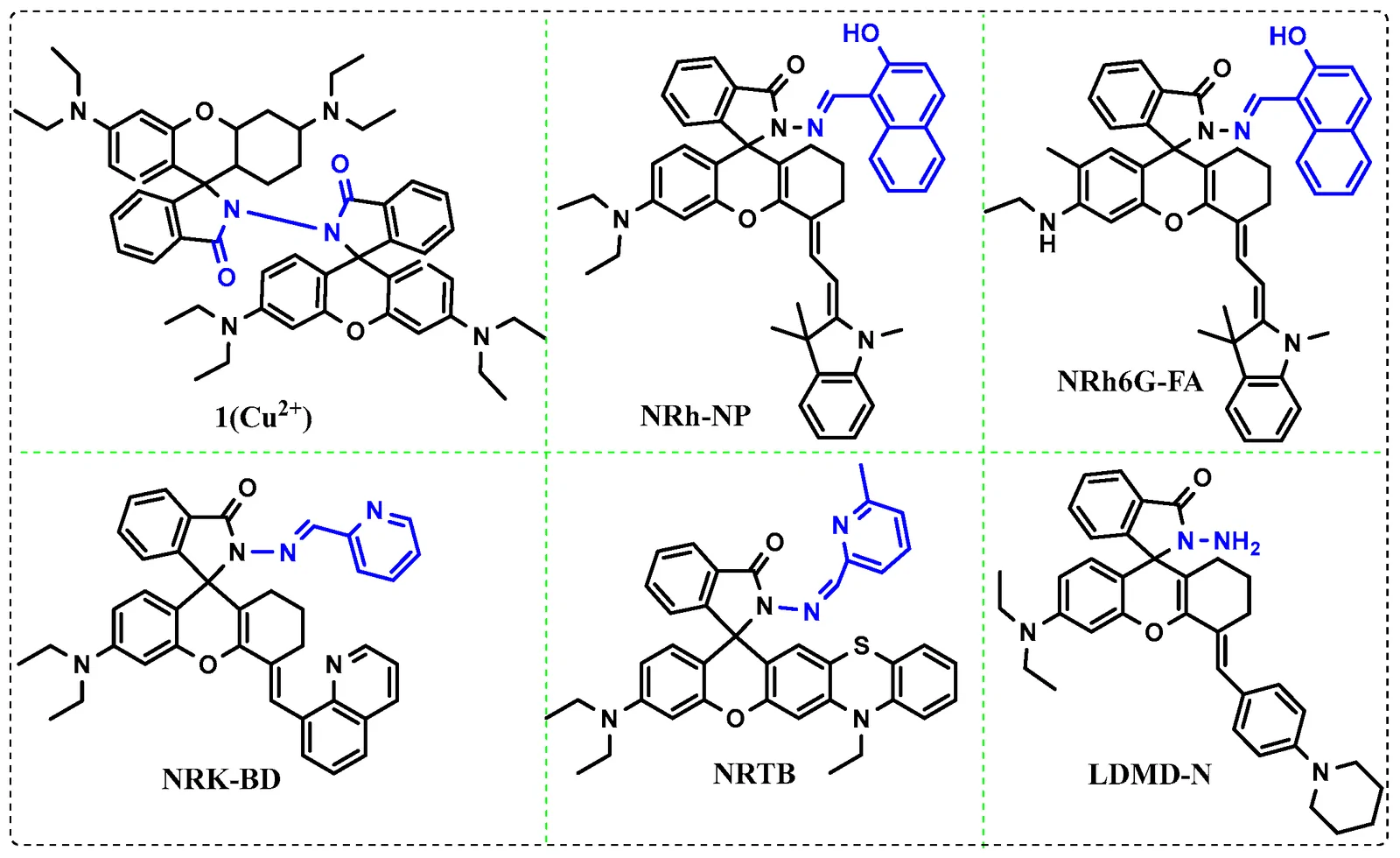
Structures of NIR fluorescent probes for Cu^2+^ based on rhodamine skeleton.

Owing to their unique spirolactam structure, high fluorescence quantum yields, excellent photostability and large molar extinction coefficients, rhodamine derivatives have become an ideal platform for designing “turn-on” fluorescent probes. Classical rhodamines are typically colorless and have a non-fluorescent spirocyclic form. Upon binding to specific analytes (such as metal ions), the spirolactam ring opens, leading to significant fluorescence enhancement and color change. This mechanism has been widely exploited for the highly selective detection of Cu^2+^. However, conventional rhodamine-based probes generally emit in the visible region (550–590 nm) and have small Stokes shifts (20–40 nm), which limits their utility in deep-tissue imaging. To overcome this limitation, a series of novel rhodamine-based Cu^2+^ probes (Figure 7) with emission wavelengths red-shifted to the NIR region and large Stokes shifts have been developed through strategies such as extending the π-conjugation system, introducing strong electron-donating groups, or constructing a hybrid scaffold [84,85,86,87,88,89].

For example, based on the mechanism mentioned above, Guo, Xie and co-workers strategically constructed an NIR colorimetric and fluorometric probe **1(Cu^2+^)**, featuring a bis-spiro rhodamine B framework, in 2023 [84]. The probe alone showed negligible color and fluorescence. Upon interaction with Cu^2+^, irreversible spirolactam ring opening was induced, generating a dominant absorption band at 700 nm with intensity linearly dependent on Cu^2+^ concentration. Concurrently, two emission bands emerged at 580 nm (λ_ex_ = 520 nm) and 770 nm (λ_ex_ = 650 nm). The probe showed high sensitivity for Cu^2+^ with LODs of 0.57 μM (colorimetric method), 9 nM (fluorometric at 580 nm), and 28 nM (fluorometric at 770 nm). Mechanistic studies revealed that the Cu^2+^-coordinated spiro-ring opening was followed by hydrolysis and acylation reactions, generating a new compound with NIR absorption/emission characteristics. The practical utility of **1(Cu^2+^)** was demonstrated by the successful fluorescence imaging of Cu^2+^ in HepG2 cells and in the mitochondria of paulownia sprout root tip cells.

Later, Yuan’s group established a distinctive rhodamine–hemicyanine platform in which Cu^2+^ coordination opened the spirolactam ring and suppressed PET, thereby switching on both color and NIR fluorescence [85,86,87,88]. In 2024, the group combined a hemicyanine dye with a rhodamine framework to synthesize the probe **NRh-NP** [85]. Upon coordination with Cu^2+^ in a 1:1 stoichiometry, the probe produced a pronounced “turn-on” fluorescence response at 750 nm (λ_ex_ = 660 nm) with a yellow-to-green color transition. **NRh-NP** performed effectively at pH 6.5–8.0 and showed negligible cytotoxicity (cell viability >98%), enabling imaging of exogenous/endogenous Cu^2+^ in MCF-7 cells and in living mice. Later that year, they further optimized the structure to obtain **NRh6G-FA** [86]. It enabled highly selective recognition of Cu^2+^ with strong interference resistance, rapid response (<60 s) and an LOD of 0.225 μM. Beyond cellular imaging, **NRh6G-FA** was successfully used for spiked recovery experiments and naked-eye detection of Cu^2+^ in Chinese herbal medicines (Astragalus and Paeonia lactiflora). Subsequently, **NRK-BD** was constructed on an extended π-conjugated xanthene–quinoline core using 2-pyridinecarboxaldehyde as the recognition unit. Coordination through the carbonyl O, imine N, and pyridine N atoms produced a strong emission at 730 nm, an LOD of 81 nM, and an ultrafast response of 1.5 s, while retaining EDTA-reversible behavior. **NRK-BD** also enabled test strip detection of Cu^2+^ on vegetable surfaces and smartphone-assisted quantitative analysis in real water samples with B/G values correlating linearly with Cu^2+^ (0–60 μM, LOD = 1.55 μM). With negligible cytotoxicity (cell viability > 96%), **NRK-BD** also achieved quantitative detection of intracellular Cu^2+^ in 4T1 cells, and fluorescence imaging of Cu^2+^ in living mice, with no significant attenuation of the fluorescence signal over time.

More recently, the same group obtained the probe **NRTB** by introducing ethyl phenothiazine group as a strong electron donor into the rhodamine framework. This modification effectively disrupted the electronic symmetry and bathochromically shifted the emission to 765 nm (λ_ex_ = 610 nm) [88]. Cu^2+^ recognition triggered a 268-fold fluorescence enhancement and a new absorption band at 600 nm, accompanied by a colorless-to-blue transition. **NRTB** demonstrated high selectivity towards Cu^2+^, with LODs of 22.6 nM by fluorescence and 43.28 nM by UV-Vis absorption. **NRTB** was successfully applied to smartphone-assisted visual quantification (LOD = 1.52 μM) and test strip-based rapid screening of Cu^2+^ residues on the surfaces of fresh produce. **NRTB** also demonstrated negligible cytotoxicity, with cell viability greater than 90%, enabling the effective imaging of Cu^2+^ in 4T1 cells as well as in mice, with an 80-fold signal enhancement in vivo.

Among these four probes, **NRh-NP**, **NRh6G-FA**, and **NRK-BD** are reversible and applicable over a certain pH range, and they could rapidly respond to Cu^2+^ in aqueous media (<60 s). In contrast, the original report on **NRTB** did not explicitly address its reversibility or pH tolerance. **NRTB** was used for Cu^2+^ detection in organic solvents and exhibited slower response kinetics, with a response time of 300 s. This slower response may be associated with its extended π-conjugated system and strong electron-donating phenothiazine moiety. Despite this, **NRTB** exhibited higher sensitivity. Collectively, these four probes illustrate a clear evolution from reversible, fast-responsive rhodamine–hemicyanine probes to a π-extended phenothiazine–rhodamine analogue with red-shifted emission and improved sensitivity, while also revealing a trade-off between extended conjugation and response kinetics.

Given the complexity of the pathological mechanisms underlying AD, the design of bifunctional probes capable of sequentially monitoring multiple related biomarkers is crucial for improving diagnostic accuracy. In 2025, Yan and colleagues rationally designed a panel of NIR fluorescent probes with a rhodamine spirolactam structure for sequentially detecting Cu^2+^ and Aβ42 aggregates [89]. **LDMD-N** exhibited an ultra-low LOD of 0.0543 μM for Cu^2+^ with rapid response (5 min), significantly outperforming the other candidates in the LDM series. Upon reaction with Cu^2+^, the ring opened to generate the compound **LDMD**, which could subsequently bind to Aβ42 aggregates, inducing an enhanced emission at 750 nm. **LDMD-N** was effectively used to monitor Cu^2+^ fluctuations in living cells and revealed that Cu^2+^ promoted Aβ aggregation in a zebrafish model. In vitro brain section staining and in vivo imaging experiments confirmed that **LDMD-N** could clearly distinguish AD model mice from wild-type controls, providing a powerful and versatile tool for elucidating the complex interplay between Cu^2+^ and Aβ aggregation in the pathogenesis of AD with potential applications in early diagnosis and therapeutic development.

#### 3.1.3. NIR Fluorescent Probes Derived from Hemicyanine/Cyanine Derivatives

**Figure 8:**
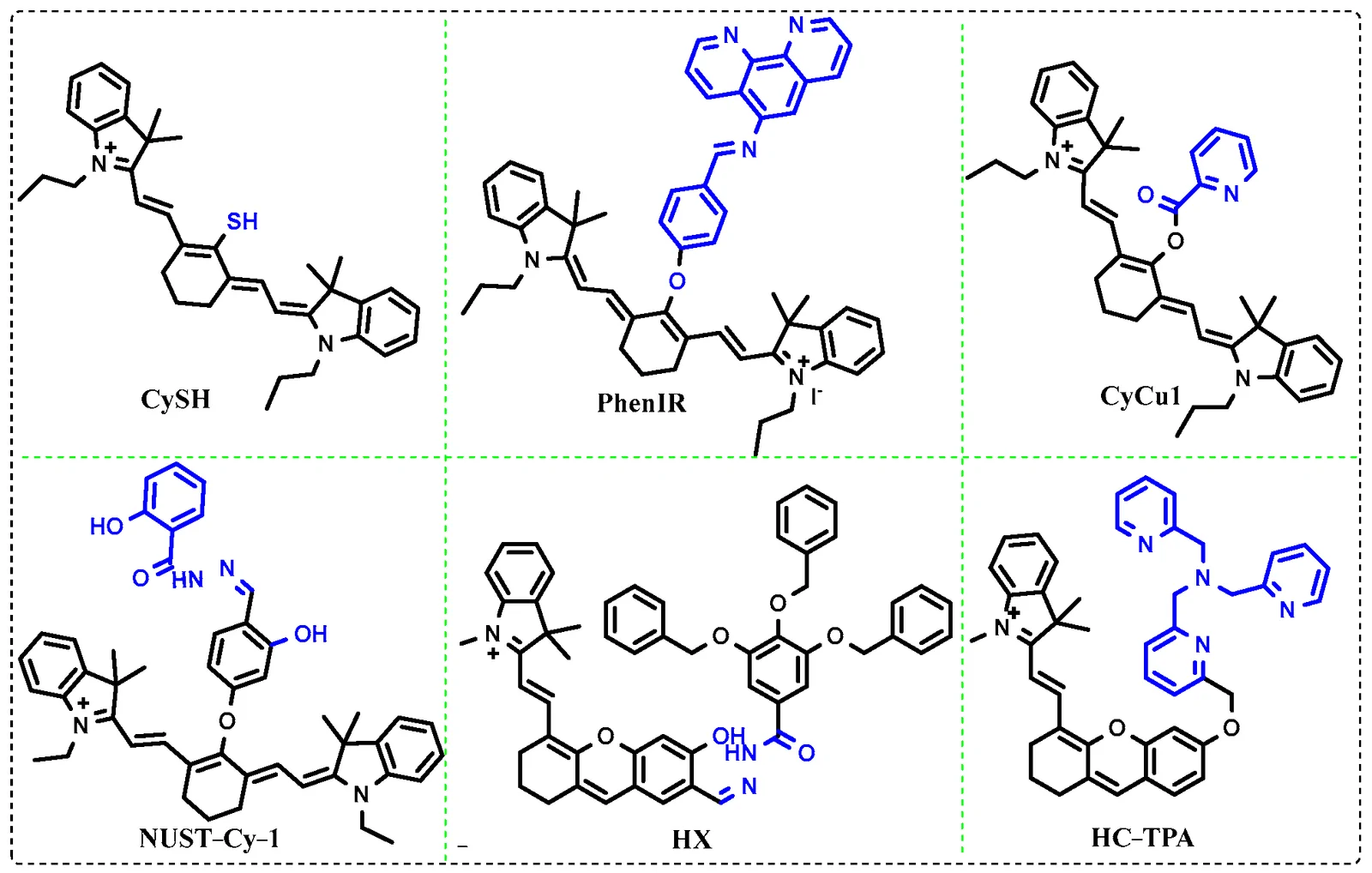
Structures of NIR fluorescent probes for Cu^2+^ based on rhodamine skeleton.

Hemicyanine/cyanine dyes have emerged as outstanding scaffolds for the development of NIR fluorescent probes for Cu^2+^ due to their excellent near-infrared optical properties, including high molar extinction coefficients, high fluorescence quantum yields, emission tunability with 650–900 nm, and good biocompatibility. These probes primarily respond to Cu^2+^ through two mechanisms: (1) fluorescence quenching caused by the paramagnetic nature of Cu^2+^ (off–on response); and (2) Cu^2+^-triggered chemical transformations (e.g., hydrolysis) that alter the probe structure, yielding pronounced spectral shifts suitable for ratiometric detection. More recently, researchers have reported various high-performance Cu^2+^ probes (Figure 8) by introducing various recognition groups into the hemicyanine/cyanine framework, demonstrating broad application prospects in subcellular structure imaging, in vivo imaging, and disease model studies [90,91,92,93,94,95].

On the common NIR fluorophore platform of cyanine, replacing the Cu^2+^ recognition unit allows for systematic modulation of the probe’s response mechanism and imaging performance. For example, in 2022, Wang’s group introduced a sulfhydryl group (-SH) into a cyanine scaffold and exploited sulfur donor-triggered PET to construct a turn-on NIR probe, **CySH** (λ_ex/em_ = 694/784 nm) [90]. Coordination of Cu^2+^ with –SH suppressed PET, causing a significant concentration-dependent increase in fluorescence. The 2:1 binding mode was identified by Job’s plot and HR-MS. **CySH** demonstrated favorable cell permeability, low cytotoxicity and mitochondria-targeting ability, enabling the fluctuation of labile copper in the mitochondria of HepG-2 cells to be monitored. Under copper-overload conditions, HepG-2 cells pretreated with NaVO_3_ and then exposed to CuCl_2_ exhibited a higher fluorescence intensity than cells exposed to CuCl_2_ alone, indicating that **CySH** enables monitoring of mitochondrial copper accumulation. Subsequently, Guo’s team reported a probe **IRPhen**, constructed by incorporating the classic Cu^2+^ ligand 1,10-phenanthroline into a cyanine framework via a Schiff base linker [91]. **IRPhen** absorbed strongly at 771 nm and emitted at 808 nm (λ_ex_ = 750 nm). Coordination with Cu^2+^ triggered rapid fluorescence quenching, driven by the paramagnetic nature of Cu^2+^ and the PET effect. **IRPhen** displayed an LOD of 0.286 μM, a 1:1 complexation stoichiometry with a high affinity (Kₐ = 1.3 × 10^6^ M^−1^), excellent selectivity, and good stability at physiological pH. Importantly, the probe was cell-permeable and enabled confocal imaging of dynamic Cu^2+^ in WS1 fibroblasts. These studies demonstrated that coordination-based recognition could achieve either turn-on or reversible turn-off responses, although turn-off modes may still suffer from background fluorescence interference.

**Figure 9:**
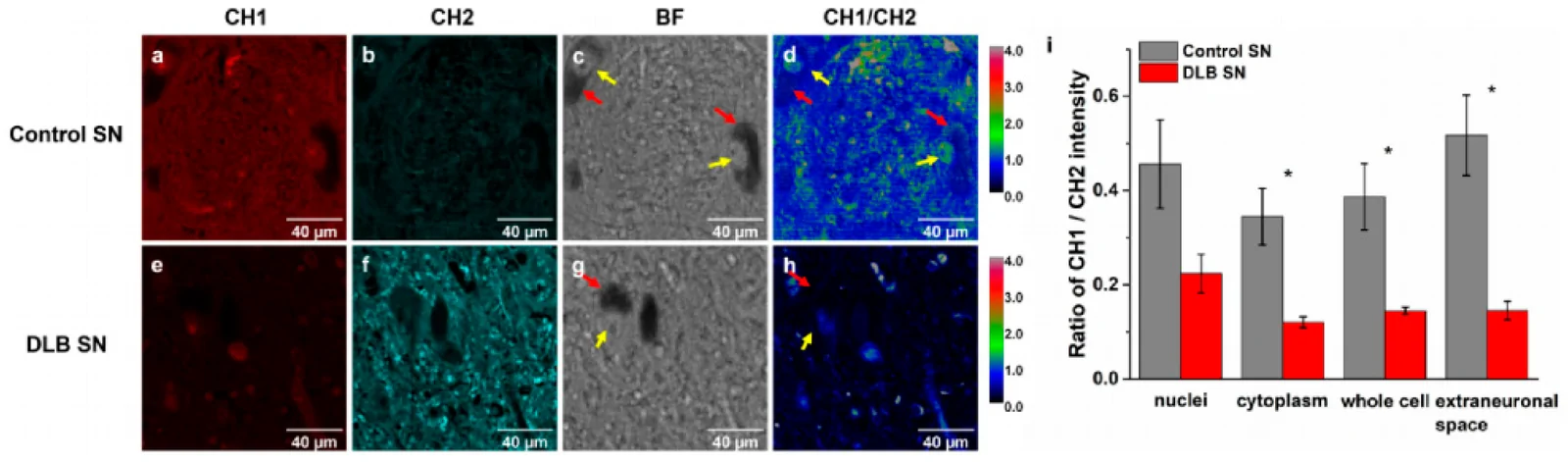
Representative fluorescence images of **CyCu1** for Cu^2+^ in the SN of human brain tissues from DLB patients and the control group. (**a**–**d**) Representative images of control SN. (**e**–**h**) Representative images of DLB SN. (**i**) Ratio of quantified fluorescence intensity in CH1 (580–650 nm) and CH2(750–800 nm) in nuclei, cytoplasm, whole cell, an extraneuronal space of control SN and DLB SN. The error bar represents SEM. * *p* < 0.05. Yellow arrow: nucleus; red arrow: cytoplasm. Reproduced with permission from Ref. [93]. Copyright (2024) American Chemical Society.

To circumvent paramagnetic quenching by Cu^2+^ and improve quantification, subsequent studies shifted toward reaction-based recognition and ratiometric signal output. New et al. [92] exploited the Cu^2+^-catalyzed hydrolysis of 2-picolinate esters to construct an NIR ratiometric probe, **CyCu1**, by modifying a cyanine scaffold (derived from IR-780). Upon reaction with Cu^2+^, **CyCu1** underwent hydrolysis followed by tautomerization to afford the ketone product **CyK1**, causing a dramatic hypsochromic shift exceeding 200 nm in both absorption and fluorescence emission, enabling ratiometric detection (I_600_/I_810_) with high sensitivity (LOD = 53 nM). **CyCu1** not only imaged exogenous Cu^2+^ in SH-SY5Y cells and neuroblastoma tumor tissues, but also effectively distinguished chelator-treated tumor tissues from controls, crucially offering the first detection of endogenous labile Cu^2+^ in neuroblastoma tumors. Most importantly, **CyCu1**, for the first time, revealed a significant decrease in Cu^2+^ levels in the cytoplasm, neurons, and extraneuronal space of the substantia nigra in patients with dementia with Lewy bodies (DLB) (Figure 9). By attaching a Schiff base structure bearing phenolic hydroxyl and C=N groups to the same cyanine fluorophore as that in **CyCu1** via an ester bond, a FRET-based fluorescent probe, **NUST-Cy-1**, was crafted [93]. Upon excitation at 450 nm, **NUST-Cy-1** displayed dual emission peaks at 593 nm and 825 nm, corresponding to the donor and the acceptor, respectively. However, Cu^2+^ induced the hydrolytic cleavage of the Schiff base unit and interrupted the FRET process, decreasing the 825 nm signal and generating a new peak at 790 nm, allowing for ratiometric detection (I_825_/I_790_) with an LOD of 1.4 μM. The bioimaging results demonstrated the successful application of **NUST-Cy-1** for ratiometric Cu^2+^ imaging in HeLa cells and living mice.

Overall, these four probes share the same cyanine fluorophore but employ different recognition units (SH, 1,10-phenanthroline, 2-picolinate ester, and Schiff base) to achieve PET turn-on, reversible turn-off, reaction-based ratiometric detection, and FRET ratiometric detection, respectively, extending Cu^2+^ imaging from the cellular level to mitochondria, tumor tissues, brain tissues, and living animals.

During the same period, exploiting the synthetic versatility of hemicyanine scaffolds, researchers conjugated hemicyanine to xanthene dyes via a C=C bridge to construct NIR fluorophores. The subsequent introduction of different Cu^2+^-specific recognition units afforded a series of high-performance NIR fluorescent probes. For instance, in 2025, a Schiff base moiety was introduced into a hemicyanine–xanthene scaffold as the Cu^2+^ recognition unit, yielding the Cu^2+^-responsive NIR fluorescent probe **HX** [94]. Among all the tested metal ions, only Cu^2+^ induced over 90% fluorescence quenching at 810 nm (λ_ex_ = 730 nm) within 60 s. **HX** showed a linear concentration-dependent fluorescence decrease upon Cu^2+^ binding, achieving an LOD of 0.54 μM. Taking advantage of its superior optical performance, **HX** was successfully used to visualize Cu^2+^ in H22 tumor-bearing mice. The results indicated that **HX** not only enabled visualization of Cu^2+^ accumulation in the liver for hepatotoxicity assessment but also allowed for the monitoring of tumor Cu^2+^ uptake to evaluate copper transporter (Ctr) function, offering a novel potential strategy for predicting tumor sensitivity to platinum-based drugs.

Recently, Li and colleagues incorporated a tris(2-pyridylmethyl)amine (TPA) unit into a hemicyanine–xanthene fluorophore analogous to that of **HX** and designed a reaction-based NIR fluorescent probe **HC-TPA**, achieving the cascade sensing of Cu^2+^ and GSH in complex food and biological systems, [95]. Upon rapid coordination with Cu^2+^, the fluorescence of **HC-TPA** at 668 nm was sharply quenched within 1 min. Subsequently, GSH triggered Cu^2+^-mediated oxidative cleavage, releasing the strongly NIR-emitting fluorophore **HC**, which restored the fluorescence at 696 nm within a response time of 20 min. DFT calculations revealed that **HC-TPA** exhibited weak intrinsic fluorescence due to the presence of a “dark” S_1_ state; Cu^2+^ coordination further suppressed the fluorescence via a CHEQ mechanism, while GSH achieved a fluorescence “turn-on” response through reductive oxidative cleavage. The excellent analytical performance, including linear detection ranges of 100–800 nM for Cu^2+^ (LOD = 48 nM) and 6–30 μM for GSH (LOD = 0.56 μM), rapid response kinetics, and exceptional selectivity over competing metal ions, biogenic amines, and other thiol-containing biomolecules such as Cys and Hcy, enables spiked recovery tests of Cu^2+^ and GSH in food matrices (e.g., apples, grapes, and tomatoes), as well as fluorescence imaging of Cu^2+^ and GSH in HEK293T cells. Herein, the probe offers a promising platform for food safety monitoring and bioanalytical applications involving interconnected metal–thiol systems, leveraged by a mechanistically driven tandem sensing strategy.

#### 3.1.4. NIR Fluorescent Probes Derived from Other NIR Fluorophores

**Figure 10:**
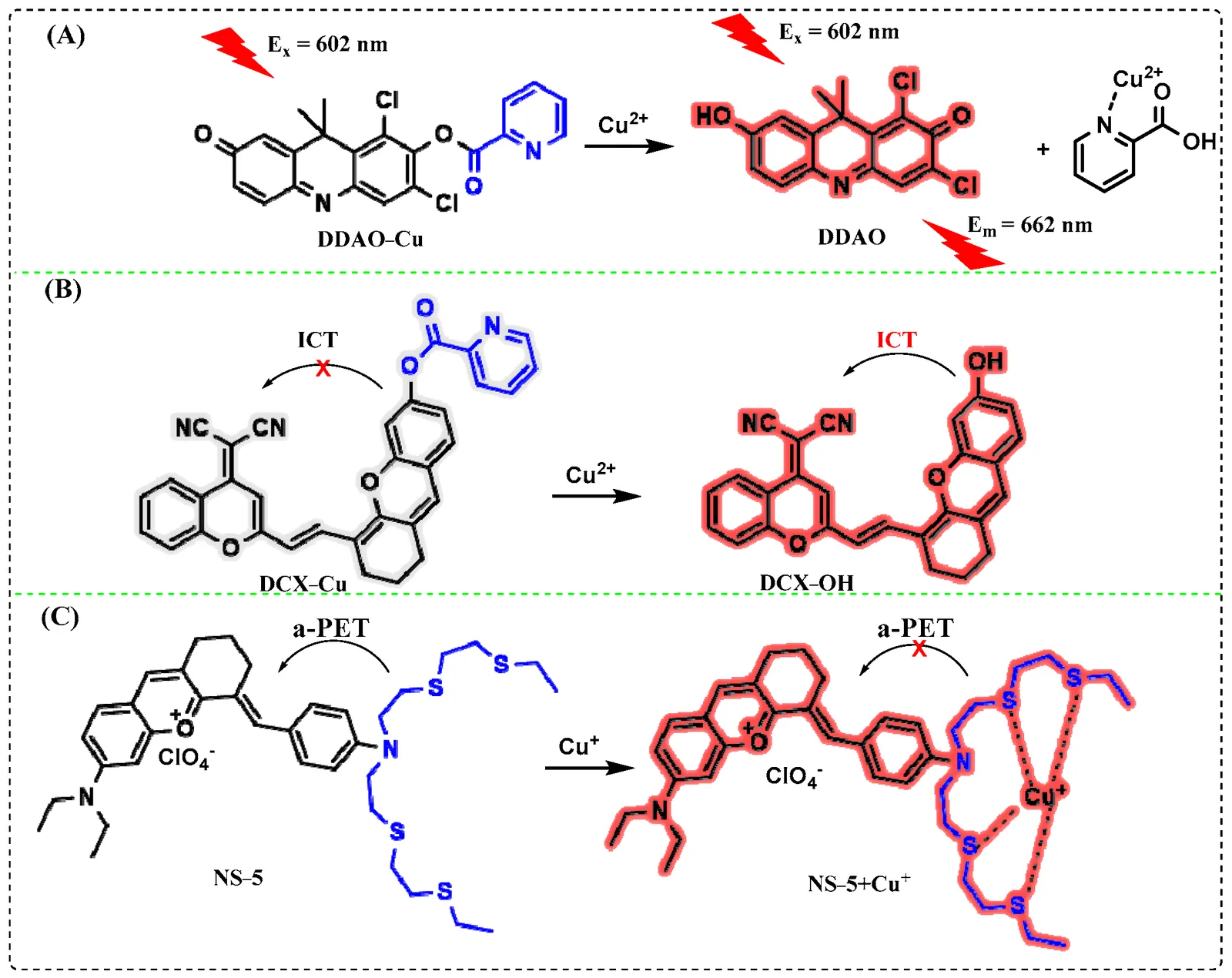
Mechanisms of Cu^2+^ sensing by **DDAO-Cu** (**A**), **DCX-Cu** (**B**) and **NS-5** (**C**).

In addition to the aforementioned common near-infrared (NIR) fluorophores, researchers have also explored other types of NIR dyes for the construction of fluorescent probes for Cu^2+^, as shown in Figure 10 [96,97,98]. Chen et al. introduced a picolinate moiety as the reaction site for Cu^2+^ into the NIR fluorophore 3-Dichloro-7-hydroxy-9,9-dimethylacridin-2(9H)-one (DDAO) to develop DDAO-Cu [96]. **DDAO-Cu** showed weak fluorescence owing to the inhibition of the ICT effect by the 2-picolinyl group. However, upon Cu^2+^-triggered hydrolysis of the picolinate ester, the hydroxyl group was released and the ICT effect was restored, causing a turn-on fluorescence response at 672 nm. **DDAO-Cu** exhibited ultra-high sensitivity (LOD = 1.8 nM) and selectivity towards Cu^2+^ with rapid response (<3 min) and favorable tissue penetration. **DDAO-Cu** was successfully used to image Cu^2+^ in cellular and zebrafish models of Parkinson’s disease (PD). Notably, **DDAO-Cu** demonstrated good blood–brain barrier permeability, achieving for the first time in situ imaging and differential analysis of Cu^2+^ levels in different brain regions (cortex, hippocampus, striatum, and substantia nigra) of Parkinson’s disease mice, revealing the complexity of copper distribution in brain regions under disease conditions.

Based on an identical recognition mechanism, Zhang’s group constructed **DCX-Cu** by introducing a 2-pyridinecarbonyl group into a benzopyranonitrile–xanthene scaffold [97]. Upon the addition of Cu^2+^, a strong fluorescence turn-on signal at 746 nm was observed for **DCX-Cu**, displaying a linear correlation with Cu^2+^ concentration (0–50 mM). Owing to its superior anti-interference performance and outstanding sensitivity (LOD = 19.47 nM), **DCX-Cu** enabled reliable Cu^2+^ detection in real water samples, with recoveries of 98–103%, and imaging of exogenous Cu^2+^ in HepG2 cells.

In 2025, Fang’s group introduced **NS-5**, an NIR probe for Cu^+^, featuring a tetradentate thioether ligand [98]. The PET-induced weak fluorescence was restored upon 1:1 Cu^+^ coordination, enabling rapid (>13-fold) enhancement at 720 nm with an LOD of 32 nM. **NS-5** demonstrated good photostability, functioned effectively in aqueous media, and specifically localized to the mitochondria with strong colocalization with Mito-Tracker Green (PCC = 0.90). Cell imaging studies confirmed that the probe could monitor both endogenous and exogenous Cu^+^ fluctuations in HepG2 cells and differentiate Cu^+^ levels between hepatocellular carcinoma (HepG2) and normal liver (L02) cells. Moreover, **NS-5** was effectively applied to in vivo imaging of labile Cu^+^ pools in living mice, successfully visualizing elevated Cu^+^ levels upon Cu(NO_3_)_2_ supplementation and reduced signals upon treatment with the copper chelator ATN-224.

### 3.2. NIR Fluorescent Probes for Hg^2+^

Among the most hazardous heavy metal pollutants, mercury ions (Hg^2+^) originate predominantly from anthropogenic sources, including fossil fuel combustion, mining, and industrial discharge [99]. Hg^2+^ exhibits strong bioaccumulation and persistence, with methylmercury (CH_3_Hg^+^) capable of biomagnifying millions-fold through the food chain [100]. Owing to its strong affinity for thiol groups present in biomolecules, even exposure to trace amounts of Hg^2+^ are sufficient to cause irreversible damage to the neurological, renal, and immune systems [101]. Consequently, the development of highly sensitive and selective methods for detecting Hg^2+^ in environmental and biological samples is of critical importance. Recently, various NIR fluorescent probes based on rhodamine, isophorone, DCM, heptamethine cyanine scaffolds, etc., have been developed to detect Hg^2+^. The photophysical properties of representative probes are summarized in Table 2.

#### 3.2.1. NIR Fluorescent Probes Derived from Dicyanoisophorone Derivatives

**Figure 11:**
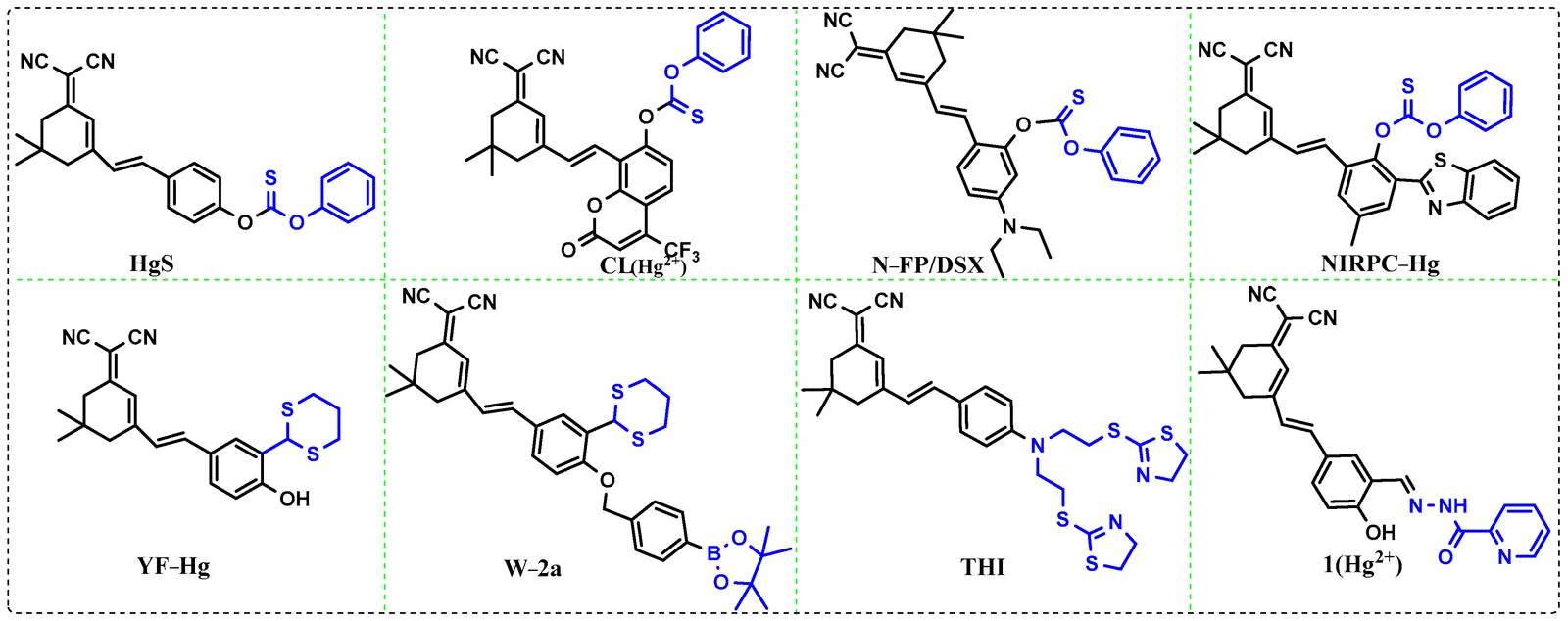
Structures of NIR fluorescent probes for Hg^2+^ based on dicyanoisohporone platform.

Leveraging the specific chemical reactivity of Hg^2+^, notably its ability to promote thioacetal/thioketal desulfurization and thiocarbonate hydrolysis, as well as principles of hard and soft acid–base (HSAB) theory, researchers have introduced various specific recognition groups (e.g., thioacetals/thioketals, thiocarbonates, and N/O/S-containing ligands) into the readily modifiable isophorone skeleton, which possesses desirable NIR luminescent properties. As illustrated in Figure 11, this strategy has enabled the creation of numerous superior fluorescent probes for Hg^2+^ detection [102,103,104,105,106,107,108,109,110].

Exploiting the high thiophilicity of Hg^2+^ to induce thiocarbonate hydrolysis, thereby triggering a fluorescence “turn-on” response, is a classic design strategy for Hg^2+^ probes. In 2022, Zhang and Zhou’s group presented the probe **HgS** by introducing a thiocarbonate moiety as the specific Hg^2+^ recognition unit into a dicyanoisophorone scaffold [102]. **HgS** exhibited fluorescence due to the PET effect. Upon specific reaction with Hg^2+^, the thiobenzoate moiety was cleaved and the ICT process was restored, triggering a pronounced emission increase at 645 nm (l_ex_ = 580 nm). **HgS** exhibited excellent selectivity and anti-interference, high sensitivity (LOD = 64 nM) towards Hg^2+^, and good biocompatibility, enabling successfully fluorescence imaging of exogenous Hg^2+^ in HeLa cells and living mice. Furthermore, **HgS** was effectively applied to the quantitative determination of Hg^2+^ in real water samples with satisfactory recoveries (83.4–108.6%).

Later, using the same mechanism mentioned above, Jiang et al. obtained the NIR fluorescent probe **CL(Hg^2+^)** by covalently conjugating a coumarin fluorophore with a dicyanoisophorone unit via a C=C bond to construct the NIR emissive skeleton, while introducing a thiocarbonate moiety as the responsive site for Hg^2+^/Ag^+^ [103]. Under excitation at 379 nm, **CL(Hg^2+^)** was weakly emissive at 695 nm. Upon addition of Hg^2+^ or Ag^+^, a strongly emissive coumarin–dicyanoisophorone fluorophore was released, which simultaneously triggered a remarkable emission enhancement and a visible color transition. **CL(Hg^2+^)** offers an exceptionally large Stokes shift (316 nm), outstanding selectivity, and good sensitivity, with LODs of 0.83 μM for Hg^2+^ and 8.8 μM for Ag^+^. Moreover, **CL(Hg^2+^)** features a rapid response to Hg^2+^ (<3 min), a wide pH tolerance (pH 2–12), and favorable biocompatibility (cell viability >90% even at 80 μM), enabling its successful application for in vitro fluorescence imaging of Hg^2+^ in U87MG cells, where the fluorescence intensity exhibits a concentration-dependent relationship with Hg^2+^.

Following a similar design strategy, in 2024, Li et al. constructed the probe **N-FP** [104]. Upon addition of Hg^2+^/Ag^+^ to **N-FP** solution, a distinct a fluorescence turn-on at 661 nm was observed, along with a bathochromic shift and a color change. **N-FP** exhibited LODs of 0.72 μM for Hg^2+^ and 1.1 μM for Ag^+^, rapid response (1–3 min), excellent selectivity, and efficient performance across an exceptionally wide pH range of 1–10. These features enabled its successful application for colorimetric and fluorimetric detection of Ag^+^ and Hg^2+^. **N-FP** was also investigated in parallel by another group [105]. Interestingly, in this report, the probe was developed for Hg^2+^-selective detection, featuring strong anti-interference performance, and an ultra-low LOD of 6.57 nM, enabling successful application in water and food sample analysis with satisfactory recoveries (93.79–101.80%). Benefiting from its low cytotoxicity and good photostability, the probe was further extended to visualize Hg^2+^ in HeLa cells and zebrafish via fluorescence imaging. After incubation with Hg^2+^, the intracellular red fluorescence was remarkably attenuated.

Based on a synergistic ICT-ESIPT mechanism, in 2025, Zhu and co-workers constructed **NIRPC-Hg** by integrating a benzothiazole ESIPT unit, a dicyanoisophorone ICT unit, and a thiocarbonate moiety as the Hg^2+^-specific recognition group for ratiometric fluorescence detection of Hg^2+^ [106]. The probe was initially emitted at 535 nm. However, upon Hg^2+^-promoted hydrolysis, the phenol was released, restoring the ICT-ESIPT pathway and shifting the emission to 665 nm, enabling ratiometric detection with an ultra-low LOD (9.21 nM). Using **NIRPC-Hg**, Hg^2+^ was successfully detected in environmental water samples and exogenous Hg^2+^ was imaged ratiometrically in HepG2 cells and zebrafish.

Beyond Hg^2+^-induced thiocarbonate hydrolysis, thioacetal deprotection is another key strategy for probe design. Guided by this mechanism, Chen’s group (2023) constructed a dual-channel colorimetric and NIR fluorescent probe **YF-Hg** by introducing a thioacetal moiety (a specific reaction site for Hg^2+^) into the malononitrile isophorone backbone [107]. After the addition of Hg^2+^, **YF-Hg** underwent a desulfurization reaction to generate the aldehyde precursor YF-CHO, causing a 12-fold fluorescence turn-on at 659 nm. Meanwhile, the probe exhibited a blue shift in absorption along with a distinct violet-to-red color variation. Selectivity experiments demonstrated that, apart from Hg^2+^, other common ions, bioactive molecules, H_2_S, and H_2_O_2_ induced no significant spectral changes in **YF-Hg**. Moreover, their coexistence with Hg^2+^ did not interfere with Hg^2+^ detection by **YF-Hg**. In addition, **YF-Hg** exhibited a large Stokes shift (191 nm), favorable membrane permeability, and low cytotoxicity (mouse fibroblast viability >80% at 100 μM), enabling successful fluorescence imaging of Hg^2+^ in mouse fibroblasts, where intense red fluorescence signals were observed in the group treated with Hg^2+^. Furthermore, dual-colorimetric test strips were developed based on its color response, allowing for naked-eye, semi-quantitative visual detection of Hg^2+^.

Han and colleagues (2023) rationally designed **W-2a** by incorporating a phenylboronic acid pinacol ester as an ONOO^−^ recognition unit into a dithioacetal-based Hg^2+^ probe for the sequential and discriminative detection of Hg^2+^ and peroxynitrite (ONOO^−^) [108]. **W-2a** exhibited weak fluorescence due to hindered ICT. Hg^2+^ triggered dithioacetal deprotection, producing fluorescence at 653 nm, while subsequent ONOO^−^ cleavage of the phenylboronate ester further intensified the emission at 656 nm, facilitating stepwise detection at 656 nm of Hg^2+^ and ONOO^−^. The detection limits were 1.46 μM for Hg^2+^ and 97 nM for ONOO^−^ (Hg^2+^ first) or 7.25 nM and 0.12 μM (ONOO^−^ first). **W-2a** effectively enabled multi-channel fluorescence imaging of EMT6 cells, allowing us to monitor intracellular Hg^2+^ and ONOO^−^ levels. Furthermore, **W-2a** was utilized for imaging endogenous ONOO^−^ in an LPS-induced mouse model of inflammation. Compared with normal tissue in the right leg, the inflammatory region in the left leg exhibited significantly enhanced fluorescence signals. Coupled with a smartphone-based portable device and a WeChat mini-program (“Colorimetric acquisition”), the probe exhibited a good linear relationship between the R/B ratio and Hg^2+^ concentration (R^2^ > 0.9) in both fluorescence and UV–vis dual modes, thereby providing an intelligent, on-site analytical platform for rapid qualitative and quantitative analysis of Hg^2+^ in the environment. Overall, **W-2a** serves as a versatile tool for studying Hg^2+^-induced oxidative stress and its downstream peroxynitrite signaling in living systems.

Apart from utilizing specific chemical reactions, Hg^2+^ fluorescent probes can also be developed through the coordination of Hg^2+^ with nitrogen, oxygen, and sulfur atoms, as guided by Pearson’s HSAB theory. Based on this approach, in 2022, Erdemir et al. synthesized an NIR probe **THI** derived from a thiazoline–isophorone framework [109]. A significant fluorescence turn-on response at 645 nm was elicited upon Hg^2+^ coordination with the thiazoline N/S atoms, which disrupted hydrogen bonding between water molecules and the diethylamino group, restored the ICT pathway, and resulted in an ~11-fold increase in quantum yield (from 0.0106 to 0.1177), a slight blue shift, and an LOD of 7.22 μM. The recognition process was reversible, as demonstrated by S^2−^-induced fluorescence quenching. Its practical utility has been validated through portable TLC test strip assays for naked-eye Hg^2+^ detection and successful application in real water sample analysis. Additionally, **THI** showed good permeability and low cytotoxicity. Cytotoxicity assays showed no obvious toxic effects on either HeLa or Hep2 cells, and the probe was applied to fluorescence imaging of Hg^2+^ in living HeLa cells, where bright red fluorescence signals were observed.

In 2024, Yan’s group constructed the fluorescent probe **1(Hg^2+^)** via the condensation reaction of dicyanoisophorone aldehyde with 2-pyridinecarbohydrazide [110]. Upon coordination with Hg^2+^ in a 1:1 stoichiometry, the PET process was inhibited, generating a turn-on NIR signal at 660 nm together with a linear detection range of 0–7 μM. Mechanistic studies revealed that coordination occurred exclusively through the pyridine nitrogen, while the hydroxyl and amide groups were not involved. Within the physiological pH range (7.5–9.5), Probe **1** offered remarkable selectivity (Ka = 1.63 × 104 M^−1^), excellent sensitivity (LOD = 5.6 nM), a 220 nm Stokes shift and a rapid equilibration time (30 s). The successful imaging of Hg^2+^ in HepG2 cells and reliable recovery tests in real water samples further confirmed its practical applicability.

#### 3.2.2. NIR Fluorescent Probes Derived from Rhodamine Derivatives

**Figure 12:**
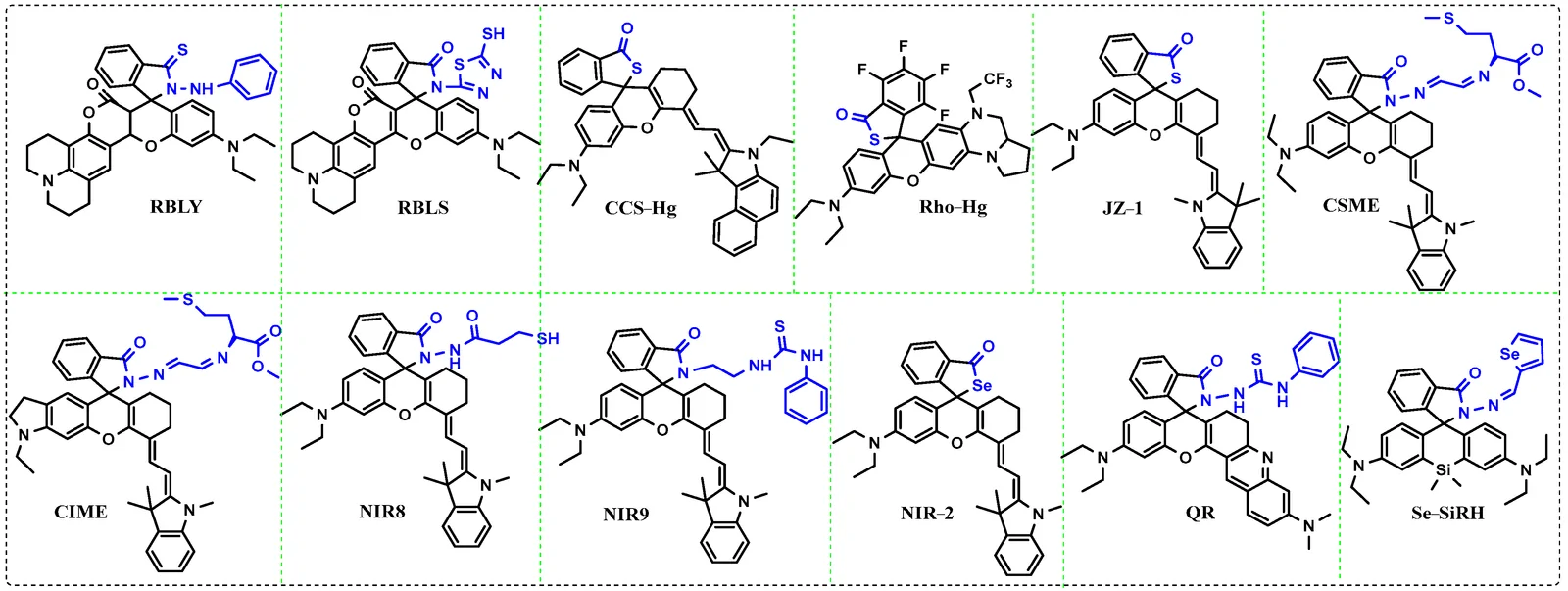
Structures of NIR fluorescent probes for Hg^2+^ based on rhodamine derivatives.

Rhodamine dyes, characterized by their unique spirocyclic lactam structure, high fluorescence quantum yields, and excellent photostability, have emerged as an ideal platform for constructing “turn-on” fluorescent probes. In recent years, researchers have successfully extended the emission wavelengths of rhodamine derivatives into the NIR region by expanding the π-conjugation system or introducing heterocyclic structures, thereby significantly enhancing their application potential in bioimaging [111,112,113,114,115,116,117,118,119,120,121,122], as shown in Figure 12.

In 2022, utilizing a coumarin–rhodamine fused dye as the fluorophore, Kan’s group reported a novel fluorescent probe **RBLY**, via acyl chlorination, phenylhydrazine coupling, and Lawesson’s reagent sulfurization [111]. The probe exhibited negligible emission due to the dominant spirolactam conformation. Upon exposure to Hg^2+^, the spirocycle was selectively cleaved, triggering a pronounced turn-on response at 695 nm, with a detection limit of 0.34 μM. The 1:1 binding mode was confirmed by Job’s plot analysis and mass spectrometry and the binding constant was determined to be 1.63 × 10^5^ M^−1^. The recognition of Hg^2+^ by **RBLY** was reversible (quenchable by S^2−^), and the system reached equilibrium within approximately 120 s. **RBLY** was capable of detecting Hg^2+^ in the actual environmental water samples. More importantly, the successful visualization of Hg^2+^ in MCF-7 cells, soybean root tissue, and zebrafish was achieved by using **RBLY**, underscoring its wide applicability in both plant and animal systems.

Subsequently, Kan’s group reported another structurally similar NIR fluorescent probe **RBLS** [112]. Complexation with Hg^2+^ in a 2:1 stoichiometric ratio triggered a prominent emission turn-on at 685 nm, yielding an LOD of 0.26 μM. Similar to **RBLY**, the recognition of Hg^2+^ by **RBLS** was reversible, and the probe proved effective for quantifying trace Hg^2+^ in environmental water samples and for fluorescence imaging in biological models. Notably, leveraging the distinct colorimetric response of **RBLS**, the research team further developed a test strip for naked-eye visualization of Hg^2+^, which exhibited a color change from slight yellow to blue as the Hg^2+^ concentration increased. More importantly, by integrating the test strip with smartphone photography and RGB color analysis, quantitative detection of Hg^2+^ was achieved with an LOD of 1.87 μM, thereby offering a practical tool for rapid on-site monitoring of Hg^2+^.

Liang et al. introduced a sulfur atom into chromenylium-cyanine dye via sodium sulfide treatment to yield **CCS-Hg**, which retained the classical “off–on” response characteristics of traditional rhodamines while offering the advantages of near-infrared excitation and emission (λ_ex/em_ = 680/780 nm) [113]. Upon coordination with Hg^2+^ in a 2:1 stoichiometric ratio, **CCS-Hg** displayed a strong NIR emission at 780 nm, along with a marked increase in absorbance at 680 nm and 740 nm, thereby achieving both fluorometric and colorimetric detection. Spectroscopic investigations revealed that **CCS-Hg** exhibited excellent selectivity and high sensitivity for Hg^2+^ detection, achieving detection limits of 73 nM (fluorometric mode) and 12 nM (colorimetric mode), and quick response (<30 s), under conditions at pH 5–9. Benefiting from these advantages, **CCS-Hg** was successfully employed for fluorescence imaging of Hg^2+^ distribution in living A549 cells.

Similar to **CCS-Hg**, Yuan’s group successfully engineered Rho-Hg by introducing a thiolactone moiety as the Hg^2+^-responsive switch into the YLF dye platform [114]. **Rho-Hg** showed a dramatic 14-fold fluorescence surge at 665 nm upon Hg^2+^ addition, with a low LOD of 17.5 nM, a rapid equilibration time (<1 min), and excellent pH tolerance spanning from 2 to 10. Colocalization experiments revealed that the red fluorescence signal was predominantly localized within mitochondria and exhibited substantial overlap with the green fluorescence of Mito-Tracker Green (Pearson’s correlation coefficient = 0.8), indicating that **Rho-Hg** possessed mitochondrial targeting capability. Owing to these merits, **Rho-Hg** was successfully utilized not only for imaging mitochondrial Hg^2+^ in HeLa cells but also for dynamically tracking Hg^2+^ accumulation and migration during zebrafish development. Fluorescence imaging revealed that as zebrafish grew to maturity, the red fluorescence signal gradually translocated from the embryonic chorion to the liver and intestine of the larvae, indicating the progressive transfer of Hg^2+^ to these organs.

More recently, Zhou’s team presented a fluorescent probe **JZ-1**, structurally analogous to **CCS-Hg** [115]. The probe **JZ-1** featured dual NIR analytical wavelengths, with both excitation and emission maxima exceeding 700 nm (λ_ex_ = 700 nm, λ_em_ = 728 nm), which minimized background autofluorescence and enhances tissue penetration. Upon reaction with Hg^2+^, the solution of **JZ-1** displayed a pronounced turn-on fluorescence signal at 780 nm (>200-fold), accompanied by significantly enhanced absorption bands at 663 nm and 722 nm. **JZ-1** demonstrated high selectivity towards Hg^2+^, a low detection limit of 33 nM, and stable performance across a broad pH range (3.7–10.0) and temperature range (25–45 °C). Cell imaging studies showed that **JZ-1** selectively accumulated in the mitochondria and effectively visualized exogenous Hg^2+^ in HeLa cells, while zebrafish experiments further validated its capacity for monitoring Hg^2+^ accumulation in vivo. Notably, the probe was effectively applied to image gastrointestinal damage in an acute mercury poisoning mouse model, intuitively revealing Hg^2+^ accumulation in the gastrointestinal tract. Additionally, H&E staining confirmed the mitigating effect of sodium selenite on mercury-induced toxicity.

Moreover, based on the chromenylium-cyanine platform, Ismail Yilmaz’s group systematically constructed a family of structurally analogous NIR fluorescent probes for sensing Hg^2+^, with progressive optimization through different recognition units (methionine, mercaptopropanehydrazide, thiourea, selenolactone), achieving improved sensitivity, response speed, and versatility for environmental and biological applications [116,117,118,119,120]. In 2023, they reported **CSME**, a methionine-modified probe that responded to Hg^2+^ via ratiometric and turn-on modes [116]. Upon Hg^2+^ addition, **CSME** exhibited fluorescence enhancement at 740 nm (linear range: 9.49 × 10^−9^–1.13 × 10^−5^ M) and a ratiometric absorbance change (A_711_/A_385_) over 6.29 × 10^−8^–1.86 × 10^−4^ M, together with a distinct colorimetric change from pale yellow to green. The sensing system reached equilibrium in just 90 s, offering LOD values of 58.9 nM (UV-Vis) and 4.93 nM (fluorescence). The practical applicability of **CSME** was further demonstrated through ratiometric/turn-on assays in real water, smartphone-assisted colorimetric analysis, and NIR fluorescence imaging of 3T3 cells.

In 2024, they developed **CIME**, a structurally similar probe with faster response (30 s) and higher sensitivity (LOD = 0.65 nM), enabling dual-mode detection in water samples, smartphone analysis, and imaging in MCF-7 and 3T3 cells [117]. In the same year, they reported **NIR8**, featuring a mercaptopropanehydrazide recognition group, which exhibited the fastest response (15 s) and detection limits of 49.3 nM (UV-Vis) and 11.3 nM (fluorescence) [118]. **NIR8** was applied to water sample analysis, smartphone RGB detection, and imaging in MCF-7 and NIH/3T3 cells. In 2025, they introduced **NIR9**, a dual chemosensor capable of independently detecting Hg^2+^ (turn-on fluorescence at 750 nm, LOD = 20 nM) and Cu^2+^ (turn-off UV-Vis at 380 nm, LOD = 10 nM). The Hg^2+^ response involves desulfurization-induced spirolactam ring opening, while Cu^2+^ simply coordinates to the thiourea moiety without ring opening [119]. **NIR9** has been successfully applied in environmental water, food samples, and A549 cell imaging. Most recently (2026), they designed **NIR-2**, a selenolactone-based probe that displayed strong turn-on fluorescence at 755 nm (LOD = 4.54 nM) and a ratiometric UV-Vis response (LOD = 0.377 nM) to Hg^2+^ [120]. **NIR-2** featured an ultra-fast response (10 s), the widest linear range (1–10 nM), and the most sensitive smartphone-based detection (linear range 1.66 nM-7.50 μM). The researchers successfully employed **NIR-2** for Hg^2+^ detection in water, food, and legume samples, and also utilized it for fluorescence imaging in A549 and H1299 cancer cells. All probes operated via the Hg^2+^-induced spiro ring opening mechanism, offering NIR emission, high sensitivity, fast response, dual-functional detection, and cell imaging capabilities, making them effective tools for convenient Hg^2+^ detection in environmental and biological samples.

A quinoline–rhodamine-based ratiometric NIR probe **QR** was engineered by Wang’s team, featuring a phenylthio ester group for selective Hg^2+^ recognition [121]. **QR** itself emitted visible fluorescence at 465 nm. Only Hg^2+^ promoted a spirolactam ring opening reaction, which induced significant expansion of molecular conjugation and generated a 1,3,4-oxadiazole moiety. This structural transformation triggered a ratiometric response (I_725_/I_465_) with a remarkable 354-fold enhancement. The probe possessed exceptional selectivity, fast response (<1 min), a remarkable Stokes shift, and a low LOD of 23 nM for sensing Hg^2+^. Leveraging its excellent optical properties, **QR** was effectively utilized to monitor Hg^2+^ in tap water with satisfactory recoveries. Moreover, **QR** demonstrated negligible cytotoxicity (cell viability >90% at 10 mM) and excellent cell-penetrating ability, facilitating the dual-channel ratiometric fluorescence visualization of Hg^2+^ in live HeLa cells, where the red channel fluorescence signal increased greatly while the green channel signal decreased upon the addition of Hg^2+^.

Recently, Wang and co-workers pioneered the development of an NIR probe **Se-SiRH**, featuring a selenophene-functionalized silicon–rhodamine B scaffold, leveraging the excellent chelation ability of the selenium atom toward Hg^2+^ [122]. The probe itself was colorless and non-fluorescent. Upon coordination with Hg^2+^ in a 2:1 stoichiometric ratio, **Se-SiRH** underwent spirocyclic opening, producing a >75-fold fluorescence turn-on at 672 nm and a colorless-to-pink color change. The sensing mechanism, involving Hg^2+^-triggered spirolactone opening facilitated by coordination via the carbonyl oxygen and selenophene selenium atom, was further validated by DFT calculations, which revealed a reduced HOMO-LUMO energy gap upon complexation. Owing to its superior selectivity and ultra-high sensitivity (LOD = 2.1 nM), the applicability of **Se-SiRH** was further extended to Hg^2+^ detection in environmental water matrices and confocal imaging in HepG2 cells.

#### 3.2.3. NIR Fluorescent Probes Derived from Hemicyanine/Cyanine Dyes

**Figure 13:**
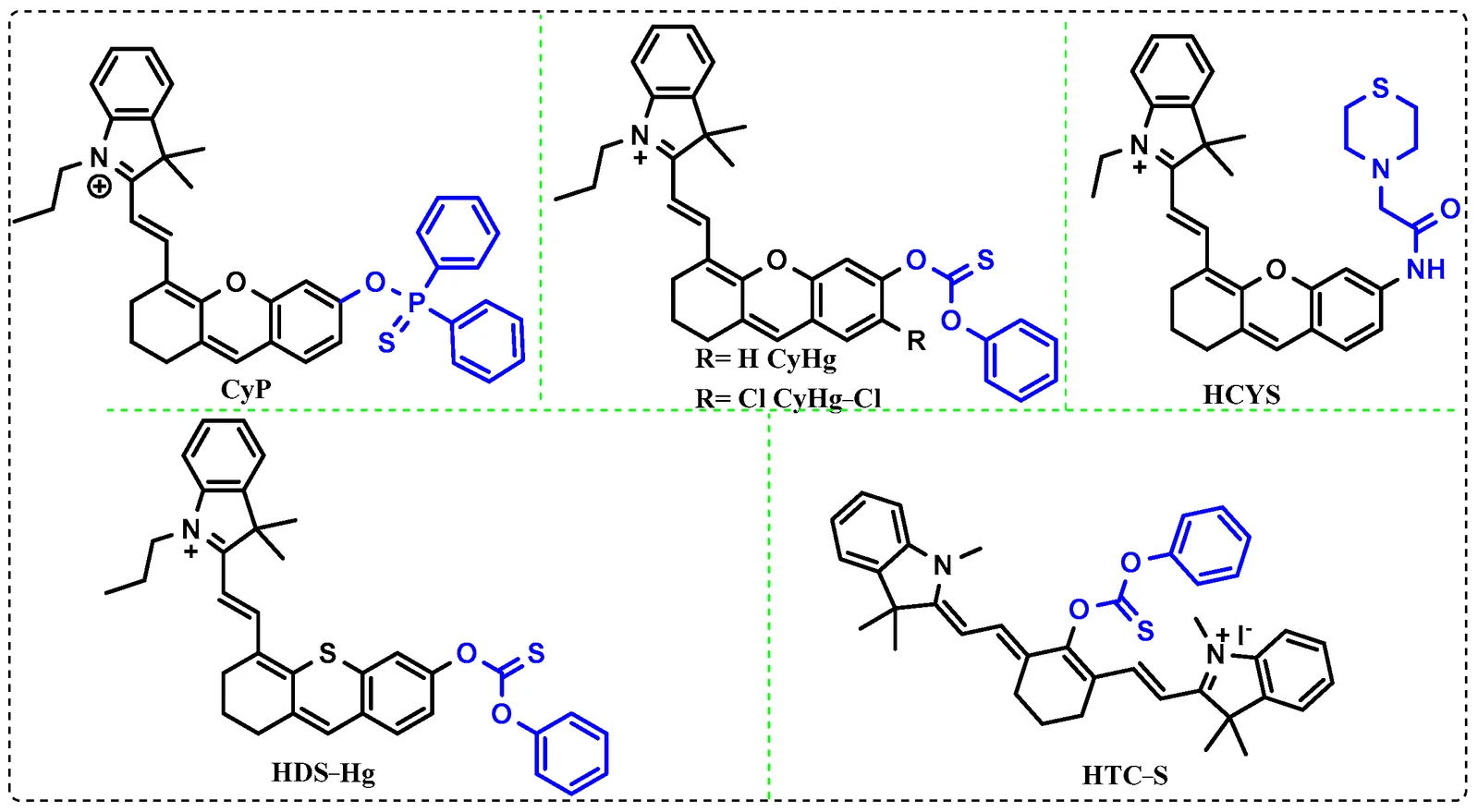
Structures of NIR fluorescent probes for Hg^2+^ based on hemicyanine/cyanine dyes.

NIR fluorescent probes constructed from a hemicyanine/cyanine scaffold have attracted considerable attention for detecting Hg^2+^ owing to superior photophysical properties. By exploiting the high thiophilicity of Hg^2+^, a series of novel NIR fluorescent probes (Figure 13) were developed by introducing specific recognition groups (such as carbonothioate, phosphinothioate, etc.) into hemicyanine or cyanine platforms to effectively modulate the ICT process, enabling turn-on, ratiometric, or quenching fluorescence responses toward Hg^2+^ [123,124,125,126,127].

**Figure 14:**
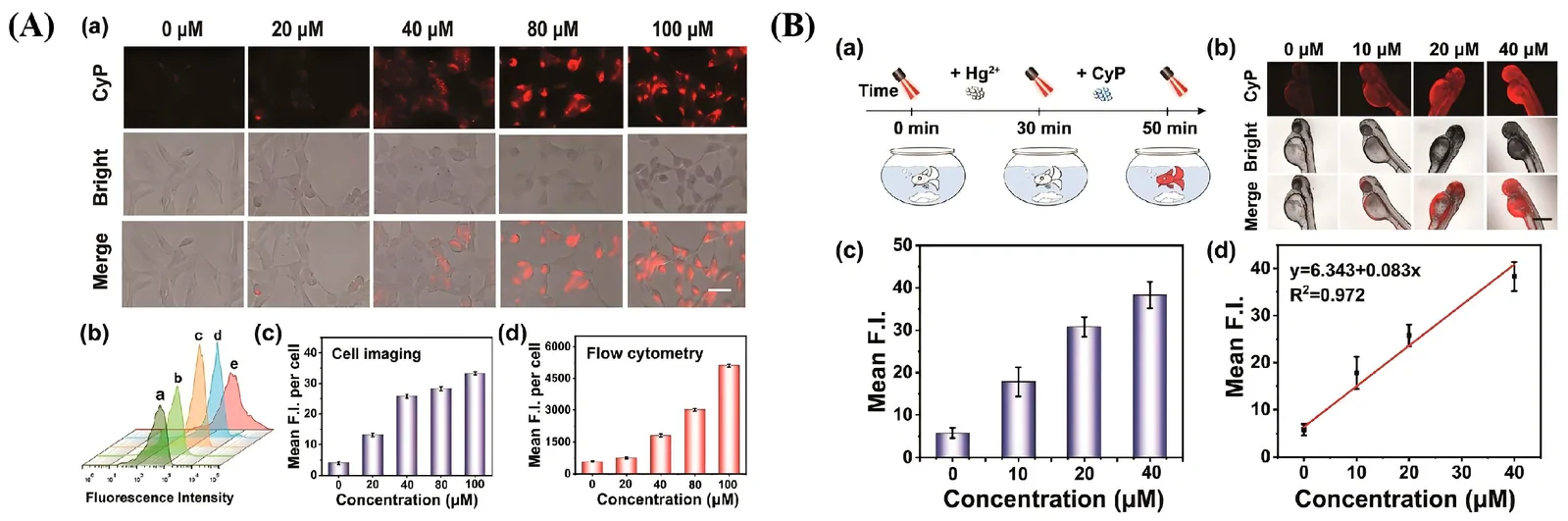
(**A**) (**a**) Fluorescence imaging of different concentrations of Hg^2+^ in SHSY-5Y cells; (**b**) The corresponding flow cytometric analysis. Letters a–e represent Hg^2+^ concentrations of 0, 20, 40, 80, and 100 μM, respectively; (**c**) The corresponding mean fluorescence intensities in cell imaging. (**d**) The corresponding mean fluorescence intensities of SHSY-5Y cells determined by flow cytometry; (**B**) Fluorescence imaging of Hg^2+^ in zebrafish. (**a**) Diagram of the zebrafish imaging experiment process; (**b**) Fluorescence imaging of different concentrations of Hg^2+^ in zebrafish; (**c**) The corresponding mean fluorescence intensities in zebrafish imaging; (**d**) Plot of the linear relationship between Hg^2+^ concentration and fluorescence intensity in zebrafish. Reproduced with permission from Ref. [123]. Copyright (2022) Royal Society of Chemistry.

In 2022, Zhang’s group developed an “off–on” NIR probe **CyP**, built on a hemicyanine skeleton, for selective detection and imaging of Hg^2+^ in cells and animals [123]. The probe was composed of a xanthene unit, a trimethylindoline moiety, and a diphenylphosphinothioic chloride group. The fluorescence was initially quenched via covalent modification of the phenolic hydroxyl group with a diphenylphosphinothioate moiety, which suppressed the ICT process. Upon reaction with Hg^2+^, **CyP** underwent hydrolysis to product the fluorophore CyOH, restoring the ICT pathway and producing a strong NIR emission at 710 nm. The probe exhibited remarkable selectivity for Hg^2+^, accompanied by a binding constant of (1.45 ± 0.12) × 10^5^ M^−1^, an LOD of 54.8 nM, and a rapid equilibration time (10 min). In addition, **CyP** worked well across a broad pH window (4.0–9.0) and showed low cytotoxicity, enabling successful fluorescence imaging of exogenous Hg^2+^ in SHSY-5Y neuroblastoma cells and zebrafish with good concentration-dependent signal enhancement (Figure 14). Furthermore, the probe demonstrated excellent tissue penetration (up to 800 μm) and was successfully applied for subcutaneous imaging in Balb/c mice, underscoring its potential as a versatile NIR tool for monitoring Hg^2+^-associated pathological conditions in complex biological environments.

Based on the similar mechanism mentioned above, Shang and Fang et al., in 2024, reported two hemicyanine-bassed probes **CyHg** and **CyHg-Cl**, employing a carbonothioate group as the Hg^2+^ recognition unit [124]. Notably, higher reactivity was observed for **CyHgCl** compared to **CyHg**, attributed to the electron-withdrawing effect of the chlorine substituent. The probe responded to Hg^2+^ via cleavage of the carbonothioate ester, releasing the chloro-substituted hemicyanine fluorophore that emitted strongly at 725 nm in the NIR region. Moreover, under identical experimental conditions, other common heavy metal ions such as Pb^2+^, Ag^+^, Cu^2+^, Cd^2+^, and Zn^2+^ caused no appreciable changes in the fluorescence spectrum of **Cy-Hg**, whereas Hg^2+^ did, demonstrating the good selectivity of **Cy-Hg** for Hg^2+^. **CyHg-Cl** was also capable of sensing Hg^2+^ in living HeLa cells and authentic water specimens. MTT assays demonstrated the low cytotoxicity of **CyHg-Cl** in HeLa cells (cell viability > 90% at 20 μM, IC_50_ = 72 μM), and confocal imaging experiments confirmed its excellent membrane permeability and ability to visualize intracellular Hg^2+^. Furthermore, **CyHg-Cl** was successfully utilized to detect Hg^2+^ in real water samples.

Subsequently, Liu and colleagues fabricated **HCYS**, an NIR probe based on a hemicyanine framework featuring a thiomorpholine moiety as the Hg^2+^ recognition unit, for Hg^2+^ detection in environmental and biological systems via the ICT mechanism [125]. Among the common metal ions tested, only Hg^2+^ induced a rapid decrease in the fluorescence intensity of **HCYS** at 674 nm within 1 min, owing to its spin–orbit coupling and heavy atom effects. Furthermore, the fluorescence intensity exhibited a good linear relationship with Hg^2+^ concentration over the range of 0–25 μM, with a detection limit of 1.41 μM. The probe enabled imaging of Hg^2+^ in various cell lines, such as SH-SY5Y, HepG2 cells and HeLa cells, where the intracellular red fluorescence gradually diminished with prolonged incubation with Hg^2+^ and was recovered upon the addition of EDTA. It also allowed for dynamic monitoring of Hg^2+^ in living zebrafish. Moreover, **HCYS** can dynamically monitor Hg^2+^ in living zebrafish. Importantly, **HCYS** was capable of imaging Hg^2+^ in both longitudinal and transverse root slices of pea sprouts, confirming its ability to track mercury contamination in plant tissues.

In 2025, Liu et al. designed and synthesized **HDS-Hg**, a thiahemicyanine-based probe structurally similar to **CyHg** but featuring a monothiocarbonate group [126]. The probe itself exhibited weak fluorescence due to the electron-withdrawing thiocarbonate group that suppressed ICT [126]. Upon selective cleavage of the carbon–sulfur bond triggered by Hg^2+^, the NIR fluorophore **HDS** was released, restoring ICT and producing a pronounced turn-on signal at 765 nm. **HDS-Hg** demonstrated the advantages of a rapid response, high sensitivity (LOD = 89 nM), wide pH tolerance, superior selectivity, and favorable mitochondrial localization ability (Pearson’s coefficient = 0.94). The probe enabled not only the quantitative detection of Hg^2+^ in environmental water samples, herbal medicines, and seafood, but also fluorescence imaging of Hg^2+^ in HeLa cells, notably in tobacco seedling leaves, which was a rarely explored application for Hg^2+^ probes. Furthermore, owing to its NIR excitation and emission properties, **HDS-Hg** enabled in situ imaging in a mouse model of chronic mercury poisoning. The imaging results revealed that the red fluorescence signal was predominantly localized in the liver and exhibited a time-dependent increase with prolonged mercury exposure, demonstrating the potential of **HDS-Hg** for real-time monitoring and early diagnosis of Hg^2+^-induced liver injury.

Erdemir et al. constructed **HTC-S** for ratiometric and colorimetric sensing of Hg^2+^, by integrating a carbonothioate recognition group into a cyanine scaffold [127]. Upon specific reaction with Hg^2+^, **HTC-S** underwent hydrolysis, disrupting its extended π-conjugated system and causing a hypsochromic shift of fluorescence from 816 nm to 625 nm, a reduction in absorbance at 714 nm and 780 nm and the emergence of a new absorption band at 502 nm. **HTC-S** exhibited a wide linear range (0–30.0 μM), a low LOD of 0.10 μM, and a quick response (40 s). The probe was shown to be effective for monitoring Hg^2+^ residues on the surfaces of diverse food matrices and soil samples, as well as in water samples, through pronounced colorimetric and fluorescent changes. **HTC-S** was further loaded onto TLC plates and combined with a smartphone for quantitative analysis, revealing good linear relationships between the R/G and R/B ratios and Hg^2+^ concentrations in the range of 0–20 μM, thus providing a rapid on-site quantitative analysis method for Hg^2+^ detection. Cellular experiments confirmed that **HTC-S** exhibited low cytotoxicity (cell viability >90% at 10 mM) and mainly localized in mitochondria, as verified by the strong colocalization with Mito-Tracker Green (Pearson’s coefficient = 0.96). On this basis, **HTC-S** successfully achieved visual detection of Hg^2+^ in mitochondria of living cells: compared with the control group, after the addition of Hg^2+^, the red fluorescence signal in the NIR channel decreased, while the green fluorescence signal in channel 1 increased.

#### 3.2.4. NIR Fluorescent Probes Derived from Other Organic Dyes

**Figure 15:**
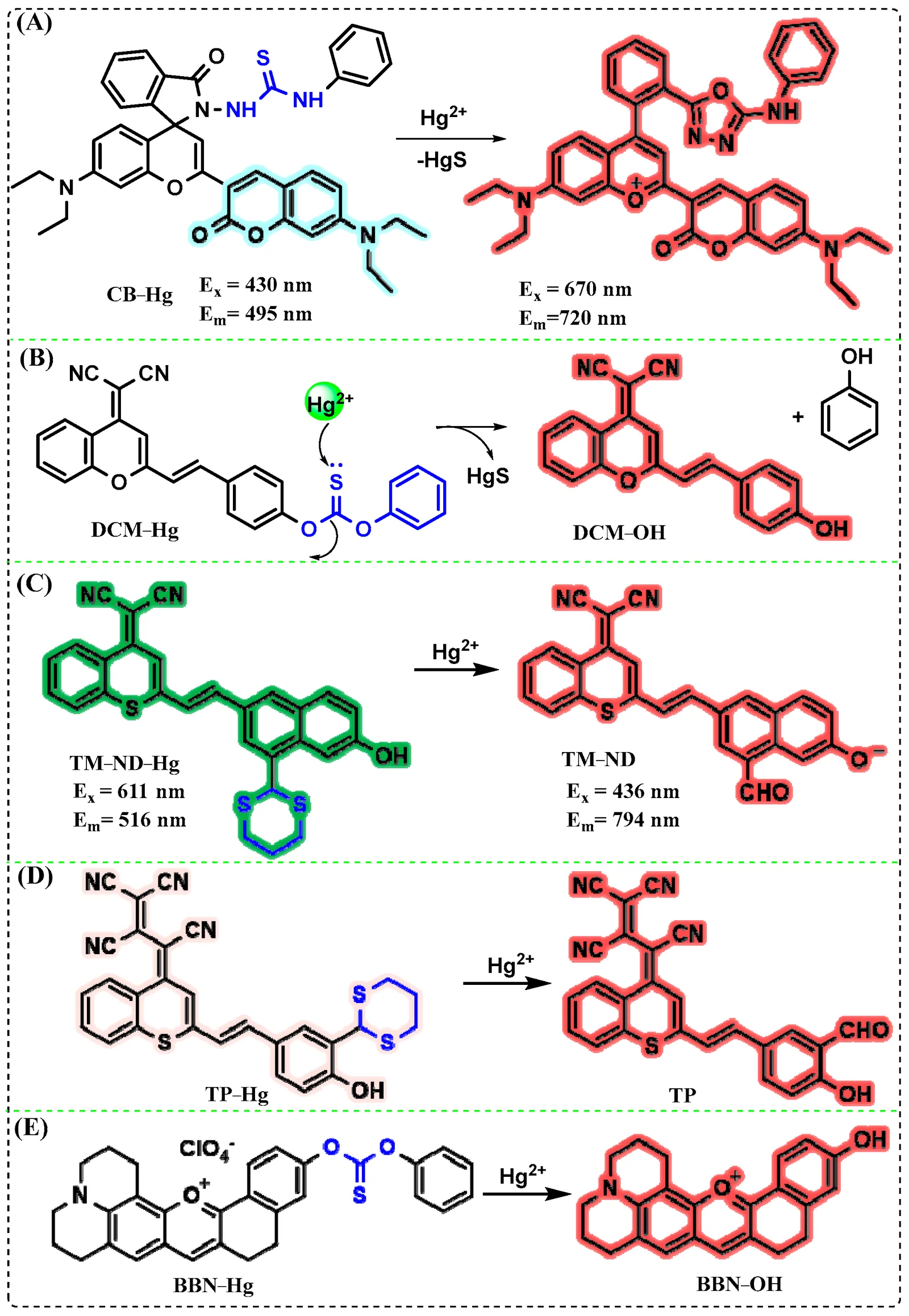
Structures and mechanisms of Hg^2+^ sensing by **CB-Hg** (**A**), **DCM-Hg** (**B**), **TM-ND-Hg** (**C**), **TP-Hg** (**D**) and **BBN-Hg** (**E**).

Apart from NIR probes constructed on rhodamine, cyanine, and isophorone frameworks, significant advances have also been made in the development of Hg^2+^ sensors using other NIR fluorophore platforms that operate via desulfurization-based reaction mechanisms, as illustrated in Figure 15 [128,129,130,131,132].

Lv’s group constructed a coumarin–benzopyrylium hybrid NIR probe **CB-Hg** for colorimetric and ratiometric Hg^2+^ sensing [128]. In aqueous solution, without Hg^2+^, the probe absorbed at 430 nm and emitted at 495 nm. Adding Hg^2+^ caused the absorption to red shift to 670 nm (Δλ = 240 nm), with a yellow-to-green color change, and produced a new NIR emission peak at 720 nm, while the 495 nm band diminished, enabling dual-channel ratiometric detection. **CB-Hg** showed extremely high sensitivity (LOD = 6.94 nM), excellent selectivity, a rapid response (30 s), and a 225 nm emission shift. The probe was further validated in tap water, mineral water, white rice, and live zebrafish, highlighting its practicality for Hg^2+^ monitoring in foodstuffs and biological specimens.

Zhou and colleagues constructed an NIR turn-on probe **DCM-Hg**, comprising a DCM fluorophore and a thiocarbonate recognition moiety [129]. Upon Hg^2+^-triggered desulfurization, the fluorophore DCM-OH was released, accompanied by prominent emission enhancement at 695 nm (LOD = 3.35 μM). With a Stokes shift of 145 nm that minimized self-absorption, **DCM-Hg** proved effective for fluorescence imaging of exogenous Hg^2+^ in HepG2 cells.

Utilizing a Hg^2+^-triggered thioacetal deprotection reaction, Zhang’s group designed an NIR probe **TP-Hg**, featuring a thiopyran–tetracarbonitrile core and a thioacetal-responsive unit [130]. Upon interaction with Hg^2+^, thioacetal deprotection triggered a marked turn-on NIR emission at 770 nm, with an LOD of 0.13 μM and an impressive 207 nm Stokes shift. Furthermore, **TP-Hg** possessed robust anti-interference capacity and naked-eye visual detection properties. Its practical versatility was demonstrated via successful fluorescence imaging of A549 cells and the fabrication of portable test strips for on-site Hg^2+^ monitoring, highlighting its potential for both biological imaging and environmental surveillance.

To further enhance detection accuracy, the group further designed **TM-ND-Hg**, a ratiometric fluorescent probe that integrated a benzothianone platform with a thioacetal group for detecting Hg^2+^ [131]. Interaction with Hg^2+^ caused a reduction in fluorescence at 516 nm and a concurrent enhancement at 794 nm, thereby achieving a ratiometric response with an LOD of 0.39 μM and an exceptionally large Stokes shift (358 nm). More importantly, **TM-ND-Hg**, in combination with smartphone-based RGB color recognition software, facilitated the portable on-site quantitative detection of Hg^2+^. Over the concentration range of 0–70 μM, both the R and B values demonstrated excellent linear relationships with Hg^2+^ concentration. In addition, the applicability of **TM-ND-Hg** was validated not only through the detection of Hg^2+^ in water samples, but also by fluorescence imaging of Hg^2+^ in living cells and organisms (zebrafish): as the Hg^2+^ concentration increased, the red-channel fluorescence signal in A549 cells and zebrafish was progressively enhanced, whereas the blue-channel fluorescence signal was gradually attenuated. These results suggest that **TM-ND-Hg** holds promise as an intelligent tool for rapid Hg^2+^ screening in complex matrices.

Employing a thiocarbonate recognition moiety similar to that of **DCM-Hg**, Fu’s group synthesized an NIR probe **BBN-Hg**, derived from an anthocyanin derivative, for visualizing Hg^2+^ [132]. Upon Hg^2+^-triggered cleavage, the probe released the fluorophore **BBN-OH** and the ICT effect was activated, producing a strong emission at 650 nm. **BBN-Hg** was employed for fluorescence imaging of Hg^2+^ in MCF-7 cells and zebrafish, with the red fluorescence signal displaying a concentration-dependent enhancement upon Hg^2+^ treatment. Remarkably, the probe was further applied to three-dimensional (3D) fluorescence imaging of zebrafish, thereby permitting direct visualization of the spatial distribution of Hg^2+^. Moreover, **BBN-Hg** proved useful not only for quantifying Hg^2+^ in real water samples (lake water, tea water, herbal extract) but also for the qualitative and quantitative analysis of Hg^2+^ in diverse soil types, while also enabling visualization of Hg^2+^ distribution in plant roots (Salvia miltiorrhiza and rice seedlings). Additionally, after immobilization on test strips, **BBN-Hg** allowed for qualitative recognition of Hg^2+^ through dual-modal colorimetric and fluorescent signals and could be coupled with smartphone-assisted RGB color analysis for on-site quantification. Taken together, **BBN-Hg** can provide a versatile tool for sensing and monitoring Hg^2+^ in both ecological and biological systems.

### 3.3. NIR Fluorescent Probes for Zn^2+^

As essential micronutrients, Zn^2+^ is critically involved in diverse biological processes, including enzymatic function, gene expression, and immune regulation [133]. However, imbalances in Zn^2+^ levels, whether deficiency or excess, are linked to serious health disorders, including developmental delays, neurodegenerative diseases, and gastrointestinal illness [134]. Beyond physiology, Zn^2+^ is widely used in industrial applications such as galvanization, battery production, and as a food additive. The environmental release of Zn^2+^ through industrial waste poses lasting ecological and health threats owing to its persistence and non-biodegradability [135]. Thus, the development of sensitive and selective monitoring methods for Zn^2+^ is crucial for understanding its biological roles and mitigating the related health and environmental impacts. Recently, large-scale NIR fluorescent probes with superior sensing performance have been developed to detect Zn^2+^. The photophysical properties of representative probes are summarized in Table 3.

#### 3.3.1. NIR Fluorescent Probes Derived from Dicyanoisophorone Derivatives

**Figure 16:**
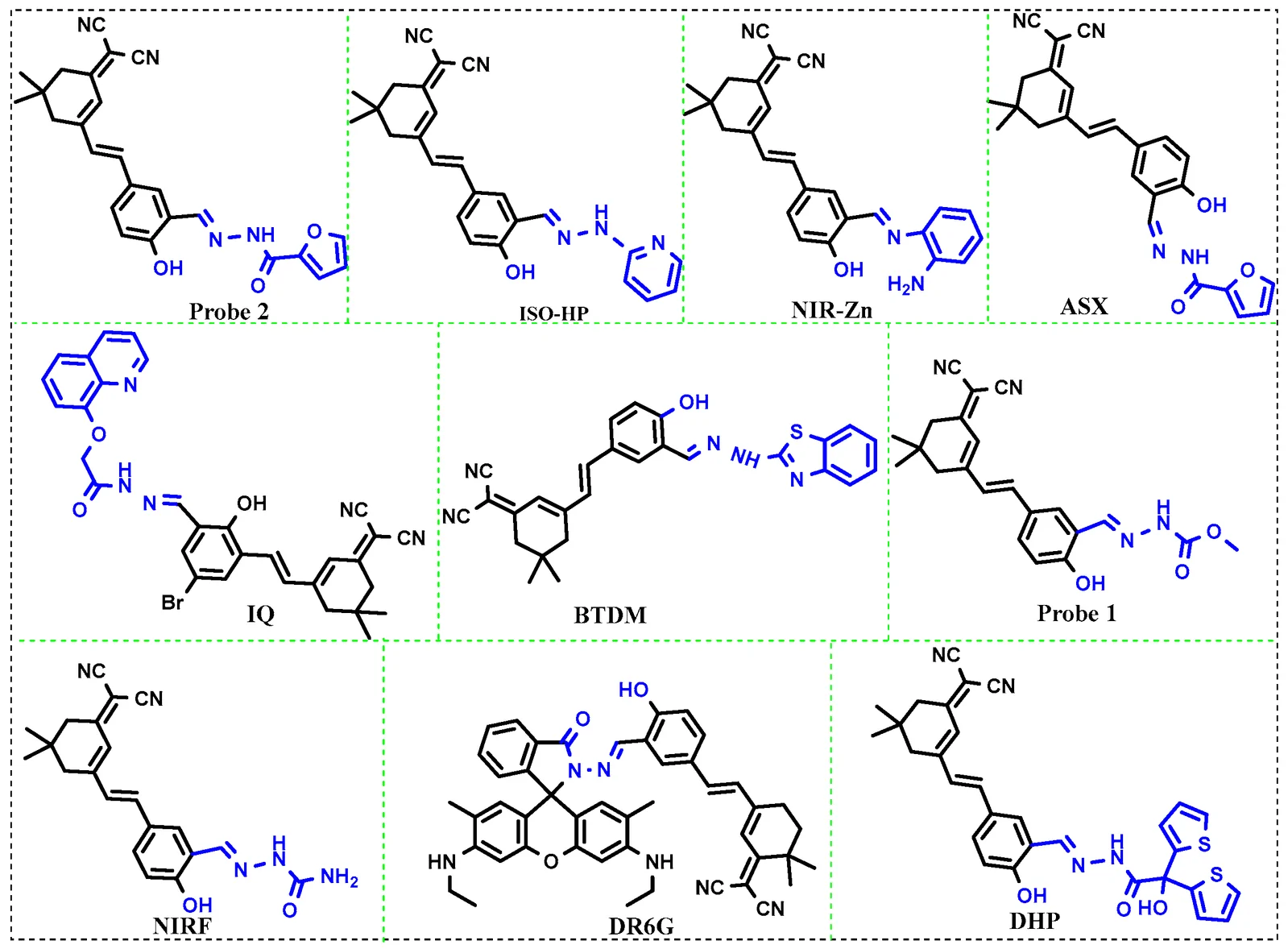
Structures of NIR fluorescent probes for Zn^2+^ based on dicyanoisophorone derivatives.

Dicyanoisophorone derivatives, which are characterized by their large planar conjugated structures, synthetic accessibility, and modifiable frameworks, have been widely exploited as NIR fluorophores. Based on the PET mechanism, numerous NIR fluorescent probes (Figure 16) have been developed for the selective detection of Zn^2+^ by introducing various recognition units into the dicyanoisophorone backbone, which exhibited superior sensing performance and hold significant promise for practical applications spanning environmental monitoring, food safety analysis and bioimaging [136,137,138,139,140,141,142,143,144,145].

In 2022, Yan, Wu and colleagues incorporated a furan-2-carbohydrazide moiety into the isophorone scaffold to afford **Probe 2** [136]. The probe initially displayed negligible fluorescence, which was attributed to the PET process from the C=N bond to the isophorone unit. Upon coordination with Zn^2+^ via the hydroxyl oxygen, acylamino oxygen, and imine nitrogen, the PET effect was effectively suppressed, causing a fold increase in emission at 670 nm. A good linear correlation (R^2^= 0.9911) was established between fluorescence intensity and Zn^2+^ concentration, and the LOD was calculated to be 2.7 nM. **Probe 2** featured high selectivity, reversibility, and successful application in real water sample analysis. Based on the same sensing mechanism, the group later reported **ISOHP**, which was synthesized via condensation of dicyanoisophorone with 2-hydrazinopyridine [137]. Structurally similar to **Probe 2**, **ISOHP** displayed a rapid turn-on response at 668 nm (within 15 s) upon 1:1 coordination with Zn^2+^ and Cd^2+^, with LODs of 0.21 μM and 0.31 μM, respectively. In contrast to **Probe 2**, **ISOHP** was further employed for fluorescence imaging in A549 cells and for visual detection using test strips.

To develop an NIR fluorescent probe with high selectivity for Zn^2+^ and suitability for bioimaging, in 2024, the team conjugated an o-phenylenediamine recognition group to a dicyanoisophorone scaffold, constructing the NIR probe **NIR-Zn** [138]. Binding Zn^2+^ in a 1:1 ratio suppressed the PET process, generating a sharp emission rise at 660 nm. **NIR-Zn** exhibited much higher selectivity for Zn^2+^ than for other metal ions, including Cd^2+^, along with a rapid response (<30 s), remarkable sensitivity (LOD = 4.8 nM), and a 179 nm Stokes shift. However, the coexistence of Cu^2+^, Co^2+^, and Ni^2+^ severely interfered with the detection signal of **NIR-Zn** toward Zn^2+^. Despite this interference, the probe was still effectively used for the quantitative detection of Zn^2+^ in pond water with excellent recovery and for confocal imaging of Zn^2+^ in A549 cells.

Similarly, in 2024, Miao’s team constructed **AXS** from the same dicyanoisophorone platform, functionalized with a furan-2-carbohydrazide moiety [139]. After specific binding to Zn^2+^, **AXS** displayed a prominent turn-on fluorescence response at 688.4 nm with outstanding sensing performance, including an exceptionally low detection limit (69.24 pM), a large Stokes shift (180 nm), good reversibility, high photostability, and pH insensitivity. **AXS** enabled both the qualitative and quantitative determination of Zn^2+^ in diverse real samples, including vegetables, fruits, and zinc gluconate oral solutions. Moreover, **AXS** was not only successfully used to fabricate portable test strips for visual on-site screening of Zn^2+^, but was also was coupled with a smartphone-assisted RGB analysis platform for accurate Zn^2+^ quantification with excellent sensitivity (LOD = 3.07 nM). Additionally, **AXS** was successfully employed for bioimaging of Zn^2+^ in Caenorhabditis elegans and mouse tissues (heart, liver, and kidney).

Erdemir et al. reported the NIR probe **IQ** for rapidly and selectively recognizing Zn^2+^ in environmental and biological specimens [140]. **IQ** exhibited weak intrinsic fluorescence, attributed to a PET state. Among various metal ions including Cd^2+^, Cu^2+^, and Hg^2+^, only Zn^2+^ significantly inhibited PET, increasing the fluorescence quantum yield of **IQ** from 0.056 to 0.40, which demonstrated good selectivity towards Zn^2+^. **IQ** was successfully loaded onto test strips, cotton swabs, and polysulfone capsules for convenient visual monitoring of Zn^2+^. With increasing Zn^2+^ concentration, a yellow-to-pink transition was observed under daylight, accompanied by a gradual enhancement in red fluorescence under UV light. Furthermore, when combined with smartphone-assisted RGB analysis, **IQ** enabled real-time quantitative detection in both colorimetric and fluorescent modes, with limits of detection of 0.30 μM and 0.21 μM, respectively. Its utility was further validated by Zn^2+^ analysis in water, food, and soil matrices. Finally, **IQ** successfully achieved the visual detection of Zn^2+^ in living cells, leveraging its NIR emission, large Stokes shift, and low cytotoxicity.

Recently, Qiao and Miao et al. constructed the probe **BTDM**, which possessed the same fluorophore scaffold as **IQ**, featuring a benzothiazole–hydrazine moiety as the Zn^2+^-specific recognition unit, for Zn^2+^ detection [141]. Only Zn^2+^ coordinated with **BTDM** in a 1:2 stoichiometric ratio via the imine nitrogen along with the benzothiazole nitrogen and sulfur atoms, triggering a pronounced turn-on emission at 683 nm. The probe was characterized by pH insensitivity (5–10), a remarkably large Stokes shift (248 nm) and detection limits of 34 nM (UV-Vis) and 18 nM (fluorescence). **BTDM** was subsequently fabricated into a multifunctional sensing toolkit, enabling Zn^2+^ detection through smartphones, test strips and electrospinning. These diverse platforms enabled **BTDM** to effectively quantify Zn^2+^ in actual foodstuff samples, such as apples, oranges, bananas, garlic and pears. Additionally, **BTDM**-functionalized electrospun fibers served as solid-state sensors for visual Zn^2+^ detection. More importantly, **BTDM** exhibited superior bioimaging capability and was employed for in vivo fluorescence imaging of Zn^2+^ in C. elegans and mice.

Beyond the pursuit of highly selective single-analyte probes, the integration of multifunctional recognition motifs into the isophorone platform has facilitated the development of NIR fluorescent sensors capable of simultaneous detection of Zn^2+^ and other anionic species. A representative example was reported in 2022 by Lei’s group, who constructed **Probe 1** by introducing a methyl hydrazate group onto a dicyanoisophorone skeleton via Schiff base reaction [142]. The probe initially displayed weak emission, arising from the combined PET effect of the C=N bond and inefficient ICT. Coordination with Zn^2+^ via N and O atoms inhibited both quenching pathways, yielding a rapid fluorescence turn-on at 653 nm (within 20 s), with an LOD of 15.3 nM. In contrast, HClO reacted with the C=N bond via addition, inhibiting PET while allowing for ICT, which induced an emission enhancement with a bathochromic shift from 670 nm to 705 nm (response time: 4 min; LOD: 1.39 μM). **Probe 1** was further validated by fluorescence imaging of Zn^2+^ and HClO in HepG2 cells and by recovery studies in environmental waters, highlighting its potential for multi-analyte applications in biological and environmental fields.

Yang’s group constructed an NIR fluorescent probe **NIRF** by incorporating semicarbazide hydrochloride into the dicyanoisophorone platform, enabling sequential detection of Zn^2+^ and CN^-^ [143]. Only Zn^2+^ could form a stable complex with **NIRF** (K_b_ = 2.06 × 10^5^), thereby causing significant fluorescence enhancement at 658 nm. Subsequent addition of CN^−^ triggered a ligand exchange reaction, displacing Zn^2+^ to form [Zn(CN)_x_] complexes and releasing free **NIRF**, resulting in distinct fluorescence quenching with an LOD of 7.9 × 10^−7^ M for CN^−^. Benefiting from its excellent sensing performance and low cytotoxicity, **NIRF** was successfully applied to the visual detection of Zn^2+^ and CN^−^ in living macrophages and mice via fluorescence imaging, as well as for the detection of both ions in real samples, highlighting its potential for dual-analyte sensing in complex systems.

Based on the FRET mechanism, in 2024, Yang’s group presented an NIR fluorescent probe **DR6G** for sequentially monitoring of Zn^2+^ and pyrophosphate (PPi) [144]. The probe consisted of rhodamine 6G hydrazide (donor) and a dicyanoisophorone derivative (acceptor). Zn^2+^ induced spiro-ring opening, producing a remarkable fluorescence emission at 646 nm with high sensitivity (LOD = 20.2 nM). The resulting **DR6G**–Zn^2+^ complex enabled PPi detection via fluorescence quenching (LOD = 20.6 nM), attributed to the strong binding between Zn^2+^ and PPi. **DR6G** was successfully used to visualize Zn^2+^ and PPi in living cells, mice, and Staphylococcus aureus, and to quantify Zn^2+^ in oral liquids and PPi in toothpaste.

Recently, Yu’s group developed **DHP**, a dicyanoisophorone-based NIR probe bearing a di(thiophen-2-yl)ethyl amide [145]. Owing to the intrinsic PET effect, **DHP** initially exhibited weak fluorescence. Upon 1:1 coordination with Zn^2+^ via the phenolic oxygen, imine nitrogen, and carbonyl oxygen atoms, the PET pathway was effectively suppressed and the CHEF effect was activated, giving rise to a pronounced NIR turn-on response at 660 nm. The probe featured an ultrafast response (4.7 s), a large Stokes shift (160 nm), and a low LOD of 0.13 μM for Zn^2+^, along with excellent selectivity and robust anti-interference capability. Notably, the **DHP**-Zn^2+^ complex could be further employed for the sequential detection of pyrophosphate (PPi) via competitive displacement of Zn^2+^, achieving an LOD of 0.39 μM and rapid response (6.8 s), thereby endowing the probe with dual-analyte sensing functionality. Beyond solution-based assays, **DHP** was successfully integrated into smartphone-assisted RGB analysis and portable swab tests for visual and on-site Zn^2+^ detection. Bioimaging experiments demonstrated that, in addition to the sequential imaging of exogenous Zn^2+^ and PPi in living cells (MCF-7 and HepG2), **DHP** also enabled the detection of Zn^2+^ in plant tissue (mung bean sprouts), highlighting its low background interference in complex biological tissues.

#### 3.3.2. NIR Fluorescent Probes Derived from Other Organic Dyes

**Figure 17:**
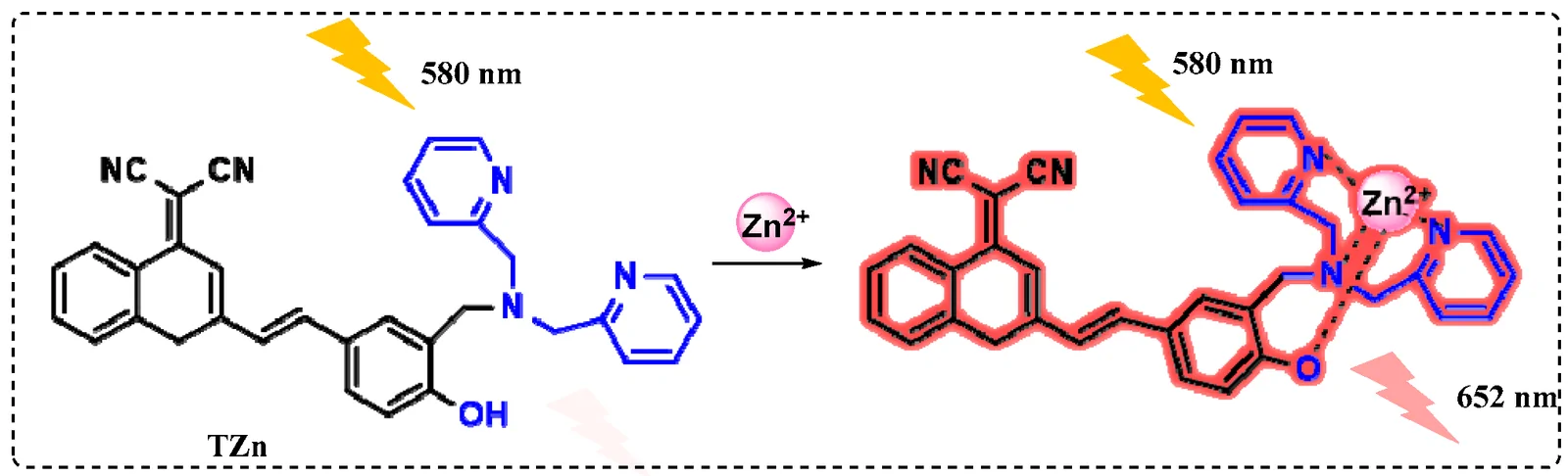
Structure and mechanism of **TZn** responses to Zn^2+^.

DCM derivatives are commonly employed as NIR fluorophores due to their favorable photophysical properties. Zhang’s group reported **TZn** (Figure 17), constructed on a DCM scaffold, by using a dipicolylamine (DPA) moiety as a specific recognition motif for Zn^2+^ [146]. The PET pathway of the probe **TZn** was effectively inhibited upon 1:1 chelation with Zn^2+^ via the DPA nitrogen and the phenolic oxygen, resulting in marked NIR fluorescence enhancement at 652 nm and a concomitant 30 nm red shift in the absorption band. **TZn** demonstrated outstanding selectivity for Zn^2+^ against various competing metal ions, achieving a detection limit of 0.31 μM and a fast response time of 40 s. Furthermore, the probe possessed favorable photostability, low cytotoxicity (cell viability >95% at 10 mM) and good biocompatibility, allowing for successful fluorescence imaging of Zn^2+^ in living HeLa cells and reliable quantification of Zn^2+^ in diverse food matrices (milk, water, red wine, and apple juice) with recoveries in the range of 92–103%.

**Figure 18:**
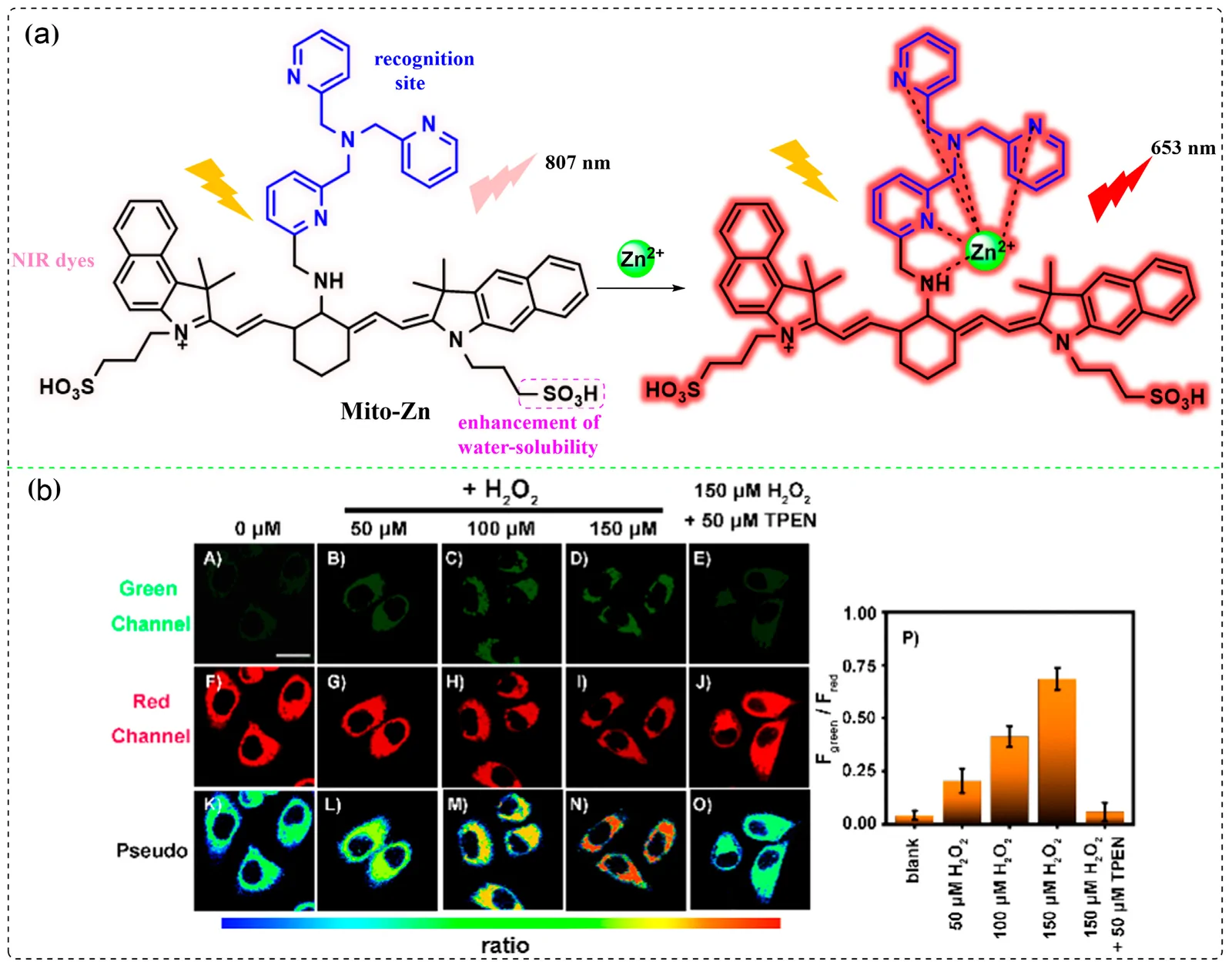
(**a**) Structure and mechanism of **Mito-Zn** responses to Zn^2+^; (**b**) fluorescence imaging of endogenous Zn^2+^ in HeLa cells. (**A**–**E**) Fluorescence images from the green channel (λem = 610–690 nm). (**F**–**J**) Fluorescence images from the red channel (λem = 760–840 nm). (**K**–**O**) Pseudo images obtained from the green and red channels. (**P**) The corresponding Fgreen/Fred. Scale bar: 20 μm. Reproduced with permission from Ref. [147]. Copyright (2024) Royal Society of Chemistry.

To quantitatively analyze Zn^2+^ in mitochondria, Huang and co-workers presented a ratiometric fluorescent probe **Mito-Zn** (Figure 18A) for mitochondrial Zn^2+^ detection [147]. The probe featured a cyanine-derived NIR fluorophore, a lipophilic cation for mitochondrial targeting, a pyridine-based Zn^2+^ recognition unit, and sulfo-groups for water solubility. Upon Zn^2+^ addition, **Mito-Zn** exhibited a distinct ratiometric response, characterized by a decrease in emission at 807 nm and a concomitant increase at 653 nm. The probe demonstrated high sensitivity (LOD = 26.7 nM), remarkable selectivity and low cytotoxicity (cell viability >92.4% at 60 mM). In addition, **Mito-Zn** mainly localized to mitochondria, as evidenced by strong colocalization with Mito-Tracker Green (PCC =0.88), enabling quantitative detection of exogenous Zn^2+^ in mitochondria. With increasing Zn^2+^ concentrations, the red-channel emission decreased gradually and the green-channel emission increased concomitantly, with the Fgreen/Fred ratio rising from 0.0425 to 0.8039. Additionally, it successfully visualized Zn^2+^ fluctuations in living cells under oxidative stress (Figure 18B) and notably revealed elevated Zn^2+^ levels in the brains of mice with depression.

### 3.4. NIR Fluorescent Probes for Pb^2+^

**Figure 19:**
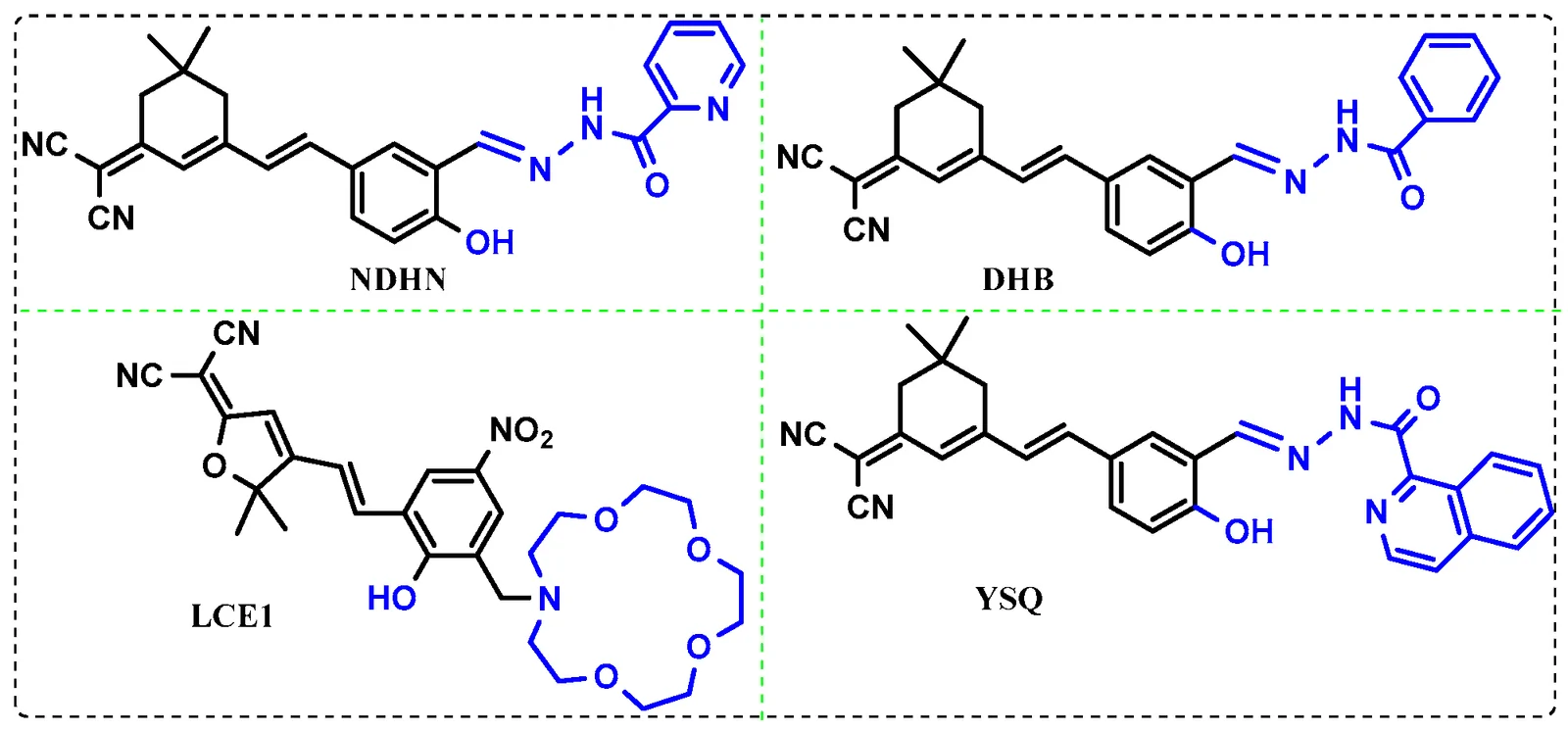
Structures of NIR fluorescent probes for Pb^2+^.

Lead ions (Pb^2+^), as a typical heavy metal pollutant, are extensively utilized in daily materials including gasoline, batteries, pigments, cosmetics, food additives, and toys, and can be accumulated in organisms through the environment and the food chain [148]. Given its high toxicity, persistence, and strong bioaccumulative nature, even trace Pb^2+^ exposure can cause irreversible damage to the nervous system, kidneys, and other vital organs [149]. In response, the WHO has set a maximum limit of 10 μM for Pb^2+^ in drinking water [14]. Therefore, developing simple, cost-effective, and rapid detection methods for Pb^2+^ in complex samples is of great significance. Recently, researchers have successively reported several Pb^2+^ detection systems based on NIR fluorescent probes (Figure 19), which demonstrate promising application potential in environmental monitoring and bioimaging owing to their superior photophysical properties and sensitive response mechanisms, as shown in Table 4 [150,151,152,153].

In 2024, Miao and colleagues reported the first fluorescent probe **NDHN** featuring both NIR emission and ratiometric fluorescence properties for sensing Pb^2+^ [150]. The probe consisted of a dicyanoisophorone scaffold, with a pyridine amide moiety serving as the Pb^2+^ recognition unit [150]. Coordination with Pb^2+^ inhibited the PET effect and C=N isomerization while enhancing the ICT effect, resulting in a red shift of the maximum absorption wavelength from 423 to 466 nm and a red shift of the fluorescence emission peak from 584 to 677 nm. **NDHN** exhibited high sensitivity and selectivity toward Pb^2+^, with minimal interference from competing ions. Leveraging these optical properties, a fluorescence/colorimetric/smartphone triple-mode sensing platform was constructed and successfully applied for Pb^2+^ detection in foods, including milk, milk powder and white wine. In smartphone mode, color intensity showed a good linear relationship with Pb^2+^ concentration over the range of 1–10 μM, with a detection limit of 130 nM. **NDHN** could be used for subcellular localization in HeLa cells and fluorescence imaging of exogenous Pb^2+^. The intracellular red fluorescence increased with increasing Pb^2+^ concentration and highly overlapped with the blue fluorescence of DAPI (PCC = 0.81). Notably, **NDHN** could be used for imaging Pb^2+^ in mouse organ cells, and **NDHN-Pb^2+^** exhibited excellent tumor-targeting ability in tumor-bearing mice, with the fluorescence intensity in tumor tissue being 5.1 times higher than that in the liver. Thus, NDHN offers a promising molecular tool for diagnosing Pb^2+^-related diseases.

Similarly, Zhao’s group successfully constructed a multifunctional NIR probe **DHB** based on the isophorone fluorescent scaffold, using benzohydrazide as the Pb^2+^ recognition group [151]. **DHB** exhibited an “off–on–off” fluorescence response, enabling sequential detection of Pb^2+^ ions and glyphosate (Glyp). **DHB** initially showed weak orange–red emission. Upon binding with Pb^2+^, the ICT effect was significantly enhanced, leading to a substantial increase in fluorescence intensity and a 70 nm red shift of the emission wavelength. Concurrently, the maximum absorption increased, accompanied by a 47 nm red-shift. Subsequent addition of glyphosate (Glyp) competitively removed Pb^2+^ from the **DHB**-Pb^2+^ ensemble, restoring the initial weak fluorescence. This reversible response enabled at least five sequential cycles. The probe showed high selectivity, excellent anti-interference capability, and high sensitivity for Pb^2+^ and Gly, with reported LODs of 35.8 mM and 36.5 mM, respectively, and a large Stokes shift of 220 nm. **DHB** was further utilized to quantitatively determine Pb^2+^ and Glyp in diverse real samples, including environmental water, soil extracts, and food matrices, with acceptable recoveries of 97.2–113.2% for Pb^2+^ and 90.1–102.8% for Glyp. Both **DHB** and the **DHB-Pb^2+^** complex showed low cytotoxicity, with >80% cell viability at 25 μM. In HeLa cells, **DHB** alone gave weak fluorescence, whereas Pb^2+^ induced concentration-dependent red fluorescence. Subsequent Glyp addition caused concentration-dependent fluorescence quenching. Similar phenomena were observed in rice root tips, zebrafish, and mouse models. Organ imaging suggested preferential hepatic accumulation of **DHB-Pb^2+^**, with stronger fluorescence in the liver than in other organs. These findings indicated that **DHB** may serve as a visual and potentially semi-quantitative probe for Pb^2+^ and Glyp in complex biological matrices.

In 2025, Meng’s group reported an NIR probe **LCE1** derived from a TCF platform functionalized with an azacrown ether as the Pb^2+^ recognition group [152]. Pb^2+^ coordinated with the N/O donor atoms of the azacrown ether and the oxygen atom of the phenolic, forming a 1:1 complex with **LCE1**, thereby suppressing the ICT process and resulting in fluorescence quenching at 670 nm. Fluorescence titration showed good linearity over the Pb^2+^ concentration range of 0–6 μM (R^2^ = 0.9917), with an LOD of 0.34 μM. **LCE1** could stably respond to Pb^2+^ over the pH range of 4.5–10.0, with a response time of 30 s, and exhibited strong anti-interference capability in the presence of coexisting ions such as Cd^2+^, Ag^+^, Cu^2+^, Hg^2+^, and so on. The probe enabled the quantitative determination of Pb^2+^ in real samples, including tap water, river water, lake water, black tea, and milk, with favorable recoveries (95.2–108.2%). In addition, **LCE1**-based test strips were used not only for semi-quantitative detection of Pb^2+^, as indicated by a color change from deep pink to pale orange with increasing Pb^2+^ concentration, but also, in combination with smartphone-based RGB analysis, for on-site rapid relative quantification of Pb^2+^ in water samples. The R/(G + B) ratio showed a linear correlation with Pb^2+^ concentration over the range of 0–300 μM (R^2^ = 0.9717). Furthermore, **LCE1** exhibited low cytotoxicity (cell viability >85% at 30 μM) and was successfully applied for fluorescence imaging of Pb^2+^ in MCF-7 cells, zebrafish, nude mice, and Pb-accumulating plants (*Chrysopogon zizanioides* (L.) Roberty and *Medicago sativa* L.), where Pb^2+^-dependent fluorescence attenuation was observed. This study suggests that **LCE1** holds promise for understanding the physiological roles of Pb^2+^, evaluating food safety, and screening plants for the phytoremediation of heavy metal-contaminated soils.

In addition, Liu and Xu et al. reported **YSQ**, an NIR probe built on an isophorone-isoquinoline scaffold [153]. In its free state, the probe showed weak emission owing to C=N isomerization and PET. However, coordination with Pb^2+^ effectively suppressed these quenching pathways and activated the CHEF effect, leading to a dramatic fluorescence enhancement at 678 nm. **YSQ** offered advantages of excellent selectivity, high sensitivity (LOD = 18 nM), fast response (1 min), and good reversibility. Based on these outstanding properties, **YSQ** was effectively employed to quantitatively detect Pb^2+^ in real water samples, imaging Pb^2+^ in living PK-15 cells, and visually sensing Pb^2+^ using test strips and SiO_2_ powder. Furthermore, with the aid of smartphone-assisted RGB color analysis, the probe enabled on-site visual screening of Pb^2+^.

### 3.5. NIR Fluorescent Probes for Cd^2+^

Cadmium (Cd^2+^) is a widely used industrial metal, employed in electroplating, smelting, battery manufacturing, and phosphate fertilizers. Its extensive application has led to the release of cadmium-containing wastewater, exhaust gases, and waste residues into the environment, contaminating soil and water through atmospheric deposition and irrigation [154]. Unlike other heavy metals, Cd^2+^ is readily absorbed by crops, such as rice, and undergoes significant bioaccumulation in plants due to its non-biodegradability. Through contaminated food, water, and air, Cd^2+^ enters the human body, posing serious health risks, including cardiovascular diseases, Alzheimer’s disease, kidney dysfunction, calcium metabolism disorders and Parkinson’s disease [155]. A typical example is Itai-Itai disease, caused by long-term consumption of cadmium-contaminated rice, which remains the primary route of human Cd^2+^ exposure [156]. The WHO has set a stringent limit of 3 ppb for Cd^2+^ in drinking water. Therefore, developing portable, efficient, and sensitive methods for Cd^2+^ detection is of great importance.

However, there have been few reports on NIR fluorescence probes for Cd^2+^ detection in recent years, primarily due to the following reasons. First, the chemical similarity between Cd^2+^ and Zn^2+^ poses a fundamental obstacle to highly selective recognition. Moreover, as an essential trace element for the human body, Zn^2+^ is ubiquitous in biological systems and far more abundant than Cd^2+^, making it difficult for probes to accurately discriminate Cd^2+^ in the presence of Zn^2+^. Second, the heavy-atom effect of Cd^2+^ itself drives probe design toward “turn-off” modes with weak signal changes. This intrinsic tendency makes the design of probes based on conventional “turn-on” mechanisms extremely challenging. Existing strategies are largely limited to “turn-off” responses or require ratiometric probes with higher synthetic difficulty to compensate for insufficient signal changes, thereby significantly restricting detection sensitivity and practical value. Third, Cd^2+^ is primarily regarded as an environmental pollutant, and its direct pathophysiological relevance is less clear than that of ions such as Zn^2+^ or Cu^2+^, resulting in relatively low research attention. Fourth, red-shifting the probe emission wavelength into the NIR region (650–900 nm) is itself a complex molecular engineering task, and on this basis, designing a suitable recognition group for Cd^2+^ requires simultaneously balancing the electronic structure of the fluorophore and the coordination ability of the recognition unit, leaving a narrow design window.

**Figure 20:**
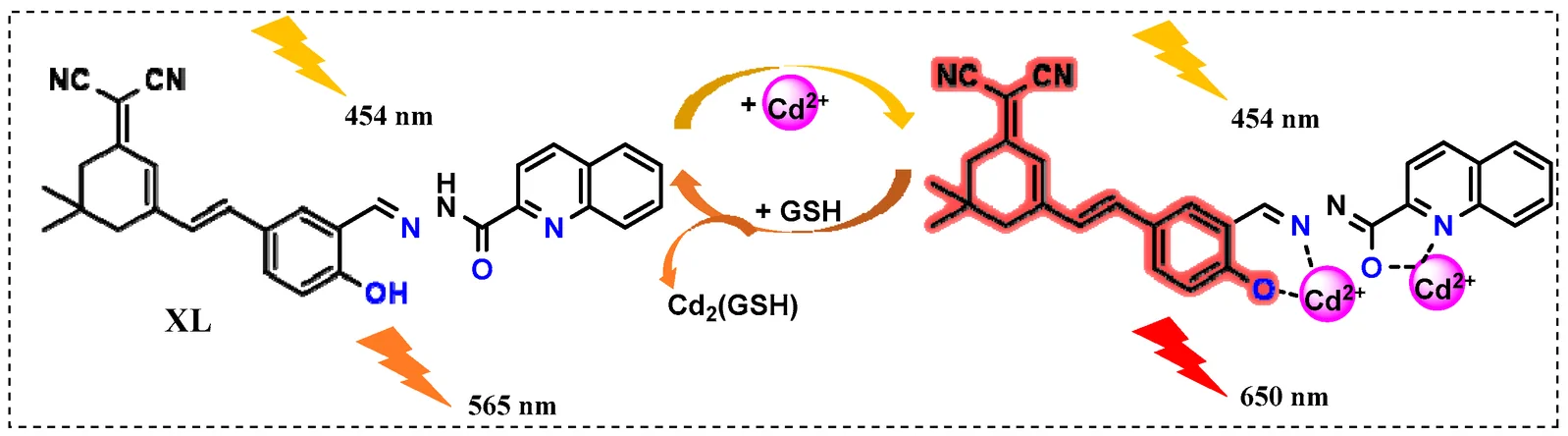
Structure and mechanism of **XL** responses to Cd^2+^ and GSH.

Given the complexity of environmental pollutants and the interconnectivity of metabolites within organisms, the development of multifunctional probes capable of the continuous detection of multiple analytes has become a research hotspot. In 2025, Zu and co-workers constructed the probe **XL** (Figure 20) by linking a dicyanodiphenyl isophorone derivative with a quinoline ring via a hydrazone bond, achieving continuous detection of Cd^2+^ and glutathione (GSH) [157]. The free probe emitted fluorescence at 565 nm. Upon binding with Cd^2+^ in a 1:2 molar ratio, the ICT process was activated, leading to a ratiometric fluorescence change, with the fluorescence intensity at 650 nm gradually increasing and progressively decreasing at 565 nm. When GSH was added to the **XL**-Cd^2+^ complex, the strong coordination ability of GSH competitively displaced Cd^2+^, releasing free **XL** and restoring fluorescence to 565 nm, while the solution color changed from purplish-red to yellow, enabling the detection of GSH with a detection limit of 0.784 μM. The probe demonstrates rapid response times, completing detection of both Cd^2+^ and GSH within 3 s. Based on its distinct color and fluorescence changes, the research team developed a smartphone-assisted visual detection platform, successfully applied for quantitative analysis of Cd^2+^ in water samples and GSH in garlic samples, providing a novel tool for rapid on-site detection.

### 3.6. NIR Fluorescent Probes for Fe^2+^/Fe^3+^

Iron (Fe^2+^/Fe^3+^) is an essential trace element involved in oxygen transport, DNA synthesis, metabolism, and immune defense. Intracellular iron exists mainly as redox-active Fe^2+^ in the labile iron pool [158]. Precise maintenance of iron homeostasis is critical for normal cellular function, and dysregulation of the Fe^2+^/Fe^3+^ balance can lead to serious health problems. Iron deficiency may cause iron-deficiency anemia and immune dysfunction, while iron overload induces oxidative damage via the Fenton reaction, leading to liver injury, neurodegeneration, cardiovascular disease, and cancer [159,160]. Moreover, excess Fe^2+^ drives ferroptosis, a key process in drug-induced liver injury and neurodegenerative diseases [161]. Thus, developing sensitive and selective methods for Fe^2+^/Fe^3+^ detection is crucial for investigating disease mechanisms and early diagnosis. NIR fluorescent probes, with their high spatiotemporal resolution and biocompatibility, have become powerful tools for real-time monitoring of iron dynamics in biological systems. The photophysical properties of representative probes are summarized in Table 5.

#### 3.6.1. NIR Fluorescent Probes Derived from Dicyanoisophorone Derivatives

**Figure 21:**
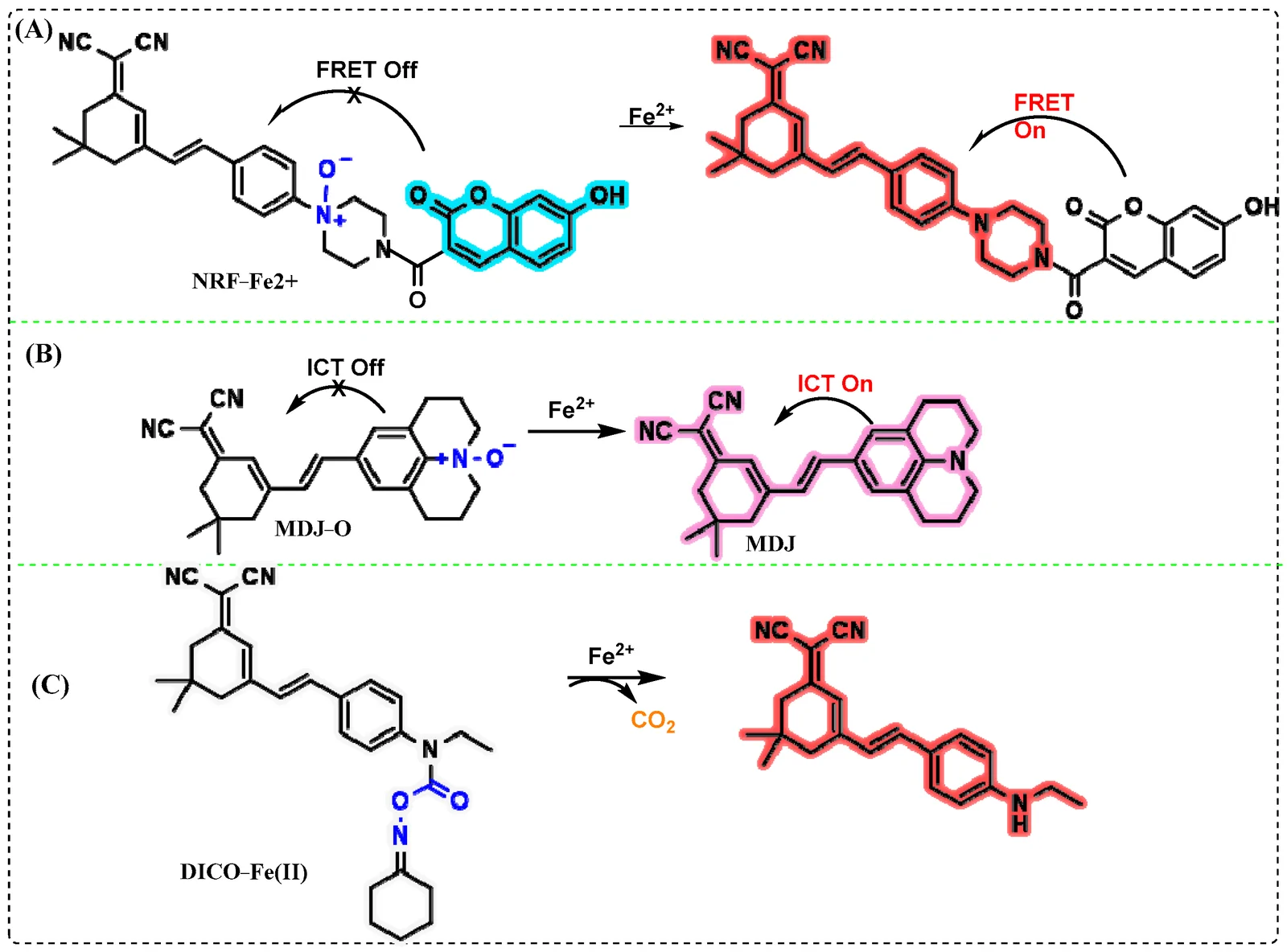
Structures and mechanisms of Fe^2+^ sensing by **NRF-Fe2+** (**A**), **MDJ-O** (**B**) and **DICO-Fe**(II) (**C**).

In recent years, leveraging the readily modifiable nature of the isophorone skeleton, researchers have successfully developed a variety of high-performance NIR fluorescent probes for Fe^2+^ detection and imaging. These probes, which operate through distinct response mechanisms, were constructed by structurally tuning the isophorone scaffold and introducing specific recognition moieties for Fe^2+^ (Figure 21).

To enable tracking of Fe^2+^ in iron-associated diseases in vivo, Feng’s group described a FRET-based ratiometric NIR probe **NRF-Fe^2+^** [162]. It consisted of a coumarin moiety as the energy donor and a dicyanoisophorone derivative as the acceptor, with an N-oxide group functioning as the Fe^2+^ recognition unit. Reduction of N-oxide by Fe^2+^ induced a marked emission shift from 460 nm to 675 nm, enabling ratiometric response with an LOD of 72 nM. The probe exhibited outstanding selectivity for Fe^2+^ over metal ions, ROS, RSS, saccharides, and amino acids, with negligible interference even in 10 mM GSH and operated over pH 2–8. **NRF-Fe^2+^** showed low cytotoxicity (A549 viability >82% at 0–50 μM) and enabled ratiometric imaging of exogenous and endogenous Fe^2+^ in living cells, where blue fluorescence gradually decreased with prolonged Fe^2+^ exposure, while red fluorescence was enhanced. DFO (deferoxamine mesylate, a Fe^2+^ chelator) reversed this signal. Moreover, similar phenomena were also observed when NRF-Fe2+ was employed to monitor Fe^2+^ dynamics during Erastin-induced ferroptosis. Notably, **NRF-Fe^2+^** also detected elevated Fe^2+^ in acetaminophen (APAP)-induced liver injury, with fluorescence intensity approximately twice that of controls. As one of the few NIR ratiometric probes for tracking Fe^2+^ in ferroptosis and DILI, NRF-Fe^2+^ holds promise for Fe^2+^-related disease research, although it has a slow response (~30 min) and requires multi-step synthesis.

**Figure 22:**
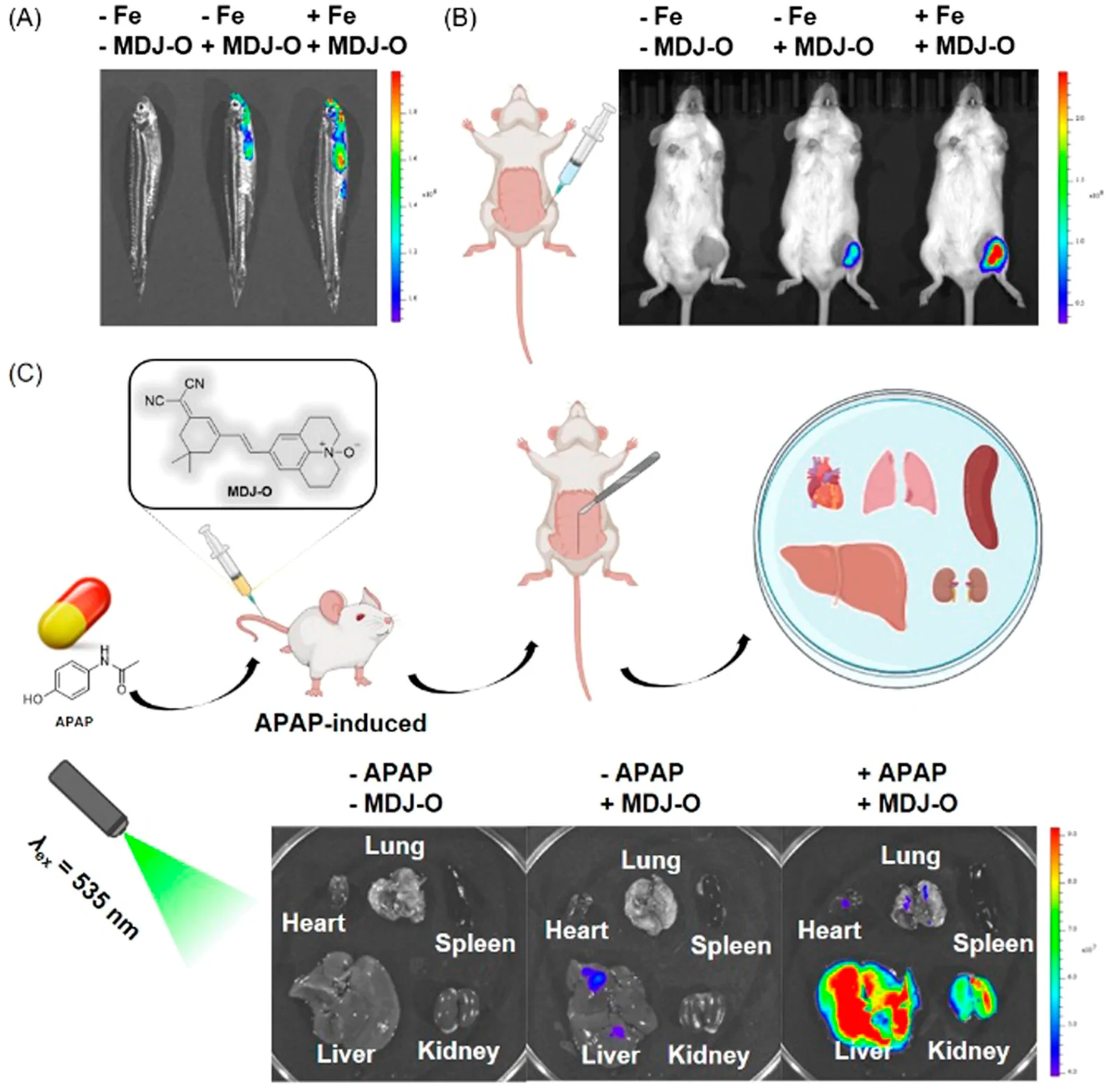
(**A**) Fluorescence imaging of endogenous Fe^2+^ in zebrafish; (**B**) fluorescence imaging of exogenous Fe^2+^ in living mice; (**C**) fluorescence imaging of main organs of APAP-induced mice model. Reproduced with permission from Ref. [163]. Copyright (2024) Royal Society of Chemistry.

To address the slow response of many Fe^2+^ probes, in 2024, Xiao et al. introduced **MDJ-O**, an NIR probe featuring an isophorone-based fluorophore and an N-oxide unit on the julolidine ring as the Fe^2+^-responsive moiety [163]. Upon exposure to Fe^2+^, **MDJ-O** was converted to the strongly emissive **MDJ**, yielding a 49-fold increase in fluorescence intensity at 720 nm (l_ex_ = 535 nm) and a red shift of absorption peak from 390 nm to 550 nm within 5 min. The probe enabled both colorimetric and fluorometric detection of Fe^2+^ in nearly 100% aqueous medium. **MDJ-O** exhibited high selectivity for Fe^2+^ over other analytes, without interference except for Cu^2+^, and showed a pronounced response over pH 4–9. It had low cytotoxicity (cell viability > 90% at 10–30 μM), good intracellular photostability, and was mainly localized in lysosomes (PCC = 0.88, significantly higher than 0.56 for mitochondria). The successful fluorescence imaging of exogenous and endogenous Fe^2+^ in cells and imaging of Fe^2+^ in zebrafish in a concentration-dependent manner were achieved by using **MDJ-O**. More importantly, **MDJ-O** could detect both exogenous and endogenous Fe^2+^ in mice and visualize elevated Fe^2+^ levels in APAP-induced liver and kidney injury models (Figure 22), where fluorescence in the liver and kidneys was markedly stronger than in other organs. 2,2′-bipyridine (Bpy) pretreatment significantly suppressed this enhancement, confirming that the signal originated from Fe^2+^. Therefore, **MDJ-O** offers a promising tool for investigating drug-induced organ damage and iron-related diseases, although its capacity for Fe^2+^ sensing is still compromised by Cu^2+^ interference and a relatively high detection limit (3.09 mM).

Later, to enable specific and sensitive monitoring of Fe^2+^ in food and biological systems, Zhu and Sheng et al. rationally engineered an NIR fluorescent probe **DICO-Fe(II)**, incorporating a carbamoyl oxime recognition moiety and an isophorone-based fluorophore [164]. Unlike conventional N-oxide-based probes, the carbamoyl oxime group underwent Fe^2+^-mediated N–O bond cleavage, restoring the ICT effect and generating a turn-on emission at 666 nm within 8 min. **DICO-Fe(II)** exhibited high selectivity for Fe^2+^ over common cations, anions, reactive oxygen species, and biothiols, with negligible interference. Fluorescence titration showed excellent linearity over the Fe^2+^ concentration range of 0–50 μM (R^2^ = 0.9917), with an LOD of 47 nM. Concurrently, the solution changed from colorless to tawny, enabling on-site visual detection via smartphone-assisted RGB analysis. Benefiting from its excellent sensitivity and selectivity, **DICO-Fe(II)** was successfully applied to the accurate determination of Fe^2+^ in food samples (spinach, rape, bean sprouts, and chicken) with acceptable recoveries of 89.87–110.22%. Moreover, the probe exhibited negligible cytotoxicity and excellent cell membrane permeability, enabling fluorescence imaging of Fe^2+^ in cells, where fluorescence intensity increased with Fe^2+^ concentration and was significantly suppressed by Bpy. **DICO-Fe(II)** also enabled visualization of Fe^2+^ in living zebrafish in a concentration-dependent manner. Notably, it allowed for fluorescent imaging of elevated Fe^2+^ levels in a mouse model of APAP-induced liver injury, whereas NAC treatment markedly reduced the signal, suggesting its potential as an effective tool for studying Fe^2+^ dynamics in drug-induced liver injury.

#### 3.6.2. NIR Fluorescent Probes Derived from Other Organic Dyes

**Figure 23:**
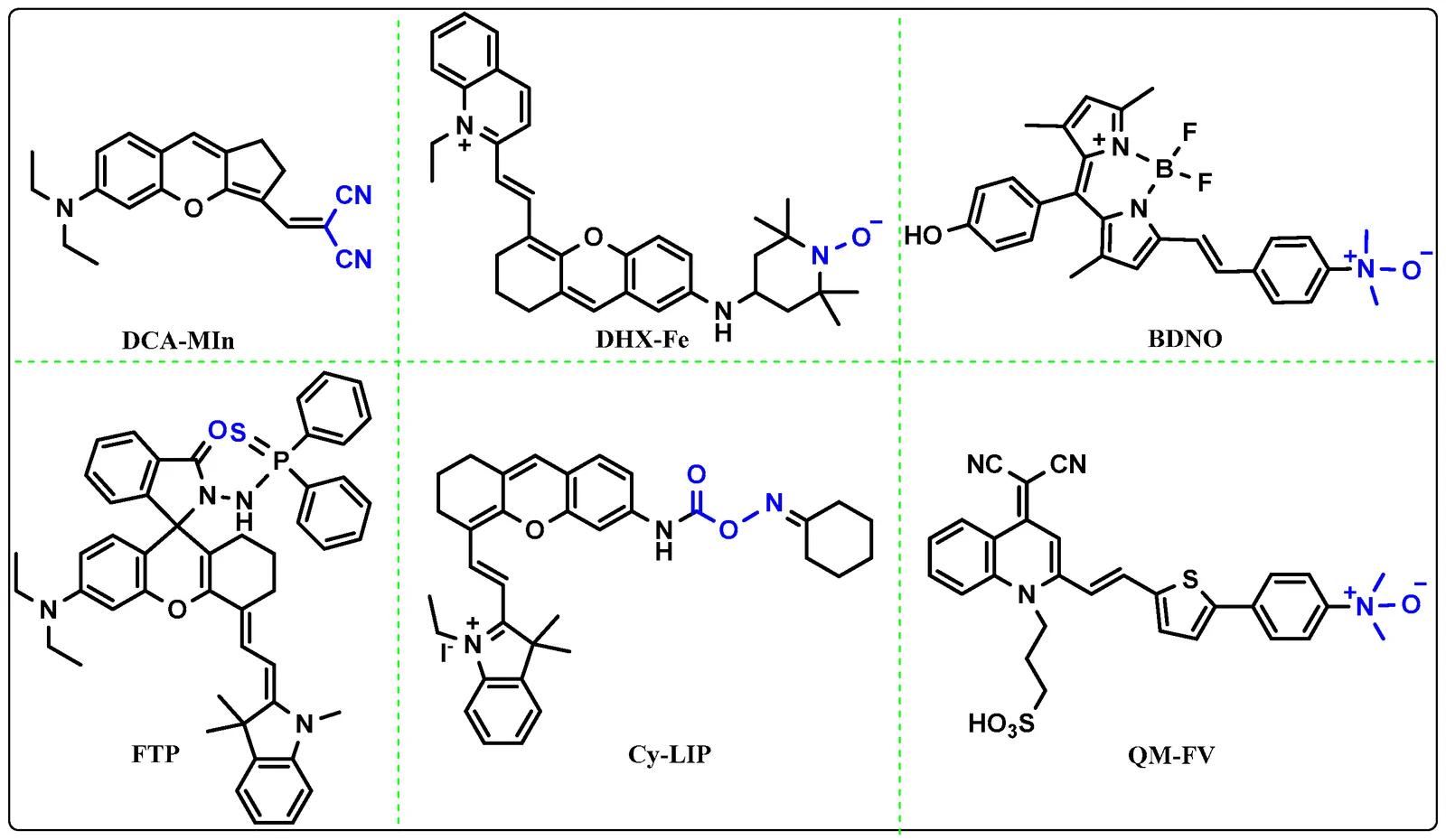
Structures of NIR fluorescent probes for Fe^2+^/Fe^3+^.

Taking advantage of the reducing property of Fe^2+^ and the strong coordination ability of Fe^3+^ with nitrogen and oxygen atoms, the researchers introduced specific recognition groups such as N-oxide and carbamoyl oxime into near-infrared fluorophores with large π-conjugated structures and successfully constructed a range of iron ion NIR fluorescent probes with excellent performance (Figure 23) [165,166,167,168,169,170].

In 2022, Tang’s group obtained **DCA-Mln**, a dual-channel colorimetric and NIR probe with a D-π-A framework, by coupling a dicyanovinyl group with a DCA fluorophore via the Knoevenagel condensation reaction [165]. DCA-MIn emitted strongly at 670 nm. While the fluorescence was efficiently quenched by Fe^3+^. Concurrently, a distinct color change from purple to peach pink was observed, allowing for naked-eye detection, and the solution color changed from purple to peach pink. The sensing mechanism was based on a 2:1 binding stoichiometry between **DCA-Mln** and Fe^3+^, which suppressed the ICT effect and triggered the PET process. **DCA-Mln** demonstrated excellent selectivity, a fast response (within 15 s) and a low LOD of 19.5 mM. Its applicability was further demonstrated in real water sample analysis, fluorescence imaging of Fe^3+^ in living MCF-7 cells, and anti-counterfeiting ink preparation.

To improve the sensitivity of NIR fluorescent probes for Fe^2+^ detection, He’s group designed an NIR probe **DHX-Fe** in 2025 [166]. The probe employed a quinoline–xanthene scaffold as the NIR emissive unit and a nitroxide radical as the responsive group for Fe^2+^. In the absence of Fe^2+^, the nitroxide radical quenched the fluorescence of **DHX-Fe** through electron transfer. Upon reaction with Fe^2+^, the nitroxide radical was reduced to hydroxylamine, triggering a 9.4-fold enhancement at 690 nm (λ_ex_ = 610 nm). Apart from Fe^2+^, **DHX-Fe** showed a negligible response to common metal ions, biological reductants, reactive oxygen species, and CYP450, and their coexistence does not interfere with Fe^2+^ detection. Furthermore, the probe displayed a fast response (<5 min) and high sensitivity (LOD = 115 nM) towards Fe^2+^ and operated effectively at a wide pH range (3–8). In addition, **DHX-Fe** exhibited low cytotoxicity (cell viability >80% at 50 μM) and excellent mitochondrial targeting capability (PCC = 0.89), enabling concentration-dependent fluorescence imaging of exogenous Fe^2+^ in A2780 and SKOV3 cells, with the fluorescence signal significantly reduced upon addition of Bpy or DFO. In cisplatin-resistant ovarian cancer cells (A2780/DPP), the probe was used to evaluate Fe^2+^ changes induced by various ferroptosis inducers, revealing that Brusatol markedly elevated mitochondrial Fe^2+^, which was reversed by Fer-1. Mechanistic studies showed that Brusatol induced ferroptosis by increasing Fe^2+^, ROS, and lipid peroxidation (LPO), decreasing GPX4, and increasing ACSL4. Furthermore, in A2780/DPP tumor-bearing nude mice, **DHX-Fe** enabled in vivo imaging of exogenous Fe^2+^ and dynamically monitored the sustained elevation of intratumoral Fe^2+^ during Brusatol treatment (days 7–14). Pharmacokinetic studies indicated hepatic and renal metabolism, and H&E staining confirmed no obvious toxicity in major organs. Therefore, as the first mitochondria-targeted NIR Fe^2+^ probe for evaluating ferroptosis inducers in chemoresistant ovarian cancer, **DHX-Fe** provides a powerful visual tool for overcoming tumor drug resistance.

To further improve the response speed and detection accuracy, based on the nitroxide radical reduction mechanism, Dutta et al. described **BDNO**, featuring a BODIPY scaffold modified with an N-oxide recognition unit for Fe^2+^ [167]. The ICT pathway of **BDNO** was suppressed by the N-oxide group, resulting in yellow fluorescence emission at 570 nm (λ_ex_ = 540 nm). Upon addition of Fe^2+^, the original emission decreased while new emission at 715 nm (λ_ex_ = 570 nm) increased, enabling self-calibrating ratiometric detection. **BDNO** offered an ultrafast response (<5 s), excellent selectivity, and high sensitivity (LOD = 41 nM), and responded effectively to Fe^2+^ over pH 4–10. Its practical utility was confirmed by the quantitative detection of Fe^2+^ in diverse food samples and environmental water samples with satisfactory recoveries. Furthermore, **BDNO**-functionalized paper strips and cotton buds allowed for on-site visual detection with the naked eye via both colorimetric and fluorescent modes without the need for specialized equipment. In addition, a smartphone-integrated test strip platform enabled quantitative Fe^2+^ detection with recoveries of 91.5–96.28%. These results suggest that **BDNO** holds promise for food safety monitoring and environmental water analysis. However, the reduced product BDN suffered from poor solubility in high-water-content systems, which may limit its NIR imaging applications in pure aqueous environments and biological systems.

Later, Jiang and colleagues reported a chromenylium–cyanine probe **FTP** for Fe^3+^ sensing via a spirolactam ring opening mechanism [168]. **FTP** was synthesized by conjugating a modified rhodamine scaffold with a diphenylphosphine moiety through a hydrazine bridge. In the solution of EtOH/PBS (*v*/*v*, 3/7), coordination with Fe^3+^ induced spirolactam ring opening, causing a sharp increase in fluorescence intensity at 743 nm and a distinct color change from pale yellow to green, enabling both fluorometric and colorimetric detection with LODs of 24.0 nM (fluorescence) and 79.7 nM (UV-Vis). In addition, **FTP** showed excellent selectivity and reversibility, a rapid response (<2 min), outstanding anti-interference, and favorable stability at pH of 4–10, enabling quantitative analysis of Fe^3+^ in water samples with good recoveries, as well as visual qualitative detection on filter paper and visual semi-quantitative detection on cotton swabs. Its applicability was further confirmed by fluorescence imaging of living cells and mice. Moreover, compared with the aforementioned probes, **FTP** exhibited improved aqueous solubility and sensitivity, but remains inadequate for bioimaging applications.

Recently, Fang’s group successfully constructed a water-soluble carbamoyl oxime-based NIR probe, **Cy-LIP**, utilizing an Fe^2+^-mediated reductive N–O bond cleavage mechanism [169]. In PBS solution, upon Fe^2+^ activation, **Cy-LIP** released a hemicyanine fluorophore, producing an 18-fold fluorescence increase at 705 nm. **Cy-LIP** showed high selectivity, rapid response kinetics (<20 min), high sensitivity (LOD = 51 nM), and a broad pH range (5–9). It successfully detected both exogenous and endogenous Fe^2+^ in cells and monitored Fe^2+^ dynamics during erastin/RSL3-induced ferroptosis, with malondialdehyde levels paralleling the Fe^2+^ signal, further confirming ferroptosis. **Cy-LIP** was mainly localized in mitochondria, showing a high PCC of 0.91 with Mito-Tracker Green, much higher than that with lysosomes (PCC = 0.43) or the endoplasmic reticulum (PCC = 0.51). Importantly, **Cy-LIP** detected elevated Fe^2+^ levels in murine models of rheumatoid arthritis (RA) and DILI. In the RA model, fluorescence increased progressively over time. In the DILI model, the APAP-treated group showed significantly higher fluorescence than the control, while NAC treatment markedly reduced the signal. Thus, **Cy-LIP** offers a versatile tool for studying iron-related disorders.

To simultaneously visualize changes in Fe^2+^ and intracellular viscosity during pathophysiological processes of hepatocellular carcinoma (HCC), in 2025, Hong, Qiu and Feng et al. synthesized a dual-detection probe **QM-FV**, utilizing an N-oxide moiety for Fe^2+^ recognition and a molecular rotor for viscosity sensing [170]. Upon Fe^2+^ addition, **QM-FV** exhibited marked fluorescence turn-on at 740 nm within 150 s, with a 30-fold fluorescence enhancement. The probe was effective over pH 3–8 and showed high selectivity toward Fe^2+^ over common metal ions, anions, amino acids, biothiols, and ROS/RNS. Meanwhile, elevated viscosity triggered a distinct orange–red emission enhancement at 587 nm, thereby enabling dual-channel independent responses to Fe^2+^ and viscosity. Owing to its high sensitivity (LOD = 25 nM), large stroke shift (290 nm), NIR emission, dual response and low cycotoxity, **QM-FV** enabled imaging of exogenous and endogenous Fe^2+^ in living cells and monitored the simultaneous elevation of intracellular Fe^2+^ and viscosity induced by monensin and nystatin. Moreover, the probe effectively discriminated cancer cells from normal counterparts, with both green and red channels showing markedly stronger fluorescence in cancer cells. Importantly, **QM-FV** was successfully applied to image Fe^2+^ and viscosity changes during erastin-induced ferroptosis and cisplatin-induced apoptosis, as well as in a mouse model of HCC, revealing a progressive increase in both parameters during disease progression. As the first dual-responsive probe capable of simultaneously monitoring Fe^2+^ and viscosity, **QM-FV** provides a powerful tool for investigating Fe^2+^- and viscosity-related pathological processes in HCC.

### 3.7. Cross-Comparison Among Different Metal Ions

In the above discussion of research progress on heavy metal ions, we first classified the probes by metal ion species and then summarized the research progress on target ions based on different NIR fluorescent platforms. To help readers better understand the latest advances and remaining challenges in this field, we cross-compared different metal ions from the following aspects and discussed the challenges they share.

In terms of fluorophore selection, NIR fluorescent probes for Cu^2+^, Hg^2+^, Zn^2+^, Pb^2+^, Cd^2+^ and Fe^2+^/Fe^3+^ show a certain preference. NIR fluorescent probes for Cu^2+^ and Hg^2+^ are often based on rhodamine, dicyanoisophorone, and hemicyanine/cyanine scaffolds; Zn^2+^ probes commonly employ isophorone and cyanine scaffolds. Pb^2+^ probes are frequently constructed on a dicyanoisophorone scaffold. Fe^2+^/Fe^3+^ probes often combine reactive recognition groups such as N-oxide or carbamoyl oxide with isophorone or xanthene scaffolds. Overall, rhodamine, dicyanoisophorone, hemicyanine and cyanine are the four most commonly used scaffolds for constructing NIR heavy metal ion probes.

From the perspective of recognition mechanisms, reversible coordination-based probes and irreversible reaction-based probes each have advantages and limitations. Zn^2+^, Pb^2+^, and some Cu^2+^ and Hg^2+^ probes mostly adopt reversible coordination mechanisms, which offer the advantages of reusability, a relatively fast response, and suitability for real-time imaging, but they may suffer from interference by metal ions with similar chemical properties. For example, the probe **CL(Hg^2+^)** can respond to both Hg^2+^ and Ag^+^. Fe^2+^ probes, some Cu^2+^ probes, and some Hg^2+^ probes employ irreversible reaction-based mechanisms. For instance, Cu^2+^ can induce picolinate ester hydrolysis or spirolactam ring opening of rhodamine. Hg^2+^ can induce thioacetal desulfurization, spirolactam ring opening of rhodamine, or thioester hydrolysis. Fe^2+^ can reduce N-oxide or hydrolyze carbamoyl oxide. Irreversible reaction-based probes offer advantages such as high selectivity and irreversible signal accumulation, but they may suffer from a relatively slow response, lack of reusability, and more complex evaluation of in vivo metabolism and toxicity.

In terms of interfering ions, Cd^2+^/Zn^2+^ and Hg^2+^/Ag^+^ are two of the most challenging ion pairs. Zn^2+^ and Cd^2+^ have similar ionic radii and coordination chemistry, and some Zn^2+^ probes are susceptible to Cd^2+^ interference, and vice versa. Hg^2+^ and Ag^+^ also share similar thiophilicity, and some Hg^2+^ probes produce false positive signals in the presence of Ag^+^. In addition, Fe^2+^ probes are sometimes interfered with by Cu^2+^ (e.g., probe **MDJ-O**). Therefore, high selectivity remains one of the core challenges in the design of NIR heavy metal ion probes.

For practical applications, the development of probes for different metal ions is uneven. NIR probes for Cu^2+^, Hg^2+^, and Zn^2+^ have been reported more extensively and have been widely used in water samples, food, cells, and in vivo imaging, whereas NIR probes for Cd^2+^, Pb^2+^, and Fe^2+^/Fe^3+^ are relatively scarce. Most probes still rely on organic cosolvents (e.g., DMSO, DMF, EtOH and acetonitrile) and have limited water solubility, which restricts their application in environmental water samples and on-site detection.

In bioimaging, most probes remain at the level of cytotoxicity evaluation, and systematic studies on biocompatibility, biodistribution, metabolism, long-term toxicity, clearance pathways, and subcellular targeting are lacking. Although some probes have been applied to cell, zebrafish, mouse, and plant imaging, cases that truly achieve clinical translation or environmental on-site application remain rare. In addition, the emission wavelengths of most probes are confined to the NIR-I region (650–900 nm), while NIR-II (1000–1700 nm) probes remain very rare, limiting the resolution and penetration depth of deep-tissue imaging.

## 4. Comparative Analysis of Fluorophore Platforms

Although the preceding sections have systematically reviewed the research progress of NIR fluorescent probes categorized by metal ions, it is evident that the same fluorophore platforms (e.g., isophorone, rhodamine, hemicyanie and cyanine) have been repeatedly employed for the detection of different ions. Therefore, an in-depth comparison of the intrinsic merits and limitations of these platforms is crucial for guiding the rational design of future probes.

Dicyanoisophorone (DCI)-based fluorophores are highly favored owing to their well-defined D–π–A architecture, which confers excellent ICT characteristics. Consequently, DCI-based probes generally possess large Stokes shifts (typically exceeding 150 nm, with some exceeding 300 nm), effectively avoiding excitation light scattering and self-absorption interference while enhancing detection sensitivity. Moreover, their core structure is readily amenable to modification through π-conjugation extension or heteroatom substitution, enabling precise tuning of the emission wavelength into the NIR region. Despite these advantages, DCI-based fluorophores also have limitations. The most prominent issue is their poor water solubility, which to some extent restricts their direct application in pure aqueous systems and compromises detection efficiency in biological environments. In addition, DCI-based probes often exhibit relatively low fluorescence quantum yields, which adversely affects the signal-to-noise ratio and imaging contrast. In terms of sensing mechanisms, many DCI-based probes rely on irreversible chemical reactions (e.g., Cu^2+^-induced hydrolysis or Hg^2+^-triggered desulfurization), thereby limiting their ability to monitor dynamic fluctuations of target ions in real time. Furthermore, although the emission wavelengths of DCI platforms have entered the NIR region, the emission peaks of most probes remain below 700 nm. Thus, compared with the NIR-II window (1000–1700 nm), there is still room for improvement in tissue penetration depth.

Rhodamine derivatives, owing to their unique spirolactam structure, serve as an ideal platform for constructing “turn-on” fluorescent probes. In the free state, rhodamine-based probes exist in a colorless and non-fluorescent spirocyclic form with extremely low background signals. Upon binding to specific analytes (such as metal ions), the spirolactam ring opens, accompanied by pronounced fluorescence enhancement and naked-eye color changes, thereby providing a natural “switch-on” signal for highly selective detection. Rhodamine dyes also have high fluorescence quantum yields, large molar extinction coefficients, and excellent photostability, endowing them with high sensitivity and good reproducibility in analytical detection. In recent years, by extending the π-conjugated system, introducing strong electron-donating groups, or constructing hybrid scaffolds (for example replacing the oxygen atom in the xanthene core with silicon or carbon), researchers have successfully red-shifted the emission wavelengths of rhodamine derivatives into the NIR region (>650 nm) while retaining their spirocycle ring opening response mechanism, significantly expanding their potential for bioimaging. However, rhodamine dyes generally suffer from small Stokes shifts, leading to severe self-absorption, low signal-to-noise ratios, and insufficient deep-tissue penetration, which seriously hampers their application in complex systems such as food detection, environmental monitoring, and bioimaging. In addition, the spirocycle ring opening process of many rhodamine-based probes is irreversible, preventing probe reuse and making it difficult to continuously monitor dynamic changes in target ion concentrations. Finally, rhodamine-based probes in complex biological matrices may also be constrained by environmental factors (e.g., acidic conditions) and background interference.

Cyanine dyes are among the most widely studied and applied NIR fluorophores. Their principal advantages include extremely high molar extinction coefficients (typically >10^5^ L·mol^−1^·cm^−1^), good fluorescence quantum yields, and facile tuning of absorption and emission into the NIR region (650–900 nm) through structural modification of terminal heterocycles and meso-substituents on the methine chain. They also generally show good biocompatibility, and certain dyes, such as IR-780, even exhibit intrinsic tumor-targeting ability. However, cyanine dyes have inherent drawbacks. Insufficient photostability is the most critical bottleneck. Under illumination, they readily undergo photooxidative degradation, mechanistically involving regioselective C–C cleavage of the methine chain by singlet oxygen to form a dioxetane intermediate that subsequently decomposes, leading to fluorescence signal decay and deteriorated probe performance, particularly in long-term imaging experiments. Poor water solubility is another key issue limiting their biological applications. Basic cyanine dyes are highly lipophilic and require modifications such as sulfonation to prepare water-soluble derivatives (e.g., Sulfo-Cy7) suitable for aqueous systems. At high concentrations, cyanine dyes also tend to self-aggregate, causing aggregation-caused quenching (ACQ) and restricting their use in scenarios requiring high probe concentrations. In terms of chemical stability, cyanine dyes are sensitive to reactive oxygen and nitrogen species and may undergo nonspecific degradation in complex biological environments. Many cyanine-based probes also rely on irreversible chemical reactions induced by target ions (e.g., carbothioate hydrolysis or desulfurization), which similarly limits reversibility.

Hemicyanine scaffolds, as asymmetric derivatives of cyanine dyes, combine NIR emission with greater molecular design flexibility. Their core advantage also stems from a unique D–π–A structure. By introducing different recognition groups, the ICT process can be effectively modulated to achieve “turn-on,” ratiometric, or quenching-type fluorescence responses toward target ions. Hemicyanines generally exhibit larger Stokes shifts (some exceeding 200 nm), effectively avoiding self-absorption and excitation light scattering interference and thereby improving detection sensitivity. In addition, hemicyanine dyes generally show good mitochondrial targeting ability, conferring unique advantages for organelle-level imaging. In terms of reversibility, some hemicyanine probes have demonstrated good reversible response capability, enabling multiple detection cycles while maintaining stable performance. However, the emission wavelengths of hemicyanine-based fluorophores are generally shorter than those of cyanines, with maximum absorption mostly limited to 650–720 nm and emission to 680–750 nm, resulting in relatively limited tissue penetration depth. Additionally, although the D–π–A structure of hemicyanines offers flexible design space, some probes still suffer from relatively low fluorescence quantum yields, especially in the long-wavelength emission region, which may affect imaging contrast. Traditional hemicyanine dyes also exhibit relatively insufficient photostability and pronounced solvatochromic effects, which limit their application in cellular microenvironment detection to some extent.

Overall, these four representative fluorophore platforms each have distinct characteristics, and their performance features show a clear correspondence with suitable application scenarios. DCI-based probes, with their large Stokes shifts and tunable emission wavelengths, are suitable for applications extremely sensitive to self-absorption and background interference, but water solubility and quantum yield remain their shortcomings. Rhodamine-based probes offer low-background advantages unmatched by other platforms through their spirocycle switch mechanism, performing excellently in high-SNR detection. However, irreversibility and relatively small Stokes shifts limit their dynamic monitoring capability and deep-tissue imaging applications. Cyanine-based probes, with long excitation/emission wavelengths and high molar extinction coefficients, occupy an irreplaceable position in deep-tissue imaging and photodynamic therapy, but their photostability and water solubility require systematic optimization. Hemicyanine platforms are molecularly flexible, and their D–π–A structure makes them ideal templates for constructing activatable probes, with intrinsic mitochondrial targeting ability, although their wavelength and quantum yield still need improvement.

Rather than pursuing a single “perfect” platform, future development of fluorophore platforms can be advanced in four complementary directions. First, hybrid fluorophores should be designed. This can be achieved, for example, by combining the D–π–A architecture of DCI with the long-wavelength emission of cyanines while exploiting the spirocycle ring opening mechanism of rhodamines to achieve highly selective responses. Second, water solubility should be optimized. The aqueous compatibility of DCI and cyanine platforms can be improved by introducing hydrophilic groups such as sulfonate, quaternary ammonium, or PEG chains. Third, reversibly responsive NIR fluorescent probes should be developed. For instance, probes based on competitive coordination or redox-reversible reactions can be designed to enable real-time tracking of dynamic target ion fluctuations. Fourth, the emission wavelength should be extended into the NIR-II region. Emission beyond 1000 nm can be achieved by further extending π-conjugation and introducing strong electron-donating groups, thereby meeting the demands of deeper tissue imaging. The synergistic implementation of these strategies is expected to advance NIR fluorescent probes for heavy metal ion detection from “capable of detection” to “precise, dynamic, and field-deployable detection.”

## 5. Summary and Outlook

Excessive accumulation of heavy metal ions in the environment poses a critical hazard to both ecological balance and public health. Consequently, achieving accurate quantification of these pollutants and elucidating their correlation with disease mechanisms carries considerable scientific significance and societal benefits. In recent years, NIR small-molecule fluorescent probes have become powerful tools for heavy metal ion monitoring, with notable strides made since 2022. This review provides a comprehensive and updated overview of NIR organic fluorogenic probes developed for the detection of major heavy metals, specifically Cu^2+^, Hg^2+^, Zn^2+^, Pb^2+^, Cd^2+^, Fe^2+^, and Fe^3+^. We focus particularly on the molecular design strategies, response modes, analytical performance, and biological imaging utilities of representative sensors for these seven ions. A range of high-performance NIR probes have been successfully engineered by leveraging fluorophore cores such as isophorone, rhodamine, heptamethine cyanine, and DCM derivatives, coupled with the incorporation of target-specific recognition units. The sophistication of probe design continues to advance, with sensing mechanisms encompassing a variety of photophysical pathways, including PET, ICT, FRET, CHEF, and ESIPT. The emission wavelengths of these probes generally exceed 650 nm, and in certain cases, Stokes shifts exceeding 200 nm have been achieved, substantially mitigating autofluorescence interference and markedly improving the signal-to-noise ratio in imaging applications.

From the current perspective, the development of NIR fluorescent probes exhibits the following trends. (1) The function of the probe has evolved from detecting a single target to simultaneously identifying multiple analytes. Researchers have developed multifunctional probes capable of sequentially detecting multiple targets, providing powerful tools for multi-factor correlation analysis in complex systems. For example, the NIR probe **QA** presented by Dong’s group enabled the sequential detection of Cu^2+^ and GSH; **W-2a** developed by Wang et al. responded to Hg^2+^ and ONOO^−^ with dual fluorescence signals, serving as an effective tool for studying the correlation mechanism between intracellular Hg^2+^ and ONOO^−^; **DNB** constructed by Zhao and colleagues achieved the sequential detection of Pb^2+^ and the pesticide glyphosate both in living cells and in vivo. (2) The application of probes has expanded from laboratory analyses to on-site rapid detection. By integrating probes with portable technologies such as smartphone-based RGB recognition, visual test strips, and SiO_2_ powder-based detection platforms, NIR fluorescent probes have been successfully applied for on-site quantitative screening of heavy metal ions in complex matrices like real water samples, food, soil, and residues on the surface of traditional Chinese medicines, significantly enhancing detection convenience and practicality. (3) The application of fluorescent probes in imaging has progressively advanced from the cellular level to multi-level in vivo imaging, including plant tissues (e.g., roots of soybean, rice, vetiver, alfalfa), model organisms (e.g., zebrafish, Caenorhabditis elegans), and mammalian in vivo imaging (e.g., mice, nude mice). Some probes have even achieved 3D fluorescence imaging and tumor-targeted imaging. For instance, Zhang’s team utilized **VIC** to perform, for the first time, 3D fluorescence imaging analysis of the relationship between Cu^2+^ accumulation and disease progression in the brain tissue of an AD mouse model, providing a visual tool for studying the distribution, migration, and toxicity mechanisms of heavy metal ions in living organisms. (4) The practical application of probes has progressively extended from fundamental research to the exploration of disease mechanisms. Some probes have been successfully applied for the dynamic monitoring of relevant metal ions (Cu^2+^, Zn^2+^) in disease models such as Alzheimer’s disease, Parkinson’s disease, and depression, revealing the correlation between ion homeostasis imbalance and disease progression, thus opening new avenues for early disease diagnosis and mechanistic studies.

Despite the fruitful achievements in NIR fluorescent probes for heavy metal ions, several challenges in their development and translation into practical applications remain. First, the ability to achieve high-specificity recognition and anti-interference towards chemically similar ion pairs, such as Cd^2+^/Zn^2+^ and Hg^2+^/Ag^+^, remains insufficient. Second, most probes remain limited to cytotoxicity evaluation, and systematic studies on biocompatibility, biodistribution, metabolism, long-term toxicity, and subcellular targeting are still lacking. Third, matrix effects in complex samples, such as soil extracts, food matrices, and traditional Chinese medicine surfaces, can compromise detection accuracy, and robust signal correction strategies are urgently needed. Fourth, photostability, storage stability, water solubility, reproducibility, and cost remain important factors limiting on-site detection and commercialization. Fifth, emission wavelengths of most probes are confined to the NIR-I region, while NIR-II region (1000–1700 nm) probes remain scarce. Sixth, since 2022, no NIR fluorescent probes for As detection have been reported, and only one NIR probe for Cr^3+^ detection has been documented, indicating that the coverage of target analytes remains imbalanced. Finally, integrating probes into test strips or smartphone devices for portable on-site detection also requires further optimization of probe loading, signal stability, and readout methods.

Therefore, the following issues should be given more considerations in future: (1) Designing and synthesizing new fluorophores with emission wavelengths exceeding 1000 nm using strategies like expanding conjugated systems and introducing strong electron-donating groups to achieve high-resolution deep-tissue imaging. (2) Constructing smart responsive probes integrated machine learning-assisted design that can respond to specific ion and possess signal self-calibration or logic operations capabilities. (3) Developing multi-mode imaging probes that combine fluorescence imaging with photoacoustic imaging, magnetic resonance imaging and other modalities to achieve comprehensive visualization from the molecular to the whole-body level. (4) Promoting the development of standardized detection kits and portable detection devices for practical needs such as environmental pollution monitoring, food safety screening, and occupational disease prevention. (5) Deepening the application of probes in mechanism studies of complex diseases like neurodegenerative and metabolic disorders to systematically investigate the dynamic changes and regulatory mechanisms of heavy metal ions, providing new targets and strategies for disease diagnosis and treatment. (6) Expanding the scope of target metal ions by developing highly selective and sensitive NIR fluorescent probes for highly toxic metal ions such as arsenic and chromium. It is also necessary to simultaneously improve probe stability, water solubility, biocompatibility, and targeting ability and to construct standardized portable detection platforms.

In summary, NIR fluorescent probes demonstrate immense application potential and promising development prospects in the field of heavy metal ion detection. With the interdisciplinary integration of materials science, chemical biology, artificial intelligence, and other fields, it is believed that in the future, an increasing number of NIR fluorescent probes for heavy metal ions with superior performance, enhanced functionality, and broader applications will continue to emerge, providing powerful molecular tools for environmental monitoring, food safety, disease diagnosis, and life science research.

## Figures and Tables

**Table 1:** The photophysical properties of NIR fluorescent probes for Cu^2+^.

Name	Solvent	Ex (nm)	Em (nm)	Sensing Mechanism	RESPONSE TIME	REVERSIBLE	Signal Transduction Mode	Detection Limit	pH Range	Application	Ref.
** DDP-Cu **	PBS/DMSO = 8/2, *v*/*v*	520	665	ICT	-	No	turn-off	36 nM	3–7.45	SH-SY5Y cells, HeLa cells, zebrafish, mice	[78]
** DCM-CuCl **	PBS /DMSO = 1/4, *v*/*v*	520	668	ICT	2.5 min	-	turn-on	0.13 μM	-	HeLa cells, tap water, Yangtze river water, Jialing river water	[79]
** CL(Cu^2+^) **	DMSO/H_2_O = 9/1, *v*/*v*	435	704/ 580	ICT	2.5 min	-	ratiometric	1.70 μM	4.0–11.0	L929 cells	[80]
** QA **	DMSO/PBS =3/7, *v*/*v*	425	688	CHEQ	-	Yes	turn-off	35.5 mM	-	HepG2 cells, C. elegans, mice, tap water, lake water	[81]
** DCPO-Cu **	DMF/HEPES = 7/3, *v*/*v*	420	655	ICT	<3 min	-	turn-on	0.018 mM	7.0–9.0	River water, tap water, mung bean sprouts, rice, onion roots, soil samples	[82]
** VIC **	DMF/HEPES = 1/9, *v*/*v*	425	675	CHEQ	30 s	-	turn-off	7 nM	5.0–12.0	Sandy soil, loess soil, forest soil, senna alexandrina mill, reed leaves, Chinese chives, garlic bolts, MCF-7 cells, mice	[83]
** 1(Cu^2+^) **	CH_3_CN/H_2_O = 2/1, *v*/*v*	520/ 650	580/ 770	ICT	-	No	ratiometric	9 nM	4.0–8.0	HepG2 cells, paulownia sprout	[84]
** NRh-NP **	DMSO/ HEPES = 1/1, *v*/*v*	660	750	PET	20 s	Yes	turn-on	33 nM (fluorescence) 35 nM (UV)	6.5–8.0	MCF-7 cells, mice	[85]
** NRh6G-FA **	DMSO/ HEPES = 1/1, *v*/*v*	665	744	PET	60 s	Yes	turn-on	0.225 μM	7.0–9.5	MCF-7 cells, Astragalus Paeonia lactiflora	[86]
** NRK-BD **	MeCN/H_2_O = 7/3, *v*/*v*	560	730	PET	1.5 s	Yes	turn-on	81 nM	6.5–8.5	tap water, Tianyi Lake, Yellow river, samples, brassica chinensis, potatoes, carrots, 4 T1 cells, mice	[87]
** NRTB **	EtOH	600	765	PET	300 s	-	turn-on	22.6 nM	-	Cherry tomatoes, cucumbers, apples, cucumbers, 4T1 cells, mice	[88]
** LDMD-N **	PBS/CH_3_CN = 2/1, *v*/*v*	615	775	ICT	5 min	NO	turn-on	0.0543 µM	-	SH-SY5Ycells, zebrafish, mice	[89]
** CySH **	HEPES/EtOH = 1/1, *v*/*v*	694	784	PET	40 min	-	turn-on	124 nM	4.0–9.0	HepG2 cells	[90]
** PhenIR **	ACN/H_2_O = 1/1, *v*/*v*	750	808	CHEQ	-	Yes	turn-off	0.286 mM	4.0–8.2	WS1 fibroblast cells	[91]
** CyCu1 **	HEPES/ DMSO = 99/1, *v*/*v*, 0.1% CrEL	710/ 523	810/ 600	ICT	40 min	NO	ratiometric	0.053 mM	-	SH-SY5Y cells, mice, Arabidopsis root	[92]
** NUST-Cy-1 **	THF/H_2_O = 6/4, *v*/*v*	450/ 750	593/ 825	FRET	-	-	ratiometric	1.4 mM	-	Drinking water, HeLa cells, mice	[93]
** HX **	-	730	810	CHEF	-	Yes	turn-on	0.54 mM	-	Live cells, mice	[94]
** HC-TPA **	Tris	604	668	CHEQ	1 min	No	turn-off	48 nM	7.0–10.0	Apple, watermelon, grape Peach, lychee, tomato, HEK293T cells	[95]
** DDAO-Cu **	PBS	602	662	ICT	<3 min	NO	turn-on	1.8 nM	-	PC12 cells, zebrafish, mice	[96]
** DCX-Cu **	PBS/DMSO = 1/1, *v*/*v*	590	746	ICT	10 min	No	turn-on	19.47 nM	6–10	Fountain pool water, tap water, wastewater, HepG2 cells	[97]
** NS-5 **	PBS/DMSO = 95/5, *v*/*v*	650	720	PET	20 min	-	turn-on	32 nM	-	HepG2 cells, LO2 cells, mice	[98]

Note: Probe names are shown in bold.

**Table 2:** The photophysical properties of NIR fluorescent probes for Hg^2+^.

Name	Solvent	Ex (nm)	Em (nm)	Sensing Mechanism	Response Time	Reversible	Signal Transduction Mode	Detection Limit	pH Range	Application	Ref.
** HgS **	PBS/DMSO = 7/3, *v*/*v*	430	645	ICT	3 min	No	turn-on	64 nM	6.5–8.5	Bottled water, river water, tap water, deionized water, HeLa cells, mice	[102]
** CL(Hg^2+^) **	EtOH/H_2_O = 1/1, *v*/*v*	379	695	ICT	<2 min	-	turn-on	0.83 mM	2.0–12.0	U87MG cells	[103]
** N-FP **	EtOH/H_2_O = 9/1, *v*/*v*	498	661	ICT	1 min	No	turn-on	0.72 mM	1.0–10.0	-	[104]
** DSX **	EtOH/PBS = 1/9, *v*/*v*	470	670	TICT	-	No	Turn-off	6.57 nM	-	Dawen river water, Yellow river water, Jinxiu river water, fish, apple, HeLa cells, zebrafish	[105]
** NIRPC-Hg **	HEPES/ACN = 9/1, *v*/*v*	420	666/535	ICT-ESIPT	120 min	No	ratiometric	9.21 nM	-	Tap water, Dawen river water, Yellow river water, HepG2 cells, zebrafish	[106]
** YF-Hg **	DMF/PBS = 5/5, *v*/*v*	468	659	ICT	12 min	No	turn-on	0.297 mM	7.0–10.0	Mouse fibroblasts	[107]
** W-2a **	DMSO/PBS = 1/1, *v*/*v*	500	656	ICT	25 min	No	turn-on	97 nM	-	EMT6 cells, mice	[108]
** THI **	MeCN/HEPES = 2/8, *v*/*v*	500	645	ICT	-	Yes	turn-on	7.22 mM	-	HepG2 cells	[109]
** 1(Hg^2+^) **	MeCN/HEPES = 4/1, *v*/*v*	440	660	PET	30 s	Yes	turn-on	5.6 nM	7.5–9.5	Pond water, river water, HepG2 cells	[110]
** RBLY **	HEPES/EtOH = 5/1, *v*/*v*	620	695	Hg^2+^-induced spirolactam ring opening	120 s	Yes	turn-on	0.34 mM		Pure water, Mo Chou lake water, Yin Xing lake water, MCF-7 cells, zebrafish, soybean rhizoma	[111]
** RBLS **	EtOH/HEPES = 1/3, *v*/*v*	620	685	Hg^2+^-induced spirolactam ring opening	210 s	Yes	turn-on	0.26 mM	5.0–10.0	Xuanwu Lake water, Zixi Lake water, HeLa cells, zebrafish	[112]
** CCS-Hg **	PBS/EtOH = 3/2, *v*/*v*	680	780	Hg^2+^-induced spirolactam ring opening	<30 s	-	turn-on	73 nM	5.0–9.0	A549 cells	[113]
** Rho-Hg **	PBS/EtOH = 1/1, *v*/*v*	610	665	Hg^2+^-induced spirolactam ring opening	<1 min	-	turn-on	17.5 nM	2.0–10.0	Taozi lake water, Houhu lake water, tap Wate, HeLa cells, zebrafish	[114]
** JZ-1 **	PBS	700	728	Hg^2+^-induced spirolactam ring opening	30 min	No	turn-on	33 nM	-	HeLa cells, zebrafish, mice	[115]
** CSME **	ACN/HEPES = 5/1, *v*/*v*	660	711	Hg^2+^-induced spirolactam ring opening	90 s	-	turn-on	4.93 nM (fluorescence) 58.9 nM (UV)	-	Tap water, drinking water, 3T3 cells	[116]
** CIME **	ACN/HEPES = 5/1, *v*/*v*	719	750	spirocyclic Hg^2+^-induced spirolactam ring opening	30 s	-	turn-on	0.65 nM (fluorescence) 37 nM (UV)	-	Tap water, drinking water, lake water, MCF-7 cells, 3T3 cells	[117]
** NIR8 **	DMF/HEPES = 5/1, *v*/*v*	670	755	Hg^2+^-induced spirolactam ring opening	15 s	No	turn-on	11.3 nM (fluorescence) 49.3 nM (UV)	-	Tap water, drinking water, lake water, IH/3T3 cells, MCF-7 cells	[118]
** NIR9 **	ACN/Tris = 1/1, *v*/*v*	715	750	Hg^2+^-induced spirolactam ring opening	10 s	-	turn-on	0.02 mM (fluorescence) 0.01 mM (UV)	-	Tap water, drinking water, lake water, cocoa, mushroom, sunflower seeds, A549 cells	[119]
** NIR-2 **	Acetone/HEPES = 4/1, *v*/*v*	719	755	Hg^2+^-induced spirolactam ring opening, hydrolyzation	10 s	No	turn-on	0.377 nM (fluorescence) 4.54 nM (UV)	1.0–9.0	Tap water, drinking water, lake water, strawberry, watermelon, cherry, rice, lentil, green lentils, beans, corn, A549 cells, H1299 cells	[120]
** QR **	PBS/DMSO = 1/1, *v*/*v*	630/410	725/465	Hg^2+^-induced spirolactam ring opening	1 min	No	ratiometric	23 nM	5.0–8.0	Tap water, HeLa cells	[121]
** Se-SiRH **	MeOH/PBS= 1/1, *v*/*v*	616	672	Hg^2+^-induced spirolactam ring opening	40 min	-	turn-on	2.1 nM	3.0–9.0	Tap water, river water, HepG2 cells	[122]
** CyP **	CH_3_OH/PBS = 3/7, *v*/*v*	680	710	ICT	<10 min	-	turn-on	54.8 nM	7.0–8.0	SHSY-5Y cells, zebrafish, mice	[123]
** CyHg-Cl **	PBS/DMSO = 4/1, *v*/*v*	680	725	ICT	2.5 min	No	turn-on	0.59 µM	5.0–9.0	Tap water, Yangtze river water, distilled water, HeLa cells	[124]
** HCYS **	PBS	615	674	ICT	<1 min	Yes	turn-off	1.41 mM	-	HepG2 cells, HeLa cells, zebrafish, pea spouts, lake water, tap water, ultrapure water	[125]
** HDS-Hg **	DMSO/PBS = 1/4, *v*/*v*	700	765	ICT	3 min	No	turn-on	89 nM	-	Environmental water samples, paeonia lactiflora, panax quinquefolius glycyrrhiza uralensis, seafood samples, tobacco seedling leaves, HepG2 cells, THLE-2 cells, A549 cells, BRL-3A cells, mice	[126]
** HTC-S **	DMSO/PBS = 1/1, *v*/*v*	500/740	625/816	ICT	40 s	No	ratiometric	0.10 µM	-	Sandy soil, plant soil, carrot, radish, lettuce, rice, mung beans, shrimp, tap water, sewage water, distillate water, HeLa cells	[127]
** CB-Hg **	EtOH/HEPES = 1/1, *v*/*v*	670/430	720/495	CHEF	30 s	No	ratiometric	6.94 nM	2.0–10.0	Tap water, mineral water, rice, HeLa cells, zebrafish	[128]
** DCM-Hg **	DMSO/PBS= 5/5, *v*/*v*	550	695	ICT	25 min	No	turn-on	3.35 µM	5.0–9.0	HepG2 cells	[129]
** TM-ND-Hg **	DMSO/PBS = 7/3, *v*/*v*	611/436	794/516	ICT	<1 min	No	ratiometric	0.39 mM	6.0–9.0	A549 cells, zebrafish, mineral Water, tap water, lake water	[130]
** TP-Hg **	DMSO/PBS = 7/3, *v*/*v*	563	770	ICT	<1 min	No	turn-on	0.13 mM	6.0–9.0	A549 cells, zebrafish	[131]
** BBN-Hg **	DMSO:PBS = 3/7, *v*/*v*	560	650	ICT	-	No	turn-on	53 nM	5.8–8.0	Tap water, Chao lake water, tea water, scutellaria baicalensis water, loess soil, green soil, purple soil, sandy soil, MCF-7 cells, zebrafish, roots of rice and Salvia miltiorrhiza	[132]

Note: Probe names are shown in bold.

**Table 3:** The photophysical properties of representative NIR fluorescent probes for Zn^2+^.

Name	Solvent	Ex (nm)	Em (nm)	Sensing Mechanism	Response Time	Reversible	Signal Transduction Mode	Detection Limit	pH Range	Application	Ref.
** Probe 2 **	(EtOH + ACN)/ HEPES = 1/1, *v*/*v*	520	670	PET	1 min	Yes	turn-on	2.7 nM	6.0–7.0	Pond water	[136]
** ISOHP **	-	-	668	PET, isomerization	15 s	-	turn-on	0.21 mM	6.0–8.0	Drinking water, A549 cells	[137]
** NIR-Zn **	CH_3_CN/HEPES = 1/1, *v*/*v*	481	660	PET	30 s	Yes	turn-on	4.8 nM	-	Pond water, A549 cells	[138]
** AXS **	DMSO/H_2_O = 7/3, *v*/*v*	508	688.4	PET	-	Yes	turn-on	0.069 nM	-	Cauliflower, crispy pear, Chinese cabbage, garlic, Chinese date, lemon, Zinc gluconate oral liquid, C. elegans, mice	[139]
** IQ **	H_2_O/EtOH = 1/9, *v*/*v*	495	664	PET	30 s	Yes	turn-on	60.5 nM	6.0–8.0	Lake water, tap water, distilled water, rice, photo, sandy soil, plant soil, HeLa cells	[140]
** BTDM **	DMSO/H_2_O = 8/2, *v*/*v*	535	683	IET	250 s	Yes	turn-on	18 nM	-	Apple, orange, banana, garlic pear, C. elegans, mice,	[141]
** Probe 1 **	HEPES/DMF = 1/1, *v*/*v*	482	653	PET	<20 s	-	turn-on	15.3 nM	5.0–11.0	Pond water, river water, HepG 2 cells	[142]
** NIRF **	DMSO/HEPES = 2/3, *v*/*v*	484	658	CHEF	20 s	Yes	turn-on	0.79 mM	4.0–11.0	Distilled water, tap water, lake water, sprouted potato, RAW264.7 cells, mice	[143]
** DR6G **	DMF/TRis-HCl = 1/1, *v*/*v*	483	646	FRET	within a few seconds,	Yes	turn-on	20.2 nM	7.0–11.0	Zinc gluconate, calcium zinc gluconate, HUVEC cells, mice	[144]
** DHP **	THF/HEPES = 1/1, *v*/*v*	428	660	CHEF, ESIPT, ICT	4.7 s	Yes	turn-on	0.13 mM	-	MCF-7 cells, HepG2 cells, mung bean sprout	[145]
** TZn **	HEPES/EtOH = 9/1, *v*/*v*	580	652	PET	70 s	Yes	turn-on	0.31 mM	-	Milk, water, red wine, apple juice, HeLa cells	[146]
** Mito-Zn **	HEPES/ACN = 99/1, *v*/*v*	684/502	807/653	PET	-	-	ratiometric	26.7 nM	-	HeLa cells, mice	[147]

Note: Probe names are shown in bold.

**Table 4:** The photophysical properties of representative NIR fluorescent probes for Pb^2+^.

Name	Solvent	Ex (nm)	Em (nm)	Sensing Mechanism	Response Time	Reversible	Signal Transduction Mode	Detection Limit	pH Range	Application	Ref.
** NDHN **	DMSO/H_2_O = 3/7, *v*/*v*	416 nm	667/584	ICT, PET, C=N isomerization	-	-	ratiometric	0.157 mM	-	Milk, milk powder, white wine, soybean, black bean, broad bean, HeLa cells, Caenorhabditis elegans, mice	[150]
** DHB **	DMF/HEPES = 4/6, *v*/*v*	430	650	ICT	-	Yes	turn-on	32.3 nM	6.0–7.0	Tap water, Songhua River water, soil extract, aqueous solution of licorice tablets extract, canned cola, HeLa cells, zebrafish, mice, rice roots	[151]
** LCE1 **	Tris-HCl/DMF = 9/1, *v*/*v*	488	670	ICT	30 s	-	turn-off	0.34 mM	4.5–10.0	Tap water, river water, lake water, black tea, milk, Chrysopogon zizanioides (L.) Roberty, Medicago sativa L., MCF-7 cells, mice	[152]
** YSQ **	MeOH/H_2_O= 9/1, *v*/*v*	490	678	ICT, PET, C=N isomerization	<1 min	-	turn-on	18 nM	-	Lake water, tap water, PK-15 cells	[153]

Note: Probe names are shown in bold.

**Table 5:** The photophysical properties of representative NIR fluorescent probes for Fe^2+^/Fe^3+^.

Name	Solvent	Ex (nm)	Em (nm)	Sensing Mechanism	Response Time	Reversible	Signal Transduction Mode	Detection Limit	Ph Range	Application	Ref.
** NRF-Fe2+ **	Tris-HCl/ DMSO = 7/3, *v*/*v*	430	460/675	FRET	<30 min	No	ratiometric	75 nM	2.0–8.0	A549 cells, mice	[162]
** MDJ-O **	PBS	535	720	PET	5 min	No	turn-on	3.09 mM	4.0–9.0	HepG2 cells, zebrafish, mice	[163]
**DICO-Fe(II)**	HEPES/EtOH = 9/1, *v*/*v*	530	666	ICT	<8 min	No	turn-on	47 nM	-	Spinach, rape, bean sprouts, chicken samples, HeLa cells, zebrafish, mice	[164]
**DCA-MIn**	MeOH/H_2_O = 5/5, *v*/*v*	570	670	ICT, PET	<15 s	-	turn-on	19.5 mM	4.0–8.0	Tap water, lake water, MCF-7 cells, anti-counterfeiting ink	[165]
**DHX-Fe**	PBS/EtOH = 7/3, *v*/*v*	610	690	ICT	<5 min	No	turn-on	115 nM	-	A2780 cells, mice	[166]
**BDNO**	DMSO/H_2_O = 1/1, *v*/*v*	610/ 540	715/570	ICT	<5 s	No	ratiometric	41 nM	4.0–10.0	Real water samples, apple juice, orange juice, wine, tap water, lake water, sea water, mung bean sprouts	[167]
**FTP**	EtOH/HEPES = 3/7, *v*/*v*	690	743	PET	<2 min	No	turn-on	24.0 nM (fluorescence) 79.7 nm (UV)	4.0–10.0	Tap water Wolong Lake water Tianyi Lake water, HepG2 cells, mice	[168]
**CyLIP**	HEPES	660	705	ICT	20 min	No	turn-on	51 nM	5.0–9.0	HepG2 cells, mice	[169]
**QM-FV**	HEPES/DMSO = 1/1, *v*/*v*	450	740	ICT	<150 s	No	turn-on	25 nM	-	HepG2 cells, HeLa cells, Huh7 cells, AML12 cells, HEK293T cells, Vero cells, mice	[170]

Note: Probe names are shown in bold.

## Data Availability

No new data were created or analyzed in this study. Data sharing is not applicable to this article.

## References

[1] Wu X.Y., Cobbina S.J., Mao G.H., Xu H., Zhang Z., Yang L.Q. (2016). A review of toxicity and mechanisms of individual and mixtures of heavy metals in the environment. Environ. Sci. Pollut. Res..

[2] Ciupa A. (2025). Hydrazone fluorescent sensors for the monitoring of toxic metals involved in human health from 2014–2024. RSC Adv..

[3] Tardito S., Bassanetti I., Bignardi C., Elviri L., Tegoni M., Mucchino C., Bussolati O., Franchi-Gazzola R., Marchiò L. (2011). Copper binding agents acting as copper ionophores lead to caspase inhibition and paraptotic cell death in human cancer cells. J. Am. Chem. Soc..

[4] Qing Z.H., Mao Z.G., Qing T.P., He X.X., Zou Z., He D.G., Shi H., Huang J., Liu J.B., Wang K.M. (2014). Visual and portable strategy for copper(II) detection based on a striplike poly(thymine)-caged and microwell-printed hydrogel. Anal. Chem..

[5] Zhang W., Zhang X., Tian Y.L., Zhu Y., Tong Y.D., Li Y., Wang X.J. (2018). Risk assessment of total mercury and methylmercury in aquatic products from offshore farms in China. J. Hazard Mater..

[6] Li Y.B., Yuan M., Khana A.J., Wang L.P., Zhang F. (2019). Peptide-gold nanocluster synthesis and intracellular Hg^2+^ sensing. Colloids Surf. A Physicochem. Eng. Asp..

[7] Fukada T., Yamasaki S., Nishida K., Murakami M., Hirano T. (2011). Zinc homeostasis and signaling in health and diseases. J. Biol. Inorg. Chem..

[8] Liu H.L., Cui S.Q., Shi F., Pu S.Z. (2019). A diarylethene based multifunctional sensor for fluorescent detection of Cd^2+^ and colorimetric detection of Cu^2+^. Dyes Pigm..

[9] Jiang C.Y., Yang L., Li P., Liu Y.T., Li S.J., Fu Y., Ye F. (2021). A simple and rapid fluorescent approach for Pb^2+^ determination and application in water samples and living cells. Spectrochim. Acta A Mol. Biomol. Spectrosc..

[10] Xie Y., Hou W., Song X., Yu Y., Huang J., Sun X., Kang R., Tang D. (2016). Ferroptosis: Process and function. Cell Death Differ..

[11] Toyokuni S. (2009). Role of iron in carcinogenesis: Cancer as a ferrotoxic disease. Cancer Sci..

[12] Kumar M., Puri A. (2012). A review of permissible limits of drinking water. Indian J. Occup. Environ. Med..

[13] Berhanu A.L., Mohiuddin I., Malik A.K., Aulakh J.S., Kumar V., Kim K.H. (2019). A review of the applications of Schiff bases as optical chemical sensors. TrAC Trends Anal. Chem..

[14] Singh H., Bamrah A., Bhardwaj S.K., Deep A., Khatri M., Kim K.H., Bhardwaj N. (2021). Nanomaterial-based fluorescent sensors for the detection of lead ions. J. Hazard. Mater..

[15] Erdemir E., Suna G., Gunduz S., Şahin M., Eğlence-Bakır S., Karakuş E. (2022). Rapid, ultrasensitive, highly selective detection of toxic Hg (II) ions in seabass, swordfish and water samples. Food Chem..

[16] Si L., Wu Q., Jin Y., Wang Z. (2024). Research progress in the detection of trace heavy metal ions in food samples. Front. Chem..

[17] Li K., Yang H., Yuan X., Zhang M. (2021). Recent developments of heavy metals detection in traditional chinese medicine by atomic spectrometry. Microchem. J..

[18] Ustabasi G.S., Ozcan M., Yilmaz I. (2021). Review-voltammetric determination of heavy metals with carbon-based electrodes. J. Electrochem. Soc..

[19] Malik L.A., Bashir A., Qureashi A., Pandith A.H. (2019). Detection and removal of heavy metal ions: A review. Environ. Chem. Lett..

[20] Yu L.H., Pang Y.H., Mo Z.L., Huang Y.Y., Shen X.F. (2021). Coordination array for accurate colorimetric sensing of multiple heavy metal ions. Talanta.

[21] Yao S.-Y., Cui H.-W., Jiang Z.-B., Wang H., Li J.-Y., Wang Y.-Y., Shen F.-Q., Zhao L.-X., Liu Y.-T., Zou Y.-L. (2025). A NIR isophorone-based Zinc coordination fluorescence probe for the detection of glyphosate and application in environment, food and living systems. J. Fluoresc..

[22] Dai M.C., Yang Y.J., Sarkara S., Ahn K.H. (2023). Strategies to convert organic fluorophores into red/near-infrared emitting analogues and their utilization in bioimaging probes. Chem. Soc. Rev..

[23] Li G.F., Ge E.X., Zheng H., Lin W.Y. (2025). Advancements and perspectives on organelle-targeted fluorescent probes for super-resolution SIM imaging. Chem. Sci..

[24] Ye Y.X., Pan J.-C., Wang H.-C., Zhang X.-T., Zhu H.-L., Liu X.-H. (2024). Advances in small-molecule fluorescent probes for the study of apoptosis. Chem. Soc. Rev..

[25] He J.R., Han X.W., Yue Y.K. (2025). Recent progress of dual-responsive fluorescent probes for polarity and analytes. Analyst.

[26] Wang S., Guo W., Wan B.-W., Wang K.-M., Dong B.L., Jiang C.-S., Zhang J. (2026). Recent advances in Golgi-targeted fluorescent probes in ferroptosis research. Analyst.

[27] He L., Li H., Tang Y., Ren T.-B., Yuan L. (2024). Recent advances in fluorescent probes for fatty liver imaging by detecting lipid droplets. J. Mater. Chem. B.

[28] Wang K.Y., Zhang Z.X., Guo X.F., Zhang G.Y., Jin Y.L., Wang Z. (2025). Recent advances in organic molecule fluorescent probes for microbial imaging. J. Mater. Chem. B.

[29] Wang J.D., Yin C.X., Huo F.J. (2025). Recent advances in glutathione fluorescent probes based on small organic molecules and their bioimaging. Analyst.

[30] Xiao W.W., Chen S., Liu M.X., Yu Y.L. (2025). Fluorescent probes for lysosomes, mitochondria, and lipid droplets: Precision design, dynamic microenvironment monitoring, and heterogeneity exploration. Chem. Commun..

[31] Geng Y.J., Wang Z., Zhou J.Y., Zhu M.G., Liu J., James T.D. (2023). Recent progress in the development of fluorescent probes for imaging pathological oxidative stress. Chem. Soc. Rev..

[32] Zhang J.J., Zhao J.Y., Lu H.H., Sayman H.B., An F.F., Ting R., Qu W. (2026). Turn-on near-infrared fluorescence/positron emission tomography dual-modal probe for intracranial hemorrhage diagnosis. J. Mater. Chem. B.

[33] Murugan P., Praburaman L., Singaravel V., Moovendhan M., Peng Y.-L., Yang F., Liu S.-Y. (2025). A comprehensive review on recent developments in near-infrared organic fluorophores for breast cancer: From molecular design to diagnostic applications. J. Mater. Chem. B.

[34] Su L.N., Wang J.D., Liu B.W., Liu H., Chen Q.X., Liu J., Li S.L., Yuan L., An L.H., Lin H. (2024). Construction of a near-infrared fluorescent probe for dynamic monitoring and early diagnosis of heart failure. ACS Sens..

[35] Biswas B., Surbhi Dogra S., Sen A., Murugan N.A., Dhingra P., Jaswal K., Mondal P., Ghosh S. (2024). NIR-I emissive cyanine derived molecular probe for selective monitoring of hepatic albumin levels during hyperglycemia. J. Mater. Chem. B.

[36] Biswas B., Dogra S., Dey G., Murugan N.A., Mondal P., Ghosh S. (2022). Near-infrared emissive cyanine probes for selective visualization of the physiological and pathophysiological modulation of albumin levels. J. Mater. Chem. B.

[37] Li Y.J., Jiang M.L., Yan M.M., Ye J.T., Li Y., Dehaen W., Yin S.C. (2024). Near-infrared boron-dipyrrin (BODIPY) nanomaterials: Molecular design and anti-tumor therapeutics. Coord. Chem. Rev..

[38] Liang Y.Y., Xu T.T., Xu S., Xu X., Wang L.P., Wang Z.L., Wang S.F. (2025). A HClO-activated BODIPY based ratiometric fluorescent probe with dual near-infrared channels for differentiating cancerous cells from normal cells and surgical guidance of tumor resection. Biosens. Bioelectron..

[39] Zhao J.Y., Ren L.-Q., Lan D.R., Zhan B.Q., Zhang J.Y., Mu J., He C. (2025). Streamlined construction of boron-stereogenic BODIPY library for near-infrared bioimaging. Nat. Commun..

[40] Mei Y., Hai Z.J., Li Z.Y., Rong K.R., Tang W.J., Song Q.-H. (2024). Dual-responsive near-infrared BODIPY-based fluorescent probe for the detection of F- and HClO in organisms. Anal. Chem..

[41] Sun Y., Zhang Y.X., Zhou C.L., Yan L.Q. (2025). Research progress on design strategies of hemicyanine-based fluorescent probes and their applications in environmental and food analysis. Analyst.

[42] Liu J., Zhu M.-S., Zhang G., Sun R., Xu Y.-J., Ge J.-F. (2023). Viscosity probes towards different organelles with red emission based on an identical hemicyanine structure. Analyst.

[43] Sun Q.L., He D.M., Zhang L.S., Li Z.H., Qu L.B., Sun Y.Q. (2023). 2023 Coumarin-hemicyanine-based far-red to near-infrared fluorescent probes: A new generation of fluorescent probe design platform. TrAC Trends Anal. Chem..

[44] Liu Q.N., Li Z.Y., Huang Y.J., Lin Z.N., Xing-Can Shen X.-C., Chen H. (2025). Deep-NIR to NIR-II hemicyanine fluorophore scaffolds with dual optically tunable sites for in vivo multiplexed imaging. Chem. Sci..

[45] Xuan J.G., Yu J.J., Huang C.S. (2024). Research progress of cyanine-based near-infrared fluorescent probes for biological application. ChemBioChem.

[46] Kong F., Liu H.Q., Huang J., Qin J.C. (2024). Imaging of ONOO^-^ fluctuations during drug-induced liver/kidney injury in vitro and in vivo via a dicyanoisophorone-based NIR fluorescent probe with a large Stokes shift. J. Mater. Chem. B.

[47] Zhang L., Yao X., Peng M., Ma Y., Peng Y., Wang Y.-W. (2025). An HPQ-based near-infrared dye for the detection of ClO- and accurate imaging of cells and mouse tumor sites in situ. Org. Biomol. Chem..

[48] Wang X., Wang N.W., Ren Y.K., Wang J.C., Bai J., Hua H.M., Li D.H. (2025). A near-infrared fluorescent probe selectively recognizing cysteine to release H2S and its applications. Analyst.

[49] Lin B., Li S.T., Han Y.F. (2026). The dicyanoisophorone scaffold: A versatile platform for near-infrared, large stokes shift fluorescent probes in sensing and bioimaging. J. Mol. Struct..

[50] Li H.D., Wang J.Y., Kim H., Peng X.J. (2024). Activatable near-infrared versatile fluorescent and chemiluminescent dyes based on the dicyanomethylene-4H-pyran scaffold: From design to imaging and theranostics. Angew. Chem. Int. Ed..

[51] Liu Q., Sun C.Y., Dai R.L., Yan C.X., Zhang Y.T., Zhu W.-H., Guo Z.Q. (2024). Engineering high-performance dicyanomethylene-4H-pyran fluorophores for biosensing and phototherapy. Coord. Chem. Rev..

[52] Zhou Z.L., Fang C., Yu F.J., Shen Y.M., Xu H., Li H.T., Zhang Y.Y. (2024). Visualization of cysteine in AD mouse with a high-quantum yield NIR fluorescent probe. Talanta.

[53] Ohno H., Sasaki E., Yamada S., Hanaoka K. (2024). Recent advances in Si-rhodamine-based fluorescent probes for live-cell imaging. Org. Biomol. Chem..

[54] Zeng S., Liu X.S., Kafuti Y.S., Kim H., Wang J.Y., Peng X.J., Li H.D., Yoon J. (2023). Fluorescent dyes based on rhodamine derivatives for bioimaging and therapeutics: Recent progress, challenges, and prospects. Chem. Soc. Rev..

[55] Jin Y.L., Liu R., Zhan Z.X., Lv Y. (2019). Fast response near-infrared fluorescent probe for hydrogen sulfide in natural waters. Talanta.

[56] Lu W.Y., Li H.-J., Wu Y.-C. (2024). Assessment of environmental and biological stress using mitochondria-targeted red-emitting and near-infrared fluorescent probes for biothiol analysis: A review. Environ. Chem. Lett..

[57] Jeong H., Wu X.F., Lee J.-S., Yoon J. (2023). Recent advances in enzyme-activated NIR fluorescent probes for biological applications. TRAC Trends. Anal. Chem..

[58] Zhao J.T., Ma T., Chang B.B., Fang J.G. (2022). Recent progress on NIR fluorescent probes for enzymes. Molecules.

[59] Reja S.I., Minoshima M., Hori Y., Kikuchi K. (2022). Near-infrared fluorescent probes: A next generation tool for protein-labeling applications. Chem. Sci..

[60] Wang J., Sheng Z.J., Guo J.R., Wang H.-Y., Sun X.L., Liu Y. (2023). Near-infrared fluorescence probes for monitoring and diagnosing nephron-urological diseases. Coord. Chem. Rev..

[61] Li Y., Zhuang D.P., Wang J.J., Huang H.Y., Li R.C., Wu C.Y., Deng Y.F., Hu G.W., Guo B. (2022). Recent advances in small molecular near-infrared fluorescence probes for a targeted diagnosis of the Alzheimer disease. Analyst.

[62] Bajad N.G., Kumar A., Dingh S.K. (2023). Recent advances in the development of near-infrared fluorescent probes for the in Vivo brain imaging of Amyloid-β species in Alzheimer’s disease. ACS Chem. Neurosci..

[63] Nandanwar S., Nguyen V.K.T., Tran D.L., Pham T.C., Lee S. (2026). Small organic molecule-based next-generation NIR fluorescent probes for cancer theranostics. Coord. Chem. Rev..

[64] Ye J.S., Wang D.Y., Wang Z.Y., Cao M.Y., Shen Y.Q., Yu B., Cong H.L. (2025). Recent advances in NIR small molecule activable probes for imaging and therapy. Coord. Chem. Rev..

[65] Liu Y., Sun Y.N., Zhao P., Liu Z.L., Wang Y.L., Wang Y.L., Zhao R.T., Wang C.N., Wang S.H. (2025). Near-infrared fluorescent probe for the early diagnosis of Alzheimer’s disease. Med. Res. Rev..

[66] Yan L.Q., Tang L.T., Wu X.Z., Li L. (2024). Recent advances in organic small-molecule fluorescent probes based on dicyanoisophorone derivatives. Crit. Rev. Anal. Chem..

[67] Zhao M., Guo Y.-S., Xu W.-N., Zhao Y.-F., Xie H.-Y., Li H.-J., Chen X.-F., Zhao R.-S., Guo D.-S. (2020). Far-red to near-infrared fluorescent probes based on silicon-substituted xanthene dyes for sensing and imaging. TRAC Trends. Anal. Chem..

[68] Feng L., Chen W.J., Ma X.X., Liu S.H., Yin J. (2020). Near-infrared heptamethine cyanines (Cy7): From structure, property to application. Org. Biomol. Chem..

[69] Li M., Li Y.T., Wang X.J., Cui X.Y., Wang T. (2019). Synthesis and application of near-infrared substituted rhodamines. Chin. Chem. Lett..

[70] Li H.D., Kim H., Xu F., Han J.J., Wang J.Y., Pu K.Y., Peng X.J., Yoon J. (2022). Activity-based NIR fluorescent probes based on the versatile hemicyanine scaffold: Design strategy, biomedical applications, and outlook. Chem. Soc. Rev..

[71] Zeng Z.L., Liew S.S., Wei X., Pu K.Y. (2021). Hemicyanine-based near-infrared activatable probes for imaging and diagnosis of diseases. Angew. Chem. Int. Ed..

[72] Muhammad S., Ahmad H., Yan Y.Q., Chen X., Muhammad S., Maridevaru M.C., Roy S., Wang Z., Zhang Y.H., Guo B. (2025). Hemicyanine-based fluorescent probes: Advancements in biomedical sensing and activity-based detection. Coord. Chem. Rev..

[73] Routray S., Acharya S., Nayak L., Pattnaik S., Satapathy R. (2025). Advances in mercury ion sensing using BODIPY based compounds: A sexennial update. RSC Adv..

[74] Bumagina N.A., Antina E.V. (2024). Review of advances in development of fluorescent BODIPY probes (chemosensors and chemodosimeters) for cation recognition. Coord. Chem. Rev..

[75] Zhao X., Li X., Zhang H.B., Chen X., Xu J., Yang J., Zhang H.C., Hu G.Z. (2022). Atomic-dispersed copper simultaneously achieve high-efficiency removal and high-value-added conversion to ammonia of nitrate in sewage. J. Hazard. Mater..

[76] Jia T., Fu M., Zhang M., Qiu J., Zhu H., Gao Y. (2022). A novel cholesterol conjugated fluorescence probe for Cu^2+^ detection and bioimaging in living cells. Spectrochim. Acta A Mol. Biomol. Spectrosc..

[77] Qiu S.Y., Wei Y.H., Tu T.H., Xiang J.J., Zhang D.W., Chen Q.L., Luo L.G., Lin Z.Y. (2020). Triazole-stabilized fluorescence sensor for highly selective detection of copper in tea and animal feed. Food Chem..

[78] Zhou Z.L., Chen S.Y., Huang Y.H., Gu B., Li J.Q., Wu C.Y., Yin P., Zhang Y.Y., Li H.T. (2022). Simultaneous visualization and quantification of copper (II) ions in Alzheimer’s disease by a near-infrared fluorescence probe. Biosens. Bioelectron..

[79] Zhang Y.B., Miu J., Wang B.L., Rong X.Q., Liu J., Tang C., Wang C., Gao W.X., Gui Y.R., Wang H.P. (2023). A novel near-infrared fluorescent probe based on the dicyanoisophorone for the selective detection of Cu^2+^ in real water samples. J. Mol. Struct..

[80] Shi Y., Yu J.R., Song Y.X., Fan J., Wang X.W., Li S.J., Li H.Q. (2025). Multifunctional near-infrared fluorescent probe for sensing of lysine and Cu^2+^/Fe^3+^ and relay detection of biothiols. Talanta.

[81] Dong Y.J., Qiao R., Song Q.X., Chen M.Y., Wang S.Z., Hu J.D., Wang X., Xue W.H., Zhang Y.Q., Bai C.B. (2025). Two birds with one stone: A near-infrared AIE fluorescent probe for highly selective recognition of Cu^2+^/GSH and bioimaging. J. Photochem. Photobiol. A Chem..

[82] Li P.J., Yang J.J., Yan L.Q. (2025). A near-infrared fluorescent probe for detecting Cu^2+^ and its versatile applications. Anal. Methods.

[83] Zhang Y.Y., Zhai H.S., Shang Z.Y., Wang Y.S., He M.M., Zhang Z.Q., Zhang R., Meng Q.T. (2025). Near-infrared copper ions fluorescent probe for assessing Alzheimer’s disease progression and its application in real samples by constructing a portable test kit. Sens. Actuators B Chem..

[84] Gao G.Q., Hao C.G., Ding C.X., Zheng X., Liu L.J., Xie P.H., Guo F.Q. (2023). A near-infrared colorimetric and fluorometric chemodosimeter for Cu^2+^ based on a bis-spirocyclic rhodamine and its application in imagings. Res. Chem. Intermediat..

[85] Wu J.-P., Xing A.-P., Yuan Y.-Y., Hao Y.-T., Pan P., He S.-N., Yuan J., Zeng D. (2024). A near-infrared hemicyanine-based colorimetric and fluorescent chemosensor for highly sensitive detection of Cu^2+^ and imaging in living cells and in vivo. Dyes Pigm..

[86] Yuan Y.-Y., Hao Y.-T., Zeng D., Pan P., Lu J.-X., Zhang B., He S.-N., Xing A.-P., Chen S.-Q., Yuan J. (2024). A near-infrared fluorescent probe for the detection of Cu^2+^ in Chinese herbal medicine and imaging in living cells. Spectrochim. Acta A Mol. Biomol. Spectrosc..

[87] Zhu Y.-J., Pan P., Zhang B., Lu J.-X., Wang J., Shen A., Li X.-M., Xing A.-P., Zeng D., Yuan J. (2025). A highly sensitive near-infrared fluorescent probe for intelligent visual detection of Cu^2+^ in water/vegetables and bioimaging in vitro and in vivo. Dyes Pigm..

[88] Zhang B., Zeng D., Zhang Y.-X., Pan P., Wang J., Shen A., Lu J.-X., Zhu Y.-J., Xing A.-P., Yuan J. (2025). A highly sensitive and selective rectilinearly π-extended NIR fluorescent rhodamine probe for Cu^2+^ detection in real food samples and fluorescence bioimaging in living cells and mice. Spectrochim. Acta A Mol. Biomol. Spectrosc..

[89] Wang B.B., Shi J.Z., Zhai W.B., Jiang L., Ma Y.M., Zhang Z.K., Zhao F.Q., Wu X.T., Wu J., Wang J.F. (2025). Construction of a bifunctional near-infrared fluorescent probe for visualization of copper (II) ions and amyloid-β aggregates in Alzheimer’s disease. Sens. Actuators B Chem..

[90] Guo T.Y., Tian R.W., Qu W.B., Yang B., Geng Z.R., Wang Z.L. (2022). A near-infrared turn-on fluorescent sensor for the determination of copper in mitochondria. Dyes Pigm..

[91] Aydin Z., Yan B., Guo M.L. (2026). A heptamethine cyanine-based near-infrared optical sensor for copper(II) detection in aqueous solutions and living cells. Sensors.

[92] Zhu J.P., Graziotto M.E., Cottam V., Hawtrey T., Adair L.D., Trist B.G., Pham N.T.H., Rouaen J.R.C., Ohno C., Heisler M. (2024). Near-infrared ratiometric fluorescent probe for detecting endogenous Cu^2+^ in the brain. ACS Sens..

[93] Chang Z.J., Li S.M., Ye J.-H., Lin F.Y., Chen Y.C., Guo Z.J., He W.J. (2025). A dual-response ratiometric near-infrared fluorescence probe based on cyanine platform for Cu^2+^ detection and its imaging in vitro and vivo. Spectrochim. Acta A Mol. Biomol. Spectrosc..

[94] Zhang T., Chi H.R., Guo J.J., Lu X.M., Li G.L. (2025). Construction of a Cu^2+^-responsive NIR fluorescent probe and the preliminary evaluation of its multifunctional application. J. Fluoresc..

[95] Zhou F.J., Xu Q.R., Li R.X., Leng L.H., Yang M.F., Zhuang X.Y., Yang H., Yang F., Wen X., Zou M.J. (2026). A reaction-activated near-infrared hemicyanine probe for tandem detection of copper(II) and glutathione in food systems and living cells. Microchem. J..

[96] Chen J.M., Luo R.Q., Li S., Shao J.P., Wang T., Xie S.M., Xu L., You Q.Y., Feng S.M., Feng G.Q. (2024). A novel NIR fluorescent probe for copper(II) imaging in Parkinson’s disease mouse brain. Chem. Sci..

[97] Zhou H., Wang S., Jin Y., Pang X.-F., Zhao Q., Zhang T., Zhang J. (2025). A near-infrared “turn-on” fluorescent probe for selective detection of copper(II) ions in aqueous media and its application in cell imaging. Spectrochim. Acta A Mol. Biomol. Spectrosc..

[98] Yi M.R., Zhao J.T., Shen R.P., Ma T., Tu J.W., Li X.M., Zhang B.X., Fang J.G. (2025). A near-infrared fluorescent probe for sensing cuprous ions. Sens. Actuators B Chem..

[99] Al-Sulaiti M.M., Soubra L., Al-Ghouti M.A. (2022). The causes and effects of mercury and methylmercury contamination in the marine environment: A review. Curr. Pollut. Rep..

[100] Yin P., Zou T., Yao G., Li S., He Y., Li G., Li D., Tan W., Yang M. (2023). In situ microwave-assisted preparation of NS-codoped carbon dots stabilized silver nanoparticles as an off-on fluorescent probe for trace Hg^2+^ detection. Chemosphere.

[101] Piyanuch P., Aryamueang S., Khrootkaew T., Mahingsadet K., Chansaenpak K., Kamkaew A. (2024). Development of a new series of thioacetal based fluorescence chemosensors for highly sensitive determination of Hg^2+^ in environmental samples and cell imaging. Spectrochim. Acta A.

[102] Zhang Y.B., Yan Q., Cheng Y.T., Wang B.L., Rong X.Q., Kuang Y.H., Qiu X.Y., Sun L., Zhou Y.Y. (2022). A novel “Off–On” NIR fluorescent probe for detecting Hg^2+^ based on dicyanoisophorone and its application in bio-imaging and real water samples. Food Anal. Method..

[103] Jiang L., Zheng T., Xu Z.X., Li J.Y., Li H.Q., Tang J.J., Liu S.C., Wang Y.Y. (2022). New NIR spectroscopic probe with a large Stokes shift for Hg^2+^ and Ag^+^ detection and living cells imaging. Spectrochim. Acta A Mol. Biomol. Spectrosc..

[104] Min Y., Han X., Qi Y., Jiang L., Song Y., Ma Y., Zhang J., Li H. (2024). A novel near-infrared fluorescent and colorimetric probe for selective detection of Ag^+^ and Hg^2+^. Color. Technol..

[105] Ma M.S., Wang S.S., Yu S.H., Zeng X.D., Liu Z.G. (2025). A highly selective NIR fluorescent probe for Hg^2+^ and its multiple application in biological and environmental systems. J. Mol. Struct..

[106] Fang Y.K., Zhang Y., Liu C.Y., Zhu H.C., Wang K., Ma L.X., Li X.K., Rong X.D., Li W.Z., Sheng W.L. (2025). Development of a novel ICT-ESIPT-based NIR ratiometric fluorescent probe for specific detection of Hg^2+^ in the environment and living organisms. Spectrochim. Acta A Mol. Biomol. Spectrosc..

[107] Pei S.-C., Li C.-Z., Pei X.-Y., Zeng W.-H., Zhang Y.-Y., Jiaing K.-X., Huang X., Liao X.-L., Chen J. (2023). Dual colorimetric and near-infrared fluorescence probe for Hg^2+^ detection and cell imaging. Spectrochim. Acta A Mol. Biomol. Spectrosc..

[108] Wang X., Wang X.C., Han Q.X. (2023). Intelligent detection strategy and bioimaging application of dual-responsive Hg^2+^ and ONOO-using near-infrared probes. Anal. Chim. Acta.

[109] Erdemir S., Oguz M., Malkondu S. (2022). A NIR fluorescent sensor based on thiazoline-isophorone with low cytotoxicity in living cells for Hg^2+^ detection through ICT associated hydrogen bonding effect. Anal. Chim. Acta.

[110] Xie F.L., Yang H., Lu D.Q., Wu X.Z., Yan L.Q. (2024). A dicyanoisoflurone-based near-infrared fluorescence probe for highly sensitive detection of Hg^2+^. J. Fluoresc..

[111] Wu L.Y., Wang X., Huang J., Wei H.Y., Kan C. (2022). Switch-type near-infrared fluorescent probes for Hg^2+^ based on rhodamines. J. Photochem. Photobiol. A Chem..

[112] Huang J., Liu K.Y., Tian J.X., Wei H.Y., Kan C. (2024). A rhodamine NIR probe for naked eye detection of mercury ions and its application. Spectrochim. Acta A Mol. Biomol. Spectrosc..

[113] Liang F.H., Xu L.B., Jin D.H., Dong L.D., Lin S.R., Huang R., Song D.Q., Ma P.Y. (2022). A novel near-infrared fluorescence probe for detecting and imaging Hg^2+^ in living cells. Luminescence.

[114] Patthana P., Zhong H.-C., Wu Q., Ren T.-B., Yuan L. (2024). Engineering a far-red fluorescent probe for rapid detection of Hg(II) ions in both cells and zebrafish. Spectrochim. Acta A Mol. Biomol. Spectrosc..

[115] Zhou J.J., Li J.C., Xu X.T., Long S., Cui N.N., Zhang Y., Shi L.H., Zhou J. (2024). Imaging gastrointestinal damage due to acute mercury poisoning using a mitochondria-targeted dual near-infrared fluorescent probe. J. Hazard. Mater..

[116] Alcay Y., Ozdemir E., Yildirim M.S., Ertugral U., Yavuz O., Aribuga H., Ozkilic Y., Tuzun N.Ş., Sert A.B.O., Kok F.N. (2023). A methionine biomolecule-modified chromenylium-cyanine fluorescent probe for the analysis of Hg^2+^ in the environment and living cells. Talanta.

[117] Yildirim M.S., Alcay Y., Ozdemir E., Ertugral U., Yavuz O., Aribuga H., Kaya K., Ozkilic Y., Tuzun N.Ş., Sert A.B.O. (2024). Molecular architecture of a novel indoline-fused chromenylium-cyanine probe carrying methionine biomolecule for ultrasensitive analyzing Hg^2+^ ion in real samples. J. Environ. Chem. Eng..

[118] Basan V., Alcay Y., Ozdemir E., Gunduz M.I., Kura B., Tuzun N.Ş., Cil C., Kilic A., Yilmaz I. (2025). Architecture of a novel NIR and switch-on fluorescent chemosensor based on mercapto propanehydrazide-functionalized chromenylium-cyanine for the quantification of mercury(II) in environment and living cells. Spectrochim. Acta A Mol. Biomol. Spectrosc..

[119] Gunduz M.I., Ozdemir E., Alcay Y., Yilmaz E., Yavuz O., Ejder N., Kura B., Tuzun N.Ş., Yilmaz I. (2026). Innovative molecular architecture of a new Hg^2+^/Cu^2+^-dual chemosensor based on functionalizable chromenylium-cyanine framework. Microchem. J..

[120] Cetin D., Ozdemir E., Yavuz O., Alcay Y., Aribuga H., Ertugral U., Ejder N., Yilmaz I. (2026). A novel near-infrared-emitting selenolactone-cyanine chemosensor platform for ultra-trace mercury (II) detection in environmental, food and living cell samples. Talanta.

[121] Lai W.P., Lin Y.F., Ye T.Q., Yu Y.T., Zhou H.W., Li L., Mao G.L., Wang J.B. (2023). A novel near-infrared ratiometric fluorescence probe for Hg^2+^ based on quinoline-fused Rhodamine dye. J. Fluoresc..

[122] Wang X.C., Yu H.F., Tan F. (2025). Selenophene-containing silicon-rhodamine: A novel near-infrared fluorescent probe for Hg^2+^ detection and its application in cell imaging. Spectrochim. Acta A Mol. Biomol. Spectrosc..

[123] Li M., Li R.Y., Chen X.F., Liu J., Shao Y., Xu Z.A., Zhang W. (2022). An activatable near-infrared hemicyanine-based probe for selective detection and imaging of Hg^2+^ in living cells and animals. Analyst.

[124] Zhang Y.B., Ge H.J., Sun L., Cheng Y.T., Xu Z.H., Gao W., Wang B.L., Rong X.Q., Qiu X.Y., Li J.J. (2024). Novel design of near-infrared fluorescent sensors for the detection of Hg^2+^ in living cells and real water samples. Spectrochim. Acta A Mol. Biomol. Spectrosc..

[125] Fang S.J., Zhang L.W., Zhao Y., Zhang X., Zhang L., Chen L.X., Yoon J., Liu S.D. (2024). Insight into mercury ion detection in environmental samples and imaging in living systems by a near-infrared fluorescent probe. Sens. Actuators B Chem..

[126] Liu F.Y., Cui J.W., Hu H.Y., Ren S.M., Chen H.L., Deng Y., Huang Z., Peng C., Dehaen W., Lu J. (2025). Real-time tracking and early diagnosis of mercury(II) exposure via a novel NIR-based fluorescent probe based on thiahemicyanine dye. J. Hazard. Mater..

[127] Erdemir S., Malkondu S., Oguz M., Kocak A. (2024). Monitoring Hg^2+^ ions in food and environmental matrices using a novel ratiometric NIR fluorescent sensor via carbonothioate-deprotection reaction. Environ. Pollut..

[128] Lv H.S., Sun H.Y. (2022). A novel coumarin-benzopyrylium based near-infrared fluorescent probe for Hg^2+^ and its practical applications. Spectrochim. Acta A Mol. Biomol. Spectrosc..

[129] Zhou H., Jin Y., Liu Y.K., Wang Y.X., Pang X.F., Zhao Q., Bu M. (2024). A near-infrared “turn-on” fluorescent probe based on dicyanomethylene-4H-pyran for the selective detection of Hg^2+^ in living cells. J. Photochem. Photobiol. A Chem..

[130] Zhang H., Cong L.M., Sun C., Zhang J.W., Guo S., Zhu S.H., Guo B.Z., Zhou J.Q., Zhang Y.L. (2025). A novel NIR fluorescent probe with large stokes shift for the detection of Hg^2+^ and its imaging in living cells and zebrafish. J. Fluoresc..

[131] Zhang H., Wu H.Y., Cong L.M., Dai A.J., Qu N., Guo S., Zhu S.H., Gou X.B., Zhang Y.L. (2025). Construction and application of a NIR benzothianone-based ratiometric fluorescent probe for visual detection of Hg^2+^ in biological systems and water samples. Anal. Chim. Acta.

[132] Liu X.-Y., Lin Y.-M., Hua F.-F., Fu Y.-L. (2025). Near-infrared fluorescent probe visual detection of Hg^2+^ and its application in biological system and ecological system. Spectrochim. Acta A Mol. Biomol. Spectrosc..

[133] Tang L., Dong Y., Cui M., Yao N., Wang Y. (2024). A triphenylamine based fluorescent probe for Zn^2+^ detection and its applicability in live cell imaging. Polyhedron.

[134] Wu Q., Feng L., Chao J.B., Wang Y., Shaung S. (2021). Ratiometric sensing of Zn(II) with a new benzothiazole-based fluorescent sensor and living cell imaging. Analyst.

[135] Shafique A. (2021). Removal of toxic pollutants from aqueous medium through adsorption: A review. Desalin. Water Treat..

[136] Li J., Zhou C.P., Yang H., Wu X.Z., Yan L.Q. (2022). Two near-infrared fluorescent probes based on dicyanoisfluorone for rapid monitoring of Zn^2+^ and Pb^2+^. Methods Appl. Fluoresc..

[137] Yan L.Q., Lu D.Q., Yang H., Wu X.Z. (2023). A dicyanisophorone-based probe for dual sensing Zn^2+^ and Cd^2+^ by near-infrared fluorescence. Spectrochim. Acta A Mol. Biomol. Spectrosc..

[138] Yan L.Q., Zhou C.P., Li J., Yang H., Wu X.Z., Li L. (2023). A near-infrared fluorescent probe based on dicyanisophorone for the detection of Zinc Ions (Zn^2+^) in water and living cells. J. Fluoresc..

[139] Song Q.X., Bai C.B., Dong Y.J., Chen M.Y., Wang S.Z., Hu J.D., Qiao X., Chen J., Li S.Y., Liu X.Y. (2024). Highly selective Zn^2+^ near-infrared fluorescent probe and its application in biological imaging. Spectrochim. Acta A Mol. Biomol. Spectrosc..

[140] Aydin D., Oguz M., Alici O., Erdemir S. (2024). Reversible NIR emitting fluorescent sensor with biocompatible and large Stokes shift for the Zn^2+^ in environmental samples and living cells. Measurement.

[141] Wang S.Z., Wang X., Xue W.H., Huo Y., Chen M.Y., Bai C.B., Song Q.X., Liu C.X., Zhang L., Qu C.Q. (2025). Qualitative and quantitative detection of Zn^2+^ in food: Design, synthesis and characterization of a near-infrared probe with large Stokes shift. Food Chem..

[142] Yan L.Q., Yang H., Li J., Zhou C.P., Li L., Wu X.Z., Lei C.H. (2022). A near infrared fluorescent probe for detection and bioimaging of zinc ions and hypochlorous acid. Anal. Chim. Acta.

[143] Wu L.Q., Liu Y., Wu X.D., Li Y.P., Du J.S., Qi S.L., Yang Q.B., Xu H., Li Y.X. (2022). A novel Near-Infrared fluorescent probe for Zn^2+^ and CN^-^ double detection based on dicyanoisfluorone derivatives with highly sensitive and selective, and its application in Bioimaging. Spectrochim. Acta A Mol. Biomol. Spectrosc..

[144] Yao Y.X., Zeng F.D., Ma J.L., Wu L.Q., Xing S., Yang H.Q., Li Y.P., Du J.S., Yang Q.B., Li Y.X. (2024). An innovative near-infrared fluorescent probe with FRET effect for the continuous detection of Zn^2+^ and PPi with high sensitivity and selectivity, and its application in bioimaging. Spectrochim. Acta A Mol. Biomol. Spectrosc..

[145] Liu L., Luo P.W., Guo X.S., Yang Y.H., Chen H., Chen X.X., Feng Y.T., Hu B., Xie Y., Yu M.H. (2026). A multifunctional dicyanoisophorone-based fluorescent probe for Zn^2+^ and pyrophosphate detection: Practical applications in water, living sprout, and cell imaging. Spectrochim. Acta A Mol. Biomol. Spectrosc..

[146] Zhang Y.B., Wang B.L., Rong X.Q., Liu J., Qiu X.Y., Sun L., Cheng X.T. (2023). Development of a novel near-infrared fluorescent probe for selective detection of zinc ions in environmental and food samples. Tetrahedron Lett..

[147] Jin H.B., Ju C., Duan C.X., Zhang N.W., Cao Y.Y., Xia Q.E., Zhou J., Gao S.M., Wang Y.G., Huang H. (2024). Revealing the elevation of Zn^2+^ in the brain of depressed mice by a ratiometric fluorescent probe with dual near-infrared emissions. Chem. Commun..

[148] Li Y., Li H., Fang W., Liu D., Liu M., Zheng M., Zhang L., Yu H., Tang H. (2022). Amplification of the fluorescence signal with clustered regularly interspaced short palindromic repeats-cas12a based on Au nanoparticle-DNAzyme probe and on-site detection of Pb^2+^ via the photonic crystal chip. ACS Sens..

[149] Lee J.Y., Mehta P.K., Sumita Subedi S., Keun-Hyeung Lee K.-H. (2023). Development of ratiometric fluorescent probes based on peptides for sensing Pb^2+^ in aquatic environments and human serum. Spectrochim. Acta A Mol. Biomol. Spectrosc..

[150] Bai C.B., Yao J.X., Meng Q., Dong Y.J., Chen M.Y., Liu X.Y., Wang X.Y., Qiao R., Huang H.N., Wei B. (2024). A near-infrared fluorescent ratiometric probe with large Stokes shift for multi-mode sensing of Pb^2+^ and bioimaging. J. Hazard. Mater..

[151] Liu Y.-T., Zhang Q.-Q., Yao S.-Y., Zhao K.-Y., Cui H.-W., Zhao H.-Y., Li J.-Y., Zou Y.-L., Zhao L.-X. (2025). A near-infrared multifunctional fluorescent bio-probe with large stokes shift and high quantum yield for effective determination of heavy metal lead and pesticide glyphosate in vitro and vivo. J. Hazard. Mater..

[152] Zhang Y.Y., Wei H.C., Li Y., Shang Z.Y., Zhang R., Zhang Z.Q., Meng Q.T. (2025). An azacrown ether-based near-infrared fluorescent probe for the detection of Pb^2+^ and its applications in food, environmental water, plant and animal samples. Anal. Chim. Acta.

[153] Xiao Y.L., Zhu J.Y., Xu H.Y., Liu Y.-G. (2025). Development of a novel isophorone-derived near-infrared fluorescent probe for specific and visual detection of Pb^2+^: Theoretical, practical and bioimaging investigations. J. Photochem. Photobiol. A Chem..

[154] Fan X.M., Qin L., Wang J.X., Zhan F.D., He Y.M., Zu Y.Q. (2019). A review of glutathione metabolism and cadmium tolerance in plants. J. West China Sci..

[155] Suhani I., Sahab S., Srivastava V., Singh R.P. (2021). Impact of cadmium pollution on food safety and human health. Curr. Opin. Toxicol..

[156] Tian G., Han Y.Z., Yang Q. (2023). A colorimetric and ratiometric fluorescence probe for Zn^2+^ and Cd^2+^ with 1, 10-phenanthroline derivative. J. Mol. Struct..

[157] Zu P.R., Yu Y.C., Li B.L., You J., Wu W.J., Wu M.Y. (2025). Visual detection of Cadmium and Glutathione by dicyanoisophorone fluorescent probe and its portable application. J. Fluoresc..

[158] Dutt S., Hamza I., Bartnikas T.B. (2022). Molecular mechanisms of Iron and heme metabolism. Annu. Rev. Nutr..

[159] Higashida K., Inoue S., Nakai N. (2020). Iron deficiency attenuates protein synthesisstimulated by branched-chain amino acids and insulin in myotubes. Biochem. Bioph. Res. Co..

[160] Liu C.Y., Rong X.D., Wang Y., Yan T.Y., Fu T.T., Sheng W.L., Zhu B.C. (2024). Rational construction of a novel fluorescent probe for imaging peroxynitrite in the endoplasmic reticulum during drug-induced liver injury. Sens. Actuators B Chem..

[161] Zhu X.Y., Lu H.Y., Jia H.N., Wei X.M., Xue J.W., Li W.J., Zhang J., Wang Y.L., Yan J.Y., Sun H.Y. (2025). Ferrostatin-1 reduces the inflammatory response of rheumatoid arthritis by decreasing the antigen presenting function of fibroblast-like synoviocytes. J. Transl. Med..

[162] Feng S.M., Zheng J.R., Zhang J.Z., Gui Z.S., Feng G.Q. (2022). Fe^2+^ imaging in ferroptosis and drug-induced liver injury with a ratiometric near-infrared fluorescent probe. Sens. Actuators B Chem..

[163] Xiang H.Y., Song Y.J., Wang Y.L., Fu W.Z., Xiao N. (2024). A novel NIR fluorescent probe for in situ visualizing Fe(II) and its application in drug-induced liver/kidney injury. Mater. Adv..

[164] Liu C.Y., Yan T.Y., Fu T.T., Wang K., Rong X.D., Liu X.T., Wang Y., Cai X.Y., Sheng W.L., Zhu B.C. (2025). A NIR fluorescent probe based on carbamoyl oxime with high specificity for detecting ferrous ions in food and in vivo. Food Res. Int..

[165] He Y.Q., Sun X.F., Yan X.M., Li Y., Zhong K.L., Tang L.J. (2022). A colorimetric, NIR, ultrafast fluorescent probe for ferric iron detection based on the PET mechanism and its multiple applications. J. Mater. Chem. C.

[166] Huang Y.R., Shen J.C., Yang L., Zhao G., Tian Q.Q., Ji N., Yuan J.L., Wang X.S., He W. (2025). A radical near-infrared fluorescent probe for imaging mitochondrial labile iron pool and evaluating ferroptosis inducers in ovarian cancer. Sens. Actuators B Chem..

[167] Mondal A., Mondal S., Majee A., Dutta S. (2025). An N-oxide-BODIPY-based ratiometric NIR fluorescent probe for the selective and sensitive detection of ferrous ions in real samples. New J. Chem..

[168] Luo P.W., Chen H., Cheng X.X., Liu L., Yang Y.H., Wen H.Y., Hu B., Zhou D., Jiang H.L. (2025). Colorimetric and fluorimetric detection of Fe(III) using a novel chromenylium-cyanine near-infrared fluorescence probe: Photophysical properties, DFT studies and cell imaging. Spectrochim. Acta A Mol. Biomol. Spectrosc..

[169] Yi M.R., Wang Y.T., Shen R.P., Ma T., Zhao J.T., Han F., Liu J.R., Li X.M., Zhang B.X., Fang J.G. (2026). A NIR fluorescent probe for imaging labile iron(II) pools in ferroptosis-related disorders. Sens. Actuators B Chem..

[170] Luo R.Q., Xu L., Chen J.M., Zhang W.X., Feng S.M., Qiu Z.P., Hong Y., Feng G.Q. (2025). Near-infrared fluorescent probe for simultaneously imaging Ferrous ions and viscosity in a mouse model of hepatocellular carcinoma. Anal. Chem..

